# Algorithm for optimized mRNA design improves stability and immunogenicity

**DOI:** 10.1038/s41586-023-06127-z

**Published:** 2023-05-02

**Authors:** He Zhang, Liang Zhang, Ang Lin, Congcong Xu, Ziyu Li, Kaibo Liu, Boxiang Liu, Xiaopin Ma, Fanfan Zhao, Huiling Jiang, Chunxiu Chen, Haifa Shen, Hangwen Li, David H. Mathews, Yujian Zhang, Liang Huang

**Affiliations:** 1 Baidu Research USA, Sunnyvale, CA USA; 2 School of EECS, Oregon State University, Corvallis, OR USA; 3 StemiRNA Therapeutics, Shanghai, China; 4 Department of Biochemistry and Biophysics, University of Rochester Medical Center, Rochester, NY USA; 5 Center for RNA Biology, University of Rochester Medical Center, Rochester, NY USA; 6 Department of Biostatistics and Computational Biology, University of Rochester Medical Center, Rochester, NY USA; 7 Coderna.ai, Inc., Sunnyvale, CA USA; 8 Present Address: Vaccine Center, School of Basic Medicine and Clinical Pharmacy, China Pharmaceutical University, Nanjing, China; 9 Present Address: Department of Pharmacy, National University of Singapore, Singapore, Singapore; 10 Present Address: Gaithersburg, MD USA

**Keywords:** Computational biology and bioinformatics, Computer science, RNA vaccines

## Abstract

Messenger RNA (mRNA) vaccines are being used to combat the spread of COVID-19 (refs. ^1–3^), but they still exhibit critical limitations caused by mRNA instability and degradation, which are major obstacles for the storage, distribution and efficacy of the vaccine products^4^. Increasing secondary structure lengthens mRNA half-life, which, together with optimal codons, improves protein expression^5^. Therefore, a principled mRNA design algorithm must optimize both structural stability and codon usage. However, owing to synonymous codons, the mRNA design space is prohibitively large—for example, there are around 2.4 × 10^632^ candidate mRNA sequences for the SARS-CoV-2 spike protein. This poses insurmountable computational challenges. Here we provide a simple and unexpected solution using the classical concept of lattice parsing in computational linguistics, where finding the optimal mRNA sequence is analogous to identifying the most likely sentence among similar-sounding alternatives^6^. Our algorithm LinearDesign finds an optimal mRNA design for the spike protein in just 11 minutes, and can concurrently optimize stability and codon usage. LinearDesign substantially improves mRNA half-life and protein expression, and profoundly increases antibody titre by up to 128 times in mice compared to the codon-optimization benchmark on mRNA vaccines for COVID-19 and varicella-zoster virus. This result reveals the great potential of principled mRNA design and enables the exploration of previously unreachable but highly stable and efficient designs. Our work is a timely tool for vaccines and other mRNA-based medicines encoding therapeutic proteins such as monoclonal antibodies and anti-cancer drugs^7,8^.

## Abstract (web summary)

An algorithm based on concepts established in computational linguistics enables rapid principled design of mRNA vaccines optimizing both structural stability and codon usage, resulting in improved half-life, protein expression and immune responses.

## Main

mRNA vaccines^9,10^ have been recognized as viable tools to limit the spread of COVID-19 owing to their scalable production, safety and efficacy^1–3^. However, mRNA molecules are chemically unstable and prone to degrade, which leads to insufficient protein expression^5^, and, in turn, compromised immunogenicity and druggability. This instability has also become a major obstacle in the storage and distribution of the vaccine, requiring the use of cold-chain technologies that hinders its use in developing countries^4^. Thus an mRNA molecule with enhanced stability is desirable, which would potentially have greater potency and favourable clinical efficacy.

Although it remains difficult to model chemical stability, previous work has established its correlation with RNA secondary structure, as quantified by the well-studied thermodynamic folding stability. Improving this structural stability, combined with optimal codon usage, leads to increased protein expression^5^. Therefore, a principled mRNA design algorithm must optimize two factors—structural stability and codon usage—to enhance protein expression.

**Fig. 1:**
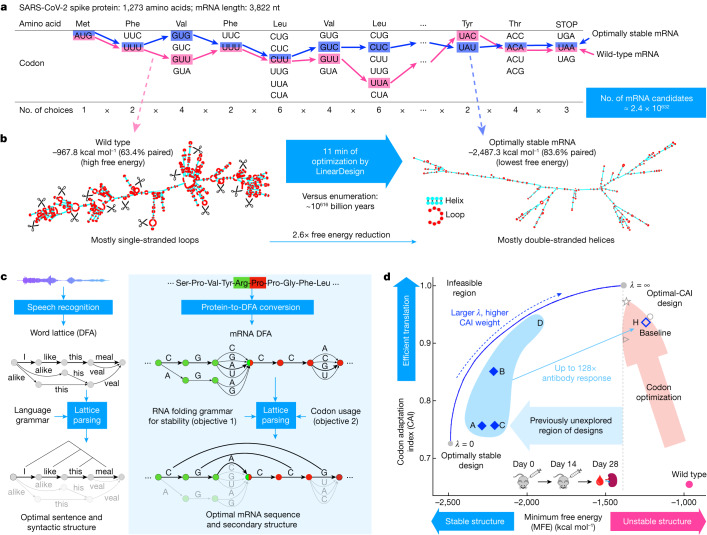
Overview of mRNA coding region design for both stability and codon optimality using SARS-CoV-2 spike protein as an example. **a**, Due to codon degeneracy and combinatorial explosion, there are around 2.4 × 10^632^ possible mRNA sequences encoding the spike protein. Enumerating every possible sequence would take around 10^616^ billion years. The pink and blue paths represent the wild-type and the optimally stable (lowest free energy) sequences, respectively. nt, nucleotides. **b**, The secondary structures of wild-type (left) and optimally stable (right) spike mRNAs. The wild-type mRNA is mostly single-stranded and thus prone to degradation in loop regions (red), whereas the optimally stable mRNA is mostly double-stranded. Optimization using LinearDesign takes around 11 min. **c**, The application of DFA and lattice parsing in computational linguistics (left) and its adaptation to mRNA design (right). An mRNA DFA (analogous to a word lattice) compactly encodes all mRNA candidates, which are folded simultaneously by lattice parsing to find the optimal mRNA (Fig. 2). **d**, Two-dimensional visualization of the mRNA design space, with stability (represented by MFE) on the *x* axis and codon optimality (represented by CAI) on the *y* axis. The standard mRNA design method of codon optimization improves codon usage (pink arrow) but is unable to explore the high-stability region (left of the dashed line); this standard approach is exemplified by the COVID-19 mRNA vaccine products BNT-162b2 (BioNTech-Pfizer, circle), mRNA-1273 (Moderna, star) and CVnCoV/CV2CoV (CureVac, wedge). LinearDesign jointly optimizes stability and codon optimality (blue curve, with *λ* being the weight assigned to codon optimality). We selected seven mRNA designs (four (A–D) are shown here) and a codon-optimized baseline (H) for in vitro and in vivo experiments (Fig. 4).

However, the mRNA design problem (we consider only the coding region in this work) is extremely challenging owing to the exponentially large search space. Each amino acid is encoded by a triplet codon—that is, three adjacent nucleotides—but owing to redundancies in the genetic code, most amino acids have multiple codons; there are 4^3^ (that is, 64) codons for the 20 common naturally occurring amino acids. This results in a prohibitively large number of candidates for any protein sequence. For example, the spike protein of SARS-CoV-2 has 1,273 amino acids and can therefore be encoded by approximately 2.4 × 10^632^ mRNA sequences (Fig. 1a). This poses an insurmountable computational challenge and rules out enumeration, which would take 10^616^ billion years for the spike protein (Fig. 1b). Conversely, codon optimization^11,12^, the conventional approach to mRNA design, optimizes codon usage but barely improves stability, leaving out the huge space of highly stable mRNAs. Optimizing GC content has a similar effect as it correlates with codon usage in vertebrates^13^. As a result, the vast majority of highly stable designs remains unexplored.

Here we describe LinearDesign, an algorithm that addresses this challenge by adapting the classical concept of lattice parsing^6^ in computational linguistics (Fig. 1c). We show that finding the optimal mRNA among the vast space of candidates is analogous to finding the most likely sentence among many similar-sounding alternatives. More specifically, we formulate the mRNA design space using a deterministic finite-state automaton (DFA), similar to a word lattice^6^, which compactly encodes exponentially many mRNA candidates. We then use lattice parsing to find the most stable mRNA in the DFA, or the optimal balance between stability and codon optimality in a weighted DFA. This unexpected connection to natural language enables an efficient algorithm that scales quadratically with the mRNA sequence length in practice. In this sense, our work transforms the enormous design space into an advantage—providing freedom of design—rather than an obstacle.

Compared to the codon-optimized benchmark, our COVID-19 and varicella-zoster virus (VZV) mRNA vaccines substantially improve chemical stability in vitro, protein expression in cells and immunogenicity in vivo. In particular, our COVID-19 vaccines achieved up to 128 times the antibody response of the codon-optimized benchmark in mice. This result reveals the great potential of principled mRNA design, and enables the exploration of these previously unreachable but highly stable and efficient designs. Our work provides a timely and promising tool for the design of mRNA vaccines and other mRNA-based medicines^14^ encoding therapeutic proteins including monoclonal antibodies^7^ and anti-cancer drugs^8^.

## Formulations and algorithms

**Extended Data Fig. 1:**
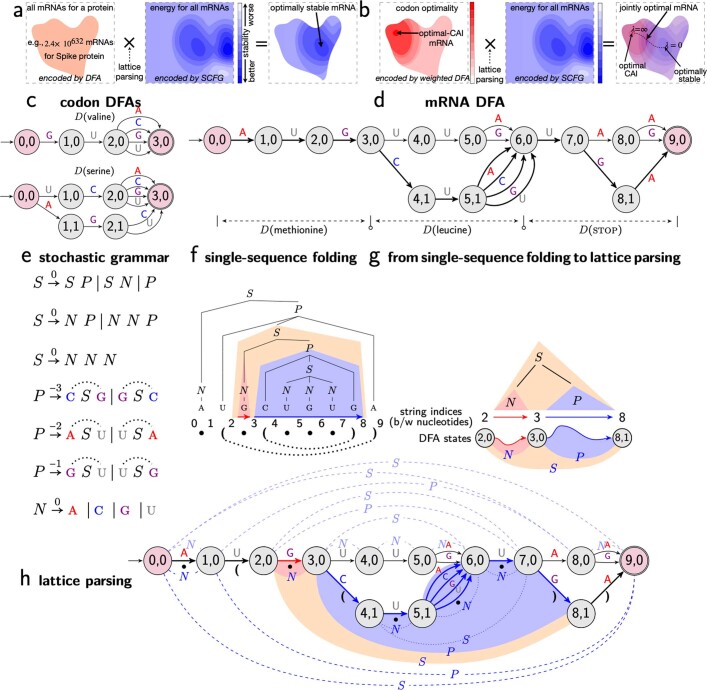
Illustrations of the optimization problems in mRNA design, DFA representations, single sequence folding as natural language parsing, and lattice parsing. **a–b**, Visualization of mRNA design as optimization problems for stability (objective 1, in **a**) and joint stability and codon optimality (objectives 1 and 2, in **b**). **c–h** show how lattice parsing solves the first optimization problem (see Fig. 2d for the second). **c**, Codon DFAs. **d**, An mRNA DFA made of three codon DFAs. The thick paths depict the optimal mRNA sequences under the simplified energy model in **e**, AUGCU⋆UGA, where ⋆ could be any nucleotide. **e**, Stochastic context-free grammar (SCFG) for a simplified folding free energy model. Each rule has a cost (i.e., energy term, the lower the better), and the dotted arcs represent base pairs in RNA secondary structure. **f**, Single-sequence folding is equivalent to context-free parsing with an SCFG; the parse tree represents the best secondary structure for the input mRNA sequence. **g**, We extend single-sequence parsing (top) to lattice parsing (bottom) by replacing the input string with a DFA, where each string index becomes a DFA state, and a span becomes a path between two states. **h**, Lattice parsing with the grammar in **e** for the DFA in **d**. The blue arcs below the DFA depict the (shared) best structure for the optimal sequences AUGCU⋆UGA in the whole DFA, while the dashed light-blue arcs above the DFA represent the best structure for a suboptimal sequence AUGUUAUAA. Lattice parsing can also incorporate codon optimality (objective 2, see **b**), by replacing the DFA with a weighted one (Fig. 2d).

Previous work^5^ established two main objectives for mRNA design, stability and codon optimality, which synergize to increase protein expression. To optimize for stability, given a protein sequence, we aim to find the mRNA sequence that has the lowest minimum-free-energy change (MFE) among all possible mRNA sequences encoding that protein; that is, for each candidate mRNA sequence, we find its MFE structure among all its possible secondary structures using the standard RNA folding energy model^15,16^ and then choose the sequence whose MFE energy is the lowest. This is thus a minimization within a minimization (Extended Data Fig. 1a). This method would take billions of years, thus an efficient algorithm without enumeration is needed.

We also aim to jointly optimize mRNA stability and codon optimality. Codon optimality is often measured by the codon adaptation index^17^ (CAI), which is defined as the geometric mean of the relative adaptiveness of each codon in the mRNA. Because CAI is between 0 and 1 but MFE is generally proportional to the sequence length, we multiply the logarithm of CAI by the number of codons in the mRNA and use the hyper-parameter CAI weight (*λ*) to balance MFE and CAI (*λ* = 0 being MFE-only). The combined objective is MFE – *λ|***p***|* log CAI, where *|***p***|* is the protein length. See Methods, ‘Optimization objectives’ and Extended Data Fig. 1b for details.

We next describe our solution to these two optimization problems with two ideas borrowed from natural language: DFA (lattice) representation and lattice parsing.

### Lattice representation for mRNA design space

**Fig. 2:**
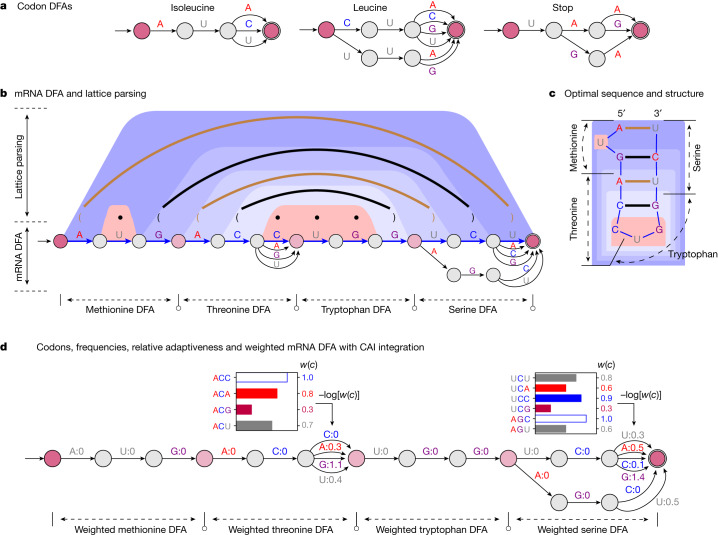
Illustration of the LinearDesign algorithm. **a**, Codon DFAs. **b**, An mRNA DFA (bottom) and lattice parsing on that DFA (top). In the DFA, the optimal mRNA sequence under a simplified energy model is shown as the blue path, together with its optimal structure shown in the dot-bracket format (dots indicate unpaired, and brackets indicate base pairs). In lattice parsing, the brown and black arcs also depict base pairs (two GC pairs and two AU pairs), and the trapezoidal shaded areas depict the decomposition of the optimal structure. Among all mRNA sequences encoded in the DFA, lattice parsing finds the optimal sequence with its optimal structure, achieving the lowest free energy under this energy model, where GC and AU pairs have energies of −3 and −2 kcal mol^−1^, respectively (Extended Data Fig. 1e). Note that here we use the simplified energy model for illustration, but our implementation uses the nearest-neighbour energy model. **c**, Another illustration of the optimal sequence and secondary structure in **b**. **d**, Joint optimization between stability and codon optimality by integrating the codon optimality in weighted DFAs. Top, the codon frequencies for threonine and serine. The relative adaptiveness *w*(*c*) of a codon *c* is the ratio of the frequency of *c* to the frequency of the most frequent codon encoding the same amino acid (white bar), and its value is shown to the right of the bars. Bottom, a weighted mRNA DFA encodes the CAI of each candidate in the total weight of its corresponding path by using –log[*w*(*c*)] (the cost of choosing codon *c*) as edge weights (Methods, ‘Optimization objectives’). This weighted DFA is used as input to lattice parsing for joint optimization between stability and codon optimality.

**Extended Data Fig. 2:**
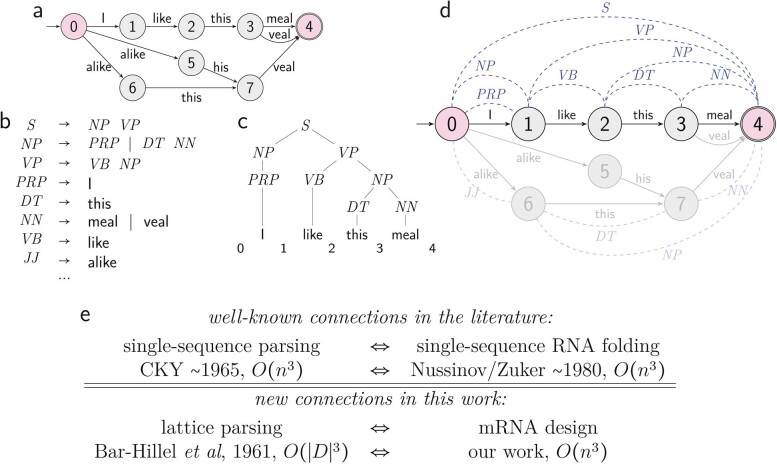
Word lattice and lattice parsing in natural language processing, and correspondence between linguistics and biology. **a**, An example of word lattice (sentence DFA) for speech recognition. **b**, Simplified language grammar. **c**, Single sentence parsing with between-word indices, which is a special case of word lattice parsing. **d**, Illustration of word lattice parsing for speech recognition with given word lattice and language grammar; the dashed blue arcs above the DFA depict the best parsing structure for the optimal sentence “I like this meal”, while the dashed light-blue arcs below the DFA represent the best parsing structure for a non-optimal sentence “alike this veal”. **e**, Correspondence between computational linguistics (left) and computational biology (right). See also Fig. 1.

Inspired by the word lattice representation of ambiguities in computational linguistics (Extended Data Fig. 2a), we represent the choice of codons for each amino acid using a similar lattice—more formally, a DFA, which is a directed graph with nucleotide-labelled edges (Fig. 2a and Extended Data Fig. 1c; see Methods, ‘DFA representations for codons and mRNA candidate sequences’ for formal definitions). After building a codon DFA for each amino acid in the protein sequence, we concatenate them into a single mRNA DFA, where each path between the start and final states represents a possible mRNA sequence encoding that protein (Fig. 2b and Extended Data Fig. 1d).

### Lattice parsing

**Fig. 3:**
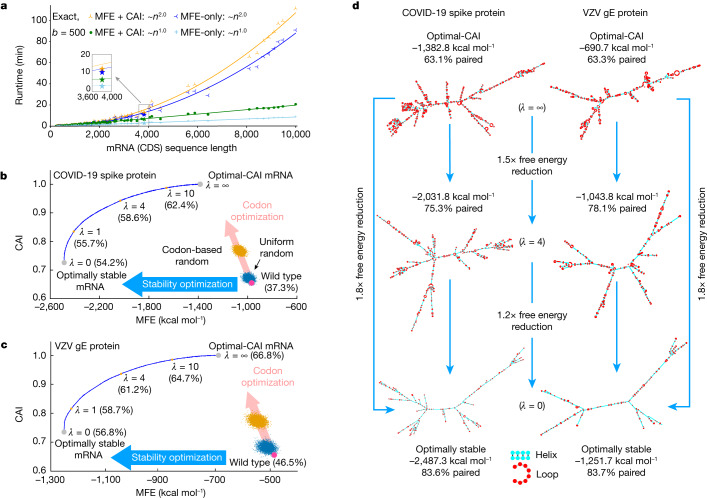
Computational characteristics of the LinearDesign algorithm. **a**, Runtime analysis of mRNA design for proteins in UniProt (Supplementary Table 1). Overall, our exact search scales quadratically with sequence length (Supplementary Figs. 7 and 8), and our MFE + CAI mode (with *λ* = 4) is about 15% slower than the MFE-only version. Moreover, beam search (*b* = 500) significantly speeds up the design of long sequences, with minor search errors (Supplementary Fig. 9). **b**,**c**, Two-dimensional (MFE–CAI) visualizations of designs for the SARS-CoV-2 spike (**b**) and VZV gE (**c**) proteins, respectively (both using human codon preference). The blue curves form the feasibility limit (optimal boundary), by varying *λ* from 0 to ∞ (see Extended Data Fig. 4 for *λ* of (–∞, 0]). The GC percentage is shown in parentheses. The human genome favours GC-rich codons; therefore, codon optimization (pink arrows) also improves stability, but only marginally, as the two optimization directions (codon versus stability) are largely orthogonal. By contrast, with an AU-rich codon preference (such as in yeast), codon optimization decreases stability (Extended Data Fig. 4b). **d**, Secondary structures of the mRNA designs for SARS-CoV-2 spike and VZV gE protein. The optimal-CAI designs (top, *λ* = ∞) are largely single-stranded (around 60% base-paired), whereas the optimally stable designs (bottom, *λ* = 0) are mostly double-stranded (around 80% base-paired). We also show intermediate designs (centre, *λ* = 4) with a balance of stability and CAI.

RNA folding is known to be equivalent to natural language parsing, where a stochastic context-free grammar (SCFG) can represent the folding energy model^18^ (Extended Data Fig. 1e,f). For mRNA design, the hard question is how all the mRNA sequences in the DFA can be folded together. We borrow the idea of lattice parsing^6,19^, which generalizes single-sequence parsing to handle all sentences in the lattice simultaneously to find the most likely one (Fig. 1c and Extended Data Fig. 2). Similarly, we use lattice parsing to fold all sequences in the mRNA DFA simultaneously to find the most stable one (Fig. 2b and Extended Data Fig. 1g,h). Note that lattice parsing is also an instance of dynamic programming, but over a much larger search space, and single-sequence folding can be viewed as a special case of lattice parsing with a single-chain DFA. This process can also be interpreted as the SCFG–DFA intersection (Extended Data Fig. 1a) where the SCFG scores for stability and the DFA demarcates the set of candidates. The runtime of this algorithm scales cubically with the mRNA sequence length (Methods, ‘SCFG, lattice parsing and intersection’), but for practical applications it scales quadratically (Fig. 3a).

### Lattice parsing with weighted DFAs

We now extend DFAs to weighted DFAs to integrate codon optimality on edge weights. Since our joint optimization formulation factors CAI onto the relative adaptiveness *w*(*c*) of each individual codon *c*, we set edge weights in each codon DFA so that a codon *c* has path cost –log *w*(*c*), which can be interpreted as the “amount of deviation” from the optimal codon. Then in a weighted mRNA DFA, the cost of each start-end path is the sum of –log *w*(*c*) for each codon *c* in the corresponding mRNA, which is proportional to its –log CAI (Fig. 2d). Now lattice parsing takes a stochastic grammar (for stability) and a weighted DFA (for codon usage) and solves the joint optimization with optimality guarantee, which can be viewed as the weighted intersection^20^ between an SCFG and a weighted DFA (Extended Data Fig. 1b and Methods, ‘Weighted DFA for CAI integration’).

### Expressiveness of DFAs

**Extended Data Fig. 3:**
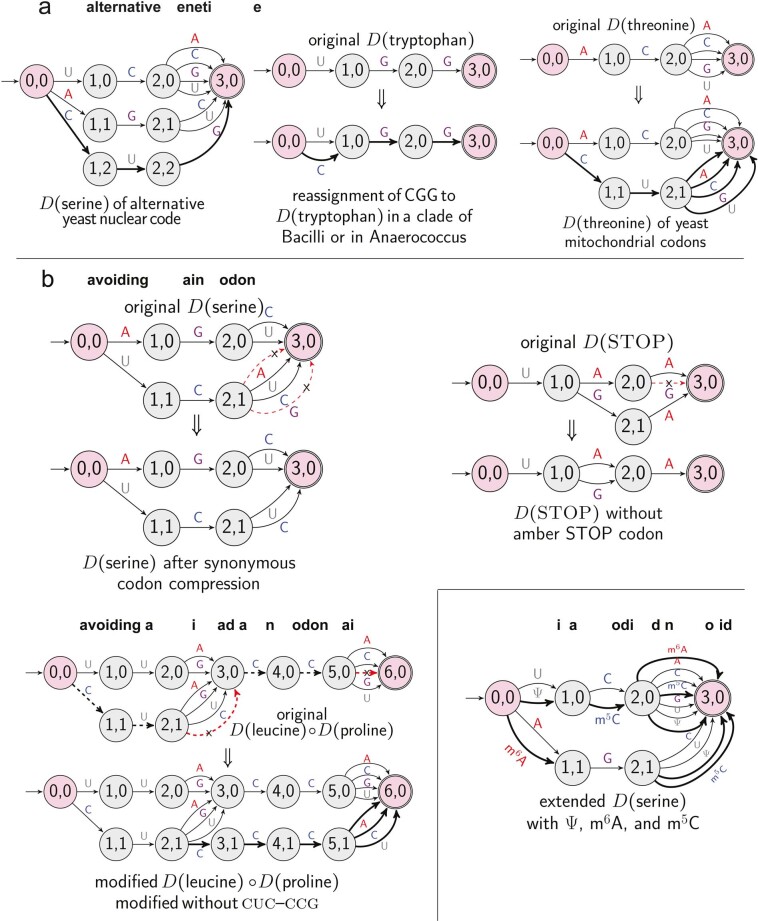
Examples of the DFA representations for extended codons, modified nucleotides, and coding constraints. **a**, Alternative genetic codes of serine, tryptophan, and threonine. **b**, Avoiding certain codon. On the left it shows the original DFA of serine (up), in which the red dashed arrows indicating UCA and UCG are chosen to be avoided, resulting in a new DFA (down). On the right it shows removing the rare amber STOP codon (UAG). **c**, Avoiding a specific adjacent codon pair. **d**, Extended serine DFA can include chemically modified nucleotides pseudouridine (Ψ), 6-Methyladenosine (m^6^A) and 5-methylcytosine (m^5^C).

Our DFA framework is sufficiently general that it can also represent alternative genetic codes, modified nucleotides and coding constraints. For details, see Methods, ‘DFAs for other genetic codes, coding constraints and modified nucleotides’, Extended Data Fig. 3 and Supplementary Fig. 5.

### Linear-time approximation

The exact design algorithm might still be slow for long sequences. Additionally, suboptimal designs may also be worth exploring for wet laboratory experiments, owing to the many other factors involved in mRNA design besides stability and codon usage. We therefore developed an approximate search version that runs in linear time using beam search, keeping only the top *b* most promising items per step (where *b* is the beam size), inspired by our previous work LinearFold^21^.

### Related work

**Fig. 4:**
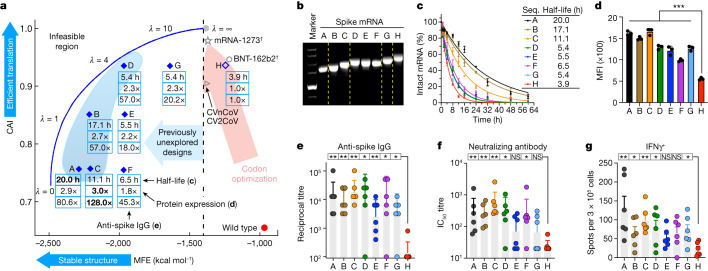
Experimental evaluation of LinearDesign-generated mRNA sequences encoding SARS-CoV-2 spike protein. **a**, Summary of chemical stability of and protein expression from spike mRNA designs A–G and the corresponding immune response (induction of anti-spike IgG) in mice compared to the codon-optimized benchmark H. The vaccines of mRNA-1273 and BNT-162b2 are annotated with daggers, because they use modified nucleotides, but their MFEs here are calculated with the standard energy model. **b**, Non-denaturing agarose gel characterization of mRNA, showing the correlation of gel mobility with minimum free energy. For gel source data, see Supplementary Fig. 1a. **c**, Chemical stability of mRNAs upon incubation in 10 mM Mg^2+^ buffer at 37 °C. Data are from three independent experiments. Seq., sequence. **d**, Protein expression levels from mRNAs 48 h after transfection into HEK293 cells, as determined by flow cytometry. Mean fluorescence intensity (MFI) values are derived from three independent experiments. Kruskal–Wallis ANOVA with Dunn’s multiple comparisons with the H group. **e**–**g**, C57BL/6 mice (*n* = 6) were immunized intramuscularly with two doses of formulated mRNA with a two-week interval. **e**, End-point titre of anti-spike IgG. **f**, Levels of neutralizing antibodies against wild-type SARS-CoV-2. IC_50_, half-maximal inhibitory concentration. **g**, Frequencies of IFNγ-secreting T cells, measured by enzyme-linked immunospot (ELISpot) assay. Two-tailed Mann–Whitney U test. Data are mean ± s.d. (**c**,**d**), geometric mean ± geometric s.d. (**e**,**f**) or mean ± s.e.m. (**g**). *P < 0.05, **P < 0.01, ***P < 0.001. NS, not significant. See Extended Data Figs. 5–7 and Supplementary Figs. 10 and 12 for extra experimental results and predicted secondary structures, and Supplementary Table 2 for detailed computational and experimental data. Source Data

**Fig. 5:**
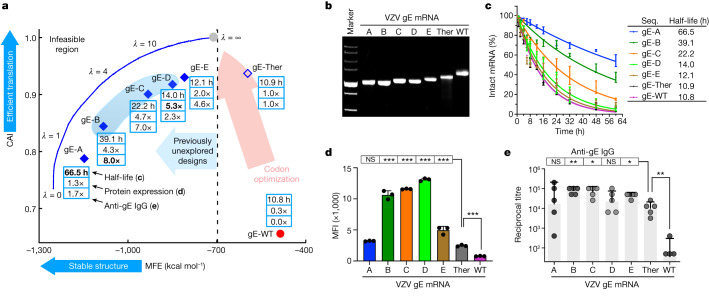
Experimental evaluation of LinearDesign-generated mRNAs encoding VZV gE protein. **a**, Summary of chemical stability of and protein expression from VZV gE mRNA designs and the corresponding immune response (induction of anti-gE IgG) in mice. The ‘sweet spot’ region is highlighted with light blue shading. **b**, Non-denaturing agarose gel characterization of mRNA showing the correlation of gel mobility with minimum free energy; for gel source data, see Supplementary Fig. 1b. **c**, Chemical stability of mRNAs upon incubation in 10 mM Mg^2+^ buffer at 37 °C. Data are from three independent experiments. **d**, Protein expression levels from mRNAs 48 h after transfection into HEK293 cells, as determined by flow cytometry. MFI values are derived from three independent experiments. Kruskal–Wallis ANOVA with Dunn’s multiple comparisons with the gE-Ther group. **e**, C57BL/6 mice (*n* = 5) were immunized intramuscularly with two doses of formulated mRNA with a two-week interval. End-point titre of anti-gE IgG is shown. Two-tailed Mann–Whitney U test. Data are mean ± s.d. (**c**,**d**) or geometric mean ± geometric s.d. (**e**). See Extended Data Fig. 8 for extra experimental results, Supplementary Fig. 11 for predicted secondary structures and Supplementary Table 3 for detailed computational and experimental data. Source Data

Two previous studies also tackled the problem of ‘most stable mRNA design’ (our objective 1) via dynamic programming, but using specialized extensions of the Zuker algorithm^22,23^ that cannot incorporate codon optimality (objective 2). By contrast, we established the connection between mRNA design and lattice parsing from computational linguistics. This connection enabled a simpler and more generalizable algorithm that can jointly optimize codon usage with a novel objective function that factors CAI onto individual codons. We also verified these algorithmic designs in vivo, showing substantial improvements for two mRNA vaccines (Figs. 4 and 5). See Methods, ‘The LinearDesign algorithm’ and ‘Related work’ for details.

## In silico results and analysis

Figure 3a benchmarks the runtime of LinearDesign on UniProt proteins^24^. LinearDesign was shown in a combination of two optimization objectives: MFE-only (objective 1) versus MFE and CAI (objectives 1 and 2), and two search modes: exact search versus beam search (*b* = 500). Empirically, LinearDesign scales quadratically with mRNA sequence length *n* for practical applications (*n* < 10,000 nt) thanks to the DFA representation and lattice parsing (Supplementary Figs. 7 and 8). Next, our CAI-integrated exact search (*λ* *=* 4) had the same empirical complexity, and was only around 15% slower than the MFE-only version thanks to the convenience of our DFA representation for adding CAI. Last, our beam search version (*b* = 500) further speeds up the design and scales linearly with sequence length, taking only 2.7 min (versus 10.7 min for exact search) on the SARS-CoV-2 spike protein (for MFE-only), with an approximation error (percentage energy gap, defined as (1 – MFE_approx_design_/MFE_exact_design_) × 100%) of 1.2%, where the subscripts indicate approximate or exact design. In fact, as sequences get longer, this percentage stabilizes, suggesting that beam search quality does not degrade with sequence length (Supplementary Fig. 9).

**Extended Data Fig. 4:**
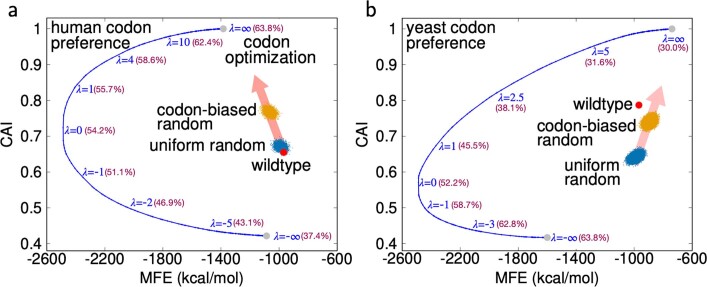
Two dimensional (MFE-CAI) visualizations of mRNA designs for the Spike protein using human codon preference (a) and yeast codon preference (b) with positive and negative λ’s. GC% are shown in parentheses. The human genome prefers GC-rich codons that lead to higher CAI designs are with higher GC%, while the yeast genome prefers AU-rich codons that exhibit an opposite relationship between CAI and GC%. See also Fig. 3 for more *in silico* results of LinearDesign.

For a GC-favouring codon preference (such as in humans), the conventional codon-optimization method does improve stability, but only slightly, since the codon-optimization direction (pink arrows) are largely orthogonal to the stability optimization direction (blue arrows) (Fig. 3b,c). By contrast, our LinearDesign can directly optimize stability and find the optimally stable mRNAs. On both the SARS-CoV-2 spike protein and the VZV gE protein, the lowest MFEs (*λ* = 0) are 1.8 times lower than the optimal CAIs (*λ* = ∞). Also, our optimally stable designs have mostly double-stranded secondary structures (Fig. 3d), which are predicted to be much less prone to degradation^5^. By varying *λ* from 0 to ∞, LinearDesign computes the feasibility limit (optimal boundary) of the mRNA design space (the blue curves in Fig. 3b,c; see Extended Data Fig. 4 for *λ* in (–∞, 0]). Furthermore, when the codon bias prefers AU-rich codons (such as in yeast), codon optimization actually worsens the stability (Extended Data Fig. 4b).

## Results for COVID-19 mRNA vaccines

We examined eight mRNA sequences for the SARS-CoV-2 spike protein in this study. Seven sequences (sequences A–G) were designed with the LinearDesign algorithm as suboptimal molecules (with beam search^21,25^). They were widely distributed in the low-MFE design space (the region where MFE ≤ −1,400 kcal mol^−1^ in Fig. 4a), which is unreachable with a conventional codon optimizing algorithm. To obtain a better understanding of the biological effects of MFE and CAI, we designed these mRNA sequences to have almost identical values in either MFE (B and C have similar MFEs, while D, E and F have similar MFEs) or CAI (A, C and F have similar CAIs, B and E have similar CAIs and D, G and H have similar CAIs). The eighth mRNA sequence (sequence H) was designed with OptimumGene, a widely used codon-optimization tool, as a benchmark. This benchmark sequence has been used in a COVID-19 mRNA vaccine that elicited high immunogenicity in two animal models^26^ and entered a phase I clinical trial in China (co-developed with the Chinese Center for Disease Control and Prevention; Chinese Clinical Trial Registry: CTR20210542). All of these mRNA sequences encode the same amino acid sequence of full-length wild-type SARS-CoV-2 spike protein, use natural unmodified nucleotides, and share the same 5′- and 3′-UTRs (see Supplementary Information for sequences).

Considering the potential negative effect on translation efficiency caused by a structured 5′-leader region^5^, we did not include the first 5 amino acids when running LinearDesign, and instead used a heuristic to select the first 15 nucleotides. It has also been suggested that long helices may elicit unwanted innate immune responses^27^, so we avoided them in our designs. This also explains why we did not study the lowest-MFE candidates (those closest to the optimal boundary—the blue curve in Fig. 4a), which usually contain long stems. See Methods, ‘Additional design constraints’ for details.

Besides coding region design, UTR structure is also crucial for translation^28^ and UTR engineering has a profound effect on protein expression^3^. Although LinearDesign does not address UTR optimization per se, its designed mRNA molecules—as they are more structured than solely codon-optimized ones—form fewer base pairs with and thus interfere less with the structures of widely used UTRs (Extended Data Table 1). This was confirmed by a different pair of UTRs in our VZV mRNA vaccine experiments (Extended Data Table 2) leading to improved protein expression and immune responses (Fig. 5). This evidence suggests that LinearDesign is likely to remain effective independent of the choice of UTRs, which is also consistent with a recent study^29^ in which LinearDesign-generated sequences with three different UTRs exhibited stronger in vitro protein expression over all benchmark sequences (see figure 4a in ref. ^29^); see Methods, ‘Related work’ for details.

### In-solution structure compactness and chemical stability

We then studied the structural compactness of mRNA molecules, which is hypothesized to be correlated with the folding free-energy change. An mRNA molecule with a lower MFE tends to contain more secondary structures, exhibit a more compact shape and have a smaller hydrodynamic size, resulting in a higher electrophoretic mobility. We loaded mRNA samples onto a non-denaturing agarose gel and found that RNA mobility rates correlated well with the calculated MFEs for sequences A–H (Fig. 4b) despite the sequences having similar molecular weights. Sequence A, with the lowest MFE, exhibited the highest mobility, followed by other sequences in order of their MFEs. Sequence H, which has the highest MFE value, was the least mobile. These data demonstrated the validity of the MFE calculation executed by LinearDesign.

**Extended Data Fig. 5:**
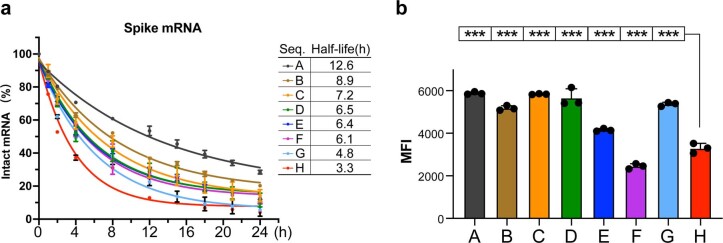
Extra experimental results of LinearDesign-generated mRNAs encoding the Spike protein. **a**, In-solution stability of sequences A–H in PBS buffer containing 20 mM Mg^2+^ at 37 °C over the course of 24 h. The degradation experiments were performed in triplicate independently and the data were presented as mean ± s.d. and fitted with a one-phase decay curve. **b**, Protein expression of mRNAs following transfection into HEK293 cells for 24 h was determined by flow cytometry. MFI values derived from three independent experiments are shown. Kruskal–Wallis analysis of variance (ANOVA) with Dunn’s multiple comparisons test to H group was performed for statistical analysis. ***P < 0.001. See Fig. 4c, d for similar experiments but with 10 mM Mg^2+^ and 48 h, respectively.

To evaluate the chemical stability of mRNAs, we incubated the mRNAs in buffers containing 10 mM (Fig. 4c) or 20 mM (Extended Data Fig. 5) Mg^2+^ at 37 °C, and assessed RNA integrity following incubation, similar to previous work^29^. Sequences A–H showed distinct degradation rates that correlated well with their MFEs (Fig. 4c and Extended Data Fig. 5). Sequence A, which has the lowest MFE, showed the slowest degradation rate, with a half-life (*T*_1/2_) of 20.0 and 12.6 h in 10 and 20 mM Mg^2+^ buffers, respectively (Fig. 4c and Extended Data Fig. 5). By contrast, sequence H, which has the highest MFE, degraded the fastest with *T*_1/2_ of 3.9 and 3.3 h in 10 and 20 mM Mg^2+^ buffers, respectively. These results support the idea that low-MFE designs are more resistant to in-solution degradation, a favourable trait for biological applications.

### Cellular protein expression

For vaccines, sufficient antigen expression is a key determinant for eliciting effective immune responses. We thus evaluated protein expression of the designed mRNAs. Sequences A–H were translated efficiently into spike protein following transfection in HEK293 cells. Of note, all seven mRNAs generated by LinearDesign (sequences A–G) showed remarkably higher protein expression levels than benchmark sequence H (Fig. 4d and Supplementary Fig. 12). Sequences D and G (with CAIs almost identical to H, but lower MFEs) expressed 2.3-fold higher protein levels than sequence H, and sequence A, with the lowest MFE, showed 2.9-fold higher expression. Collectively, our results are consistent with those of Mauger et al.^5^, which show that low MFE and high CAI synergize to improve protein expression; we were able to test this hypothesis using mRNA molecules with much lower MFE values, thanks to the ability of LinearDesign to explore the previously unreachable design space.

### In vivo immunogenicity

We further tested whether these designs could endow increased immunogenicity in vivo. We inoculated mRNA sequences A–H into mice using a lipid-based formulation^30^, and evaluated their humoral and cellular immune responses. For each mRNA sequence, C57BL/6 mice were inoculated intramuscularly with two doses of the vaccines with an interval of two weeks. Levels of anti-spike IgG, neutralizing antibodies and spike-specific interferon-γ (IFNγ)-secreting T cells were assessed. All mRNA molecules from LinearDesign were able to elicit robust antibody responses. By contrast, sequence H mRNA showed very limited ability to induce antibodies (Fig. 4e,f). Similar results were also observed on the antigen-specific T cell response, where a robust T helper 1-biased T cell response was induced only by the LinearDesign mRNAs (Fig. 4g). Sequences A–D, which are closer to the optimal boundary (blue shaded region in Fig. 4a), elicited a 57 to 128× increase in anti-spike IgG antibody titres and a 9 to 20× increase in neutralizing antibody titres over those elicited by the benchmark sequence H.

**Extended Data Fig. 6:**
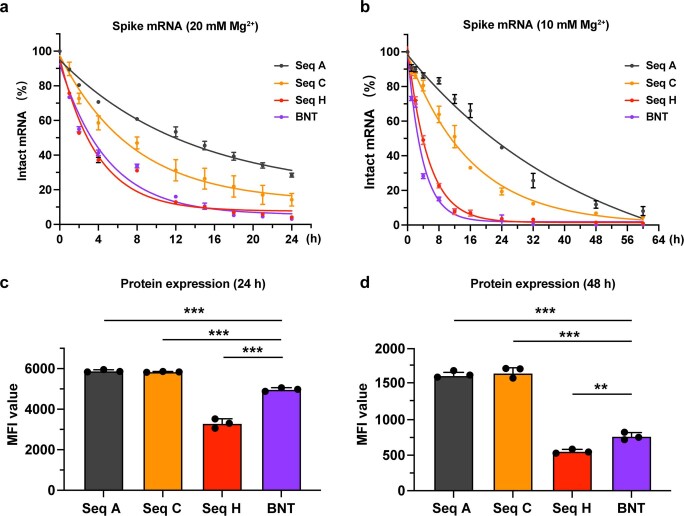
In-solution stability and protein expression of sequences A, C, H and BNT, for a head-to-head in vitro comparison between LinearDesign and BioNTech-Pfizer mRNA sequences. **a-b**, In-solution stability of mRNAs in PBS buffer containing 20 mM Mg^2+^ or 10 mM Mg^2+^ at 37 °C. Data are from three independent experiments and were presented as mean ± s.d. and fitted with one-phase decay curve. **c-d**, Protein expression of mRNAs was determined 24 h or 48 h following transfection into HEK293 cells. MFI value is presented as mean ± s.d. Each group has three independent assays and 10,000 live cells were collected for analysis in each assay. Kruskal–Wallis analysis of variance (ANOVA) with Dunn’s multiple comparisons test to BNT group was performed for statistical analysis. **P < 0.01, ***P < 0.001.

**Extended Data Fig. 7:**
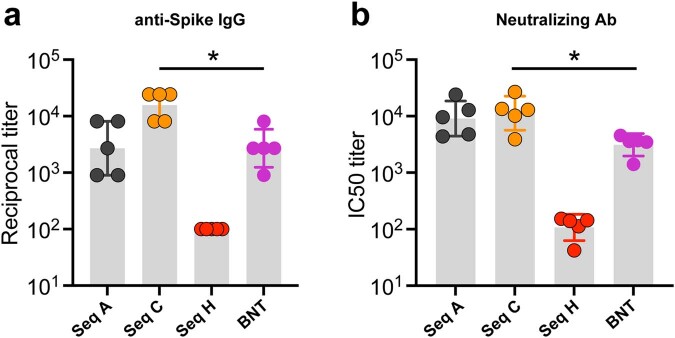
Antibody (Ab) responses induced by sequences A, C, H and BNT-based mRNA vaccines, for a head-to-head in vivo comparison between LinearDesign and BioNTech-Pfizer mRNA sequences. C57BL/6 mice (*n* = 5) were immunized *i.m*. with two doses of mRNA vaccines at a 2-week interval. Seven days after boost immunization, levels of anti-Spike IgG (**a**) and neutralizing Abs (**b**) against pseudotyped SARS-CoV-2 were measured. Data were presented as geometric mean ± geometric s.d. A two-tailed Mann-Whitney U test was used for statistical analysis. *P < 0.05. See Source Data for details. Source Data

Since BNT-162b2 from BioNTech-Pfizer is the most widely adopted COVID-19 mRNA vaccine, we compared it with the LinearDesign mRNAs. For this head-to-head comparison, our BNT sequence is almost identical to the sequence of BNT-162b2, but with three changes: (a) the two stabilizing proline mutations^31^ in BNT-162b2 converted back to the wild-type sequence, (b) BNT uses the same 5′- and 3′-UTRs as in sequences A–H, and (c) it uses natural unmodified nucleotides as in sequences A-H. Four mRNA sequences—A, C, H and BNT—were included in the study. A and C showed a markedly lower in-solution degradation rate and significantly higher protein expression in HEK293 cells than BNT (Extended Data Fig. 6). Note that BNT and H have very similar MFEs, CAIs (Fig. 4a) and half-lives. Moreover, A and C were able to elicit significantly higher levels of anti-spike IgG and neutralizing antibodies than H and BNT (Extended Data Fig. 7). Collectively, these data lead us to speculate that LinearDesign-optimized mRNA molecules are more stable in vivo, which leads to improved protein expression and enhanced immunogenicity.

## Results for VZV mRNA vaccines

**Extended Data Fig. 8:**
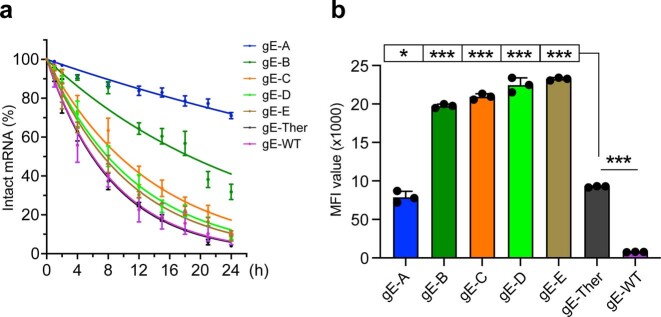
Extra stability and protein expression results of LinearDesign-generated mRNAs encoding VZV gE protein. **a**, In-solution stability of mRNAs upon incubation in buffer (Mg^2+^ = 20 mM) at 37 °C. Percentage of intact mRNA is shown. Data are presented as mean ± SD from three independent experiments. **b**, Protein expression of mRNAs following transfection into HEK293 cells for 24 h was determined by flow cytometry. Each group has three independent assays and 10,000 live cells were collected for analysis in each assay. MFI value is presented as mean ± s.d. Kruskal–Wallis analysis of variance (ANOVA) with Dunn’s multiple comparisons test to gE-Ther group was performed for statistical analysis. *P < 0.05, ***P < 0.001.

To further evaluate the generalizability of LinearDesign, we applied the algorithm to the design of a mRNA vaccine for VZV. Vaccination against VZV is considered an effective approach to reduce the risk of shingles^32^. Using the same strategy as for spike mRNA design (Fig. 4a), we generated five mRNA sequences encoding the full-length VZV gE protein (gE-A to gE-E). These sequences are widely distributed in the previously unexplored high-thermostability region (Fig. 5a). These sequences were benchmarked to the gE-Ther sequence, which we designed with the widely used codon-optimization tool GeneOptimizer^33^. These mRNAs, including wild-type gE mRNA (gE-WT), shared the same encoded amino acid sequence and 5′ and 3′ UTRs (the sequences are provided in Supplementary Information). In line with the spike mRNA data (Fig. 4b), gE-A mRNA, which has the lowest MFE, showed the greatest mobility in a non-denaturing gel (Fig. 5b) and markedly slower degradation rates with a *T*_1/2_ of 66.5 h in 10 mM (Fig. 5c) and 50.7 h in 20 mM (Extended Data Fig. 8a) Mg^2+^ buffers, indicating a high chemical stability correlated with the compactness of molecules. By contrast, gE-Ther showed a *T*_1/2_ of 10.9 h in 10 mM and 5.9 h in 20 mM Mg^2+^ buffers. We also observed that gE mRNA molecules were more stable than spike mRNAs owing to their shorter length^34^. In addition, protein expression from most of the LinearDesign-generated mRNAs (gE-B to gE-E) was significantly higher than for gE-Ther and gE-WT in HEK293 cells 48 h (Fig. 5d) and 24 h (Extended Data Fig. 8b) after transfection. However, the best-performing mRNA molecules were gE-B, gE-C and gE-D. They outperformed gE-A, which has the lowest CAI, and gE-E, which has the highest MFE. This emphasizes the importance of jointly optimizing CAI and MFE. The most highly expressed molecules were those whose CAI and MFE were both in the favourable region (light blue shaded area; Fig. 5a). Finally, we evaluated the immune respopnse elicited by VZV mRNA vaccines in C57BL/6 mice. LinearDesign mRNA molecules (gE-B, gE-C and gE-E) induced significantly higher levels of anti-gE IgG than gE-Ther or gE-WT (Fig. 5e).

## Discussion

An effective mRNA design strategy is of utmost importance for the development of mRNA vaccines, which have shown great promise against the COVID-19 pandemic. However, this task remains challenging owing to the prohibitively large search space. Here we present a simple solution by reducing the mRNA design problem to the classical problem of lattice parsing used in computational linguistics. This work resulted in an efficient algorithm that can design an optimal mRNA encoding the SARS-CoV-2 spike protein in 11 min. It can also jointly optimize stability and codon usage, which has been shown to be crucial for mRNA design. This approach is one of several recent fruitful exchanges between linguistics and biology^35,36^.

Here we have comprehensively characterized mRNA sequences generated by LinearDesign and demonstrated their superiority over the commonly used codon-optimization benchmark using two viral antigens across three attributes that are critical for vaccine performance: chemical stability, protein translation and in vivo immunogenicity. In particular, our designs for mRNA encoding the SARS-CoV-2 spike protein showed an increase of up to 128-fold in binding antibody levels over the codon-optimization benchmark. Our VZV gE mRNA designs—using a different UTR pair—also showed substantial improvements over the benchmark. These results indicate the robustness of LinearDesign in optimizing the coding region independently of UTR pairs. Indeed, coding region design and UTR engineering^3^ are complementary and could be combined in future work. It is worth noting that our designed mRNAs did not use chemical modification which is widely believed to be critical to the recent success of mRNA vaccines^1,2,10,37,38^, yet still showed high levels of stability, translation efficiency and immunogenicity, with the additional advantage of a lower manufacturing cost. The LinearDesign approach is likely to complement chemical modification and can be easily adapted to modified nucleotides once the corresponding energy model is available. Our work has only considered stability and codon usage but, owing to the generalizability of the lattice representation, could also be adapted to optimize other parameters relevant to mRNA design. By opening up the previously inaccessible region of highly stable and efficient sequences, this approach provides a timely and promising tool for mRNA vaccine development that is likely to have a key role in future pandemics. It is also a principled method for molecule design in the field of mRNA medicines generally, and can be used for all therapeutic proteins including monoclonal antibodies and anti-cancer drugs.

## Methods

### The LinearDesign algorithm

#### Optimization objectives

There are two objectives in mRNA design: stability and codon optimality. The optimal-stability mRNA design problem can be formalized as follows. Given a protein sequence ${\bf{p}}={p}_{0}\ldots {p}_{\left|p\right|-1}$ where each ${p}_{i}$ is an amino acid residue, we find the optimal mRNA sequence ${{\bf{r}}}^{* }\left({\bf{p}}\right)$ that has the lowest MFE among all possible mRNA sequences encoding that protein:

1 $${{\bf{r}}}^{\ast }({\bf{p}})={\rm{a}}{\rm{r}}{\rm{g}}{\rm{m}}{\rm{i}}{{\rm{n}}}_{{\bf{r}}\in {\rm{m}}{\rm{R}}{\rm{N}}{\rm{A}}({\bf{p}})}{\rm{M}}{\rm{F}}{\rm{E}}({\bf{r}})$$

2 $${\rm{M}}{\rm{F}}{\rm{E}}({\bf{r}})={\rm{m}}{\rm{i}}{{\rm{n}}}_{{\bf{s}}\in {\rm{s}}{\rm{t}}{\rm{r}}{\rm{u}}{\rm{c}}{\rm{t}}{\rm{u}}{\rm{r}}{\rm{e}}{\rm{s}}({\bf{r}})}\Delta G^\circ ({\bf{r}},{\bf{s}})$$

where ${\rm{mRNA}}\left({\bf{p}}\right)=\{{\bf{r}}| {\rm{protein}}\left({\bf{r}}\right)={\bf{p}}\}$ is the set of candidate mRNA sequences, ${\rm{structures}}\left({\bf{r}}\right)$ is the set of all possible secondary structures for mRNA sequence ${\bf{r}}$, and $\Delta G^\circ ({\bf{r}},{\bf{s}})$ is the free-energy change of structure ${\bf{s}}$ for mRNA ${\bf{r}}$ according to an energy model. This is clearly a double minimization objective involving the per-sequence minimization over all of its possible structures (that is, RNA folding; equation (2)), which has well-known dynamic programming solutions, and the global minimization over all sequences (that is, optimal mRNA design; equation (1)) which we will solve using lattice parsing (see ‘SCFG, lattice parsing and intersection’).

Next, we integrate codon optimality by adding CAI^17^, defined as the geometric mean of the codon optimality of each codon in the mRNA ${\bf{r}}$:

3 $${\rm{C}}{\rm{A}}{\rm{I}}({\bf{r}})=\sqrt[\frac{|{\bf{r}}|}{3}]{{\prod }_{0\le i < \frac{|{\bf{r}}|}{3}}w({\rm{c}}{\rm{o}}{\rm{d}}{\rm{o}}{\rm{n}}({\bf{r}},i))}$$

where ${\rm{c}}{\rm{o}}{\rm{d}}{\rm{o}}{\rm{n}}({\bf{r}},i)={r}_{3i}{r}_{3i+1}{r}_{3i+2}$ is the $i\text{th}$ triplet codon in ${\bf{r}}$, and $w\left(c\right)$ is the relative adaptiveness of codon $c$, defined as the frequency of $c$ divided by the frequency of its most frequent synonymous codon $\left(0\le w\left(c\right)\le 1\right)$. Because CAI is always between 0 and 1 but MFE is generally proportional to the mRNA sequence length, we scale CAI by the number of codons and use a hyper-parameter $\lambda$ to balance MFE and CAI ($\lambda =0$ being purely MFE), and define a novel joint objective:

4 $$\text{MFECA}{{\rm{I}}}_{\lambda }\left({\bf{r}}\right)=\text{MFE}\left({\bf{r}}\right)-\frac{\left|{\bf{r}}\right|}{3}\lambda {\rm{\log }}{\rm{CAI}}\left({\bf{r}}\right)$$

which can be simplified by expanding CAI:

5 $$\begin{array}{cc}{{\rm{M}}{\rm{F}}{\rm{E}}{\rm{C}}{\rm{A}}{\rm{I}}}_{\lambda }({\bf{r}}) & \,={\rm{M}}{\rm{F}}{\rm{E}}({\bf{r}})-\frac{|{\bf{r}}|}{3}\lambda \,\log \,\sqrt[\frac{|{\bf{r}}|}{3}]{{\prod }_{0\le i < \frac{|{\bf{r}}|}{3}}\,w({\rm{c}}{\rm{o}}{\rm{d}}{\rm{o}}{\rm{n}}({\bf{r}},i))}\\ \, & \,=\,{\rm{M}}{\rm{F}}{\rm{E}}({\bf{r}})-\lambda \sum _{0\le i < \frac{|{\bf{r}}|}{3}}\,\log \,w({\rm{c}}{\rm{o}}{\rm{d}}{\rm{o}}{\rm{n}}({\bf{r}},i))\end{array}$$

This joint objective is basically MFE plus (a scaled) sum of the negative logarithm of each codon’s relative adaptiveness. Now the joint optimization can be defined as:

6 $$\begin{array}{cc}{{\bf{r}}}_{\lambda }^{\ast }({\bf{p}}) & \,={{\rm{a}}{\rm{r}}{\rm{g}}{\rm{m}}{\rm{i}}{\rm{n}}}_{{\bf{r}}\in {\rm{m}}{\rm{R}}{\rm{N}}{\rm{A}}({\bf{p}})}{{\rm{M}}{\rm{F}}{\rm{E}}{\rm{C}}{\rm{A}}{\rm{I}}}_{\lambda }({\bf{r}})\\ \, & \,=\,{{\rm{a}}{\rm{r}}{\rm{g}}{\rm{m}}{\rm{i}}{\rm{n}}}_{{\bf{r}}\in {\rm{m}}{\rm{R}}{\rm{N}}{\rm{A}}({\bf{p}})}({\rm{M}}{\rm{F}}{\rm{E}}({\bf{r}})-\lambda \sum _{0\le i < \frac{|{\bf{r}}|}{3}}\,\log \,w({\rm{c}}{\rm{o}}{\rm{d}}{\rm{o}}{\rm{n}}({\bf{r}},i)))\end{array}$$

See Fig. 2d for examples of relative adaptiveness calculation.

#### DFA representations for codons and mRNA candidate sequences

Informally, a DFA is a directed graph with labelled edges and distinct start and end states. For our purpose each edge is labelled by a nucleotide, so that for each codon DFA, each start-to-end path represents a triplet codon; see Fig. 2a and Extended Data Fig. 1c for examples. Formally, a DFA is a 5-tuple $\left\langle Q,\varSigma ,\delta ,{q}_{0},F\right\rangle$, where $Q$ is the set of states, $\varSigma$ is the alphabet (here $\varSigma =\{\text{A},\text{C},\text{G},\text{U}\}$), ${q}_{0}$ is the start state (always $\left(\mathrm{0,0}\right)$ in this work), $F$ is the set of end states (in this work the end state is unique—that is, $F=\{\left(\mathrm{3,0}\right)\}$), and $\delta$ is the transition function that takes a state $q$ and a symbol $a\in \varSigma$ and returns the next state ${q}^{{\prime} }$—that is, $\delta \left(q,a\right)={q}^{{\prime} }$ encodes a labelled edge $q\mathop{\to }\limits^{a}{q}^{{\prime} }$.

After building DFAs for each amino acid, we can concatenate them (concatenation is indicated by $\circ$ below) into a single DFA $D\left({\bf{p}}\right)$ for a protein sequence ${\bf{p}}$, which represents all possible mRNA sequences that translate into that protein

$$D\left({\bf{p}}\right)=D\left({p}_{0}\right)\,\circ \,D\left({p}_{1}\right)\,\circ \,\cdots \,\circ \,D\left({p}_{\left|{\bf{p}}\right|-1}\right)\,\circ \,D({\rm{STOP}})$$

by stitching the end state of each DFA with the start state of the next. See Extended Data Fig. 1d for examples. The new end state of the mRNA DFA is $\left(3\left|{\bf{p}}\right|+\mathrm{3,0}\right)$.

We also define ${\rm{out}}\_{\rm{edges}}\left(q\right)$ to be the set of outgoing edges from state $q$, and ${\rm{in}}\_{\rm{edges}}\left(q\right)$ to be the set of incoming edges (which will be used in the pseudocode; Supplementary Figs. 2 and 3):

$$\begin{array}{c}{\rm{out}}\_{\rm{edges}}(q)=\{q\mathop{\to }\limits^{a}{q}^{{\prime} }| \delta (q,a)={q}^{{\prime} }\}\\ {\rm{in}}\_{\rm{edges}}(q)=\{{q}^{{\prime} }\mathop{\to }\limits^{a}q| \delta ({q}^{{\prime} },a)=q\}\end{array}$$

For the mRNA DFA in Extended Data Fig. 1d, out_edges ((3,0)) = $$\{(3,0)\mathop{\to }\limits^{{\rm{U}}}(4,0),(3,0)\mathop{\to }\limits^{{\rm{C}}}(4,1)\}$$ and in_edges ((9,0)) = $$\{(8,0)\mathop{\to }\limits^{{\rm{A}}}(9,0),$$$$(8,0)\mathop{\to }\limits^{{\rm{G}}}(9,0),(8,1)\mathop{\to }\limits^{{\rm{A}}}(9,0)\}$$.

#### SCFG, lattice parsing and intersection

A SCFG is a context-free grammar in which each rule is augmented with a weight. More formally, an SCFG is a 4-tuple $$\left\langle N,\varSigma ,P,S\right\rangle $$ where $$N$$ is the set of non-terminals, $$\varSigma $$ is the set of terminals (identical to the alphabet in the DFA, in this case $$\varSigma =\{\text{A},\text{C},\text{G},\text{U}\}$$), $$P$$ is the set of weight-associated context-free writing rules, and $$S\in N$$ is the start symbol. Each rule in $$P$$ has the form $$A\mathop{\to }\limits^{w}{(N\cup \varSigma )}^{* }$$ where $$A\in N$$ is a non-terminal that can be rewritten according to this rule into a sequence of non-terminals and terminals (the star $$* $$ means repeating zero or more times) and $$w\in {\mathbb{R}}$$ is the weight associated with this rule.

SCFGs can be used to represent the RNA folding energy model^39^. The weight of a derivation (parse tree, or a secondary structure in this case) is the sum of weights of the productions used in that derivation. For example, for a very simple Nussinov–Jacobson-style model^40^, which simplifies the energy model to individual base pairs, we can define this SCFG $G$ as in Extended Data Fig. 1e, where each GC pair gets a score of −3, and each AU pair gets a score of −2. Thus, the standard RNA secondary structure prediction problem can be cast as a parsing problem: given the above SCFG $G$ and an input RNA sequence, find the minimum-weight derivation in $G$ that can generate the sequence. This can be solved by the classical CKY algorithm from computational linguistics^41–43^.

The optimal-stability mRNA design problem is now a simple extension of the above single-sequence folding problem to the case of multiple inputs: instead of finding the minimum-free-energy structure (minimum-weight derivation) for a given sequence, we find the minimum-free-energy structure (and its corresponding sequence) among all possible structures for all possible sequences (Extended Data Fig. 1). This can be solved by lattice parsing on the DFA, which is a generalization of CKY from a single sequence to a DFA. Take the bifurcation rule $S\to NP$ for example. In CKY, if you have derived non-terminal $N$ for span $\left[i,j\right]$, notated $i\mathop{\to }\limits^{\genfrac{}{}{0ex}{}{N}{\triangle }}j$, and if you have also derived $j\mathop{\to }\limits^{\genfrac{}{}{0ex}{}{P}{\triangle }}k$, you can combine the two spans—that is, $i\mathop{\to }\limits^{\genfrac{}{}{0ex}{}{N}{\triangle }}j\mathop{\to }\limits^{\genfrac{}{}{0ex}{}{P}{\triangle }}k$—and use the above rule to derive $i\mathop{\to }\limits^{\genfrac{}{}{0ex}{}{S}{\triangle }}k$. Similarly, in lattice parsing, if you have derived both ${q}_{i}\mathop{\rightsquigarrow}\limits^{\genfrac{}{}{0ex}{}{N}{{\rm{\triangle }}}}{q}_{j}$ (that is, there is a ${q}_{i}\rightsquigarrow{q}_{j}$ path that can be derived from $N$) and ${q}_{j}\mathop{\rightsquigarrow}\limits^{\genfrac{}{}{0ex}{}{P}{{\rm{\triangle }}}}{q}_{k}$, you can combine them to a longer path ${q}_{i}\mathop{\rightsquigarrow}\limits^{\genfrac{}{}{0ex}{}{N}{{\rm{\triangle }}}}{q}_{j}\mathop{\rightsquigarrow}\limits^{\genfrac{}{}{0ex}{}{P}{{\rm{\triangle }}}}{q}_{k}$ and derive ${q}_{i}\mathop{\rightsquigarrow}\limits^{\genfrac{}{}{0ex}{}{S}{{\rm{\triangle }}}}{q}_{k}$ with the above rule. While the runtime for CKY scales $O\left(\left|G\right|{n}^{3}\right)$ where $\left|G\right|$ is the grammar constant (the number of rules) and $n$ is the RNA sequence length, the runtime for lattice parsing similarly scales $O\left(\left|G\right|{\left|D\right|}^{3}\right)$ where $|D|$ is the number of states in the DFA. For mRNA design with the standard genetic code, $n\le \left|D\right|\le 2n$ because each position $i$ has either one or two states ($\left(i,0\right)$ and $\left(i,1\right)$), so its time complexity is also actually identical to single-sequence folding, just with a larger constant. See ‘Left-to-right dynamic programming’ for details of this algorithm and Supplementary Figs. 2 and 3 for the pseudocode.

More formally, in theoretical computer science, lattice parsing with an CFG $G$ on a DFA $D$ is also known as the intersection between the languages of $G$ and $D$ (that is, the sets of sequences allowed by $G$ and $D$), notated $L\left(G\right)\cap L\left(D\right)$, which was solved by the Bar-Hillel construction in 1961 (ref. ^19^). In order to adapt it to mRNA design, we need to extend this concept to the case of weighted (that is, stochastic) grammars and weighted DFAs (the latter is needed for CAI integration; see below). While the language $L\left(G\right)$ of CFG $G$ is the set of sequences generated by $G$, the language of the SCFG for RNA folding free-energy model defines a mapping from each RNA sequence to its MFE—that is, ${L}_{w}\left(G\right):{\varSigma }^{* }\longmapsto {\mathbb{R}}$. This can be written as a relation:

$${L}_{w}\left(G\right)=\{{\bf{r}} \sim \text{MFE}\left({\bf{r}}\right){\rm{|}}{\bf{r}}\in {\varSigma }^{* }\}$$

And we also extend the language of a DFA to a trivial weighted language (which will facilitate the incorporation of CAI into DFA below):

$${L}_{w}\left(D\right)=\{{\bf{r}} \sim 0{\rm{|}}{\bf{r}}\in L\left(D\right)\}$$

Next we extend the intersection from two sets to two weighted sets *A* and *B*:

$$A{\cap }_{w}B=\{{\bf{r}} \sim \left({w}_{1}+{w}_{2}\right){\rm{|}}{\bf{r}} \sim {w}_{1}\in A,{\bf{r}} \sim {w}_{2}\in B\}$$

Now we can show that optimal-stability mRNA design problem can be solved via weighted intersection between ${L}_{w}\left(G\right)$ and ${L}_{w}\left(D\right)$—that is, we can construct a new ‘intersected’ stochastic grammar ${G}^{{\prime} }$ that has the same weights (that is, energy model) as the original grammar but only generates sequences in the DFA:

$${L}_{w}\left({G}^{{\prime} }\right)={L}_{w}\left(G\right){\cap }_{w}{L}_{w}\left(D\right)=\{{\bf{r}} \sim {\rm{MFE}}\left({\bf{r}}\right){\rm{| }}{\bf{r}}\in L\left(D\right)\}$$

#### Weighted DFA for CAI integration

As described in the main text and Fig. 2d, our novel joint optimization objective (equation (6)) factors the CAI of each mRNA candidate onto the relative adaptiveness of each of its codons, and thus can be easily incorporated into the DFA as edge weights. To do this we need to extend the definition of DFA to weighted DFA, where the transition function $\delta$ now returns a state and a weight—that is, $\delta \left(q,a\right)=\left({q}^{{\prime} },w\right)$—which encodes a weighted label edge $q\mathop{\to }\limits^{a:w}{q}^{{\prime} }$. Now the set of outgoing and incoming edges are also updated to:

$$\begin{array}{c}{\rm{out}}\_{\rm{edges}}(q)=\{q\mathop{\to }\limits^{a:w}{q}^{{\prime} }| \delta (q,a)=({q}^{{\prime} },w)\}\\ {\rm{in}}\_{\rm{edges}}(q)=\{{q}^{{\prime} }\mathop{\to }\limits^{a:w}q| \delta ({q}^{{\prime} },a)=(q,w)\}\end{array}$$

In this case, the weighted DFA defines a mapping from each candidate mRNA sequence to its negative logarithm of CAI scaled by the number of codons—that is, ${L}_{w}\left(D\right):L\left(D\right)\mapsto {\mathbb{R}}$. More formally,

$${L}_{w}\left(D\right):\{{\bf{r}} \sim -\frac{\left|{\bf{r}}\right|}{3}{\rm{\log }}\text{CAI}({\bf{r}}){\rm{|}}{\bf{r}}\in L(D)\}$$

Now the weighted intersection defined above can be extended to incorporate the hyper-parameter $\lambda$ and derive the joint objective:

$${L}_{w}^{\lambda }\left({G}^{{\prime} }\right)={L}_{w}(G){\cap }_{w}^{\lambda }{L}_{w}(D)=\{{\bf{r}} \sim (\text{MFE}({\bf{r}})-\lambda \frac{\left|{\bf{r}}\right|}{3}{\rm{\log }}\text{CAI}({\bf{r}})){\rm{|}}{\bf{r}}\in L(D)\}$$

#### Bottom-up dynamic programming

Next, we describe how to implement the dynamic programming algorithm behind lattice parsing (or equivalently, intersection between the languages of a SCFG and a weighted DFA) to solve the joint optimization problem. For simplicity reasons, here we use bottom-up dynamic programming on a modified Nussinov–Jacobson energy model. Supplementary Fig. 2 gives the pseudocode for this simplified version. We first build up the mRNA DFA for the given protein, and initialize two hash tables, ‘best’ to store the best score of each state, and ‘back’ to store the best backpointer. For the base cases $(S\mathop{\to }\limits^{0}N\,N\,N)$ we set ${\rm{best}}\left[S,{q}_{i},{q}_{i+3}\right]\leftarrow 0$ for optimal-stability design, and ${\rm{best}}\left[S,{q}_{i},{q}_{i+3}\right]\leftarrow {\rm{mincost}}\left({q}_{i},{q}_{i+3},\lambda \right)$ for the joint optimization where

7 $${\rm{mincost}}({q}_{i},{q}_{i+3},\lambda )\triangleq \mathop{\min }\limits_{{q}_{i}\mathop{\longrightarrow }\limits^{a:{w}_{1}}q{\prime} \mathop{\longrightarrow }\limits^{b:{w}_{2}}q{\prime\prime} \mathop{\longrightarrow }\limits^{c:{w}_{3}}{q}_{i+3}}\lambda ({w}_{1}+{w}_{2}+{w}_{3})$$

is the minimum ($\lambda$-scaled) cost of any ${q}_{i}\rightsquigarrow{q}_{i+3}$ path in the CAI-integrated DFA. Next, for each state $\left({q}_{i},{q}_{j}\right)$ it goes through the pairing rule and bifurcation rules, and updates if a better score is found. After filling out the hash tables bottom-up, we can backtrace the best mRNA sequence stored with the backpointers. See Supplementary Fig. 3 for details of UPDATE and BACKTRACE functions.

#### Left-to-right dynamic programming

Inspired by our previous work, LinearFold^21^, we further developed a left-to-right dynamic programming, which is equivalent to the above bottom-up version but explores the search space incrementally from left to right; see Supplementary Fig. 4 for the pseudocode. This left-to-right order also enables beam search^44^, a classical pruning technique, to significantly narrow down the search space without sacrificing too much search quality. Our real system uses this left-to-right dynamic programming on the Turner nearest-neighbour free-energy model^15,16^, and our thermodynamic parameters follow LinearFold and Vienna RNAfold^45^, except for the dangling ends, which do not contribute stability in LinearDesign. Dangling ends refer to stabilizing interactions for multiloops and external loops^46^, which require knowledge of the nucleotide sequence outside of the state $\left({q}_{i},{q}_{j}\right)$. Though it could be integrated in LinearDesign, the implementation is more involved so we leave it to future work.

#### DFAs for other genetic codes, coding constraints and modified nucleotides

The DFA framework can also represent less common cases such as alternative genetic codes, modified nucleotides, and coding constraints. First, the DFA can encode non-standard genetic codes, such as alternative nuclear code for some yeast^47^ and mitochondrial codes^48^ (Extended Data Fig. 3a). Second, we may want to avoid some unwanted or rare codons (such as the amber stop codon) which is an easy change on the codon DFAs (Extended Data Fig. 3b), or certain adjacent codon pairs that modulate translation efficiency^49^, which is beyond the scope of single codon DFAs but easy on the mRNA DFA (Extended Data Fig. 3c). Similarly, we may want to disallow certain restriction enzyme recognition sites, which span across multiple codons (Supplementary Fig. 5). Finally, chemically modified nucleotides such as pseudouridine (Ψ) have been widely used in mRNA vaccines^38^, which can also be incorporated in the DFA (Extended Data Fig. 3d).

#### Related work

Here we first discuss the advantages of our algorithm over previous work, and then discuss a recent work^29^ that uses LinearDesign in experimental screening.

Two previous studies^22,23^ also tackled the problem of optimal-stability mRNA design (that is, our objective 1) via dynamic programming, but their algorithms are complicated, not generalizable and less efficient. By contrast, the stability-only version of our work reduced the mRNA design problem to the classical computational linguistics problem of lattice parsing, resulting in a much simpler and more efficient algorithm that is vastly different from the specifically-designed algorithms such as the one described in Cohen et al.^22^ and CDSfold^23^. More importantly, our work further solves the harder and practically more important problem of joint optimization between stability and codon optimality, which subsumes the stability-only objective as a special case. Here we comprehensively compare our work to the previous ones in the following seven aspects.

##### Lattice representation of the design space

Our work is the first to use automata theory to compactly and conveniently represent the exponentially large mRNA design space. By contrast, Cohen et al. and CDSfold extend the standard Zuker algorithm with the consideration of amino acid constraints, and they do not have any graph-theoretic or formal representation of the design space. To handle the nucleotide dependencies of the first and third positions in the codons of leucine and arginine, CDSfold introduces the ‘extended nucleotides’, which classify the same nucleotide at the second position with different notations regarding the dependency. See Supplementary Fig. 6 for the lattice representation of leucine in our work as an example, and the extended nucleotides of leucine in CDSfold as a comparison. More importantly, our lattice representation is able to integrate (the logarithm) of CAI for a joint optimization of stability and codon optimality, and is general for arbitrary genetic code; see the details in later paragraphs.

##### Lattice parsing

Based on our DFA representation, we further reduce the mRNA design problem to the classical computational linguistics problem of lattice parsing, which aims to find the most grammatical sentence among exponentially many alternatives. This problem was solved by Bar-Hillel et al. in 1961 (ref. ^19^). Therefore, instead of inventing a new algorithm, we simply adapt the classical lattice parsing to mRNA design using our algorithm of LinearDesign. Note that the single-sequence folding is a special case of our algorithm where the lattice is a single chain.

##### Efficiency

More interestingly, our simple adapted algorithm reduces the constant factor of the cubic-time bifurcation rule that dominates the runtime of mRNA design, leading to better efficiency over previous work such as CDSfold. Supplementary Fig. 7b illustrates the space and time complexity under the classical Nussinov energy model.

The single-sequence RNA folding defines a span $\left(i,j\right)$ as an item, where $i$ and $j$ are indices in the RNA sequence. For a sequence with *n* nucleotides, during dynamic programming, at most ${n}^{3}$ items are generated for the bifurcation rule $S\to {S\; P}$; space-wise, at most ${n}^{2}$ items are stored.

Extending RNA folding to lattice parsing, our work defines each item as $\left({q}_{i},{q}_{j}\right)$, where ${q}_{i}$ and ${q}_{j}$ are the nodes in the lattice: ${q}_{i}\in \{\left(i,0\right),\left(i,1\right)\}$ and${q}_{j}\in \{\left(\,j,0\right),\left(\,j,1\right)\}$. Since there are at most two nodes at each position, the number of items stored is at most $4{n}^{2}$. For the bifurcation rule $S\to S\,P$, items $\left({q}_{i},{q}_{k}\right)$ and $\left({q}_{k},{q}_{j}\right)$ are combined to form a bigger item $\left({q}_{i},{q}_{j}\right)$, in which at most $8{n}^{3}$ items are generated (at most two nodes each for $i$, $k$ and $j$). See Supplementary Fig. 7c for the illustration of above analysis; see lines 22–25 in Supplementary Fig. 2 and lines 20–24 in Supplementary Fig. 4 for the pseudocode of the bifurcation case in our work.

By contrast, CDSfold defines each item as $\left(i,j,{{\rm{nuc}}}_{i},{{\rm{nuc}}}_{j}\right)$, where ${{\rm{nuc}}}_{i}$ and ${{\rm{nuc}}}_{j}$ are the nucleotides at positions $i$ and $j$, respectively. The number of items stored in CDSfold scales $16{n}^{2}$, because there are at most 4 nucleotide types for each ${{\rm{nuc}}}_{i}$ and ${{\rm{nuc}}}_{j}$. For the bifurcation rule $S\to S\,P$, items $\left(i,k,{{\rm{nuc}}}_{i},{{\rm{nuc}}}_{k}\right)$, and $\left(k+1,j,{{\rm{nuc}}}_{k+1},{{\rm{nuc}}}_{j}\right)$, are combined to form $\left(i,j,{{\rm{nuc}}}_{i},{{\rm{nuc}}}_{j}\right)$, in which at most $128{n}^{3}$ items are generated (at most $4\times 4$ nucleotide types at ${{\rm{nuc}}}_{i}$ and ${{\rm{nuc}}}_{j}$, and $4\times 2$ nucleotide pairs between ${{\rm{nuc}}}_{k}$ and ${{\rm{nuc}}}_{k+1}$). See Supplementary Fig. 7d for the analysis illustration of CDSfold.

Compared to CDSfold, our work largely reduces the time complexity constant of the bifurcation rule $S\to S\,P$ from 128 to 8. The cubic-time bifurcation rule which dominates the runtime in CDSfold is greatly accelerated in our algorithm. Empirically, our algorithm scales quadratically rather than cubically with mRNA sequence length for practical applications (Fig. 3 and Supplementary Fig. 8).

##### Joint optimization of stability and codon optimality

Codon optimality is an important factor in mRNA design, which should be jointly optimized with stability^5^, and our work is the first to solve this joint optimization problem, thanks to the DFA representation and lattice parsing. By contrast, previous work (Cohen et al. and CDSfold) does not perform, and is impossible to be extended to perform, such a joint optimization. First, Cohen et al. only optimize stability without considering codon optimality. CDSfold uses simulated annealing to improve CAI by fine-tuning from the MFE solution, but this is a heuristic with no guarantees. Second, CDSfold’s objective function, ${\rm{MFE}}\,\bullet \,{{\rm{CAI}}}^{\lambda }$, is impossible for dynamic programming due to the difference between MFE and CAI, where MFE is a sum of free energy for each component substructure (additive) but CAI is a geometric mean of the relative codon usages (multiplicative). To reconcile this difference, our formulation defines a novel objective that factors the logarithm of CAI for an mRNA additively onto its individual codons, thus making it decomposable and amenable to dynamic programming (see ‘The LinearDesign algorithm’ for details). By contrast, CDSfold’s objective formulation does not factor into individual codons, and thus cannot be incorporated into global optimization. Last but most importantly, even if CDSfold were to borrow our formulation, its fundamental codon representation still rules out joint optimization. Our framework easily encodes (the logarithm of) CAI in our DFA representation, for example, we can integrate CAI onto a weighted DFA for leucine (Supplementary Fig. 6a). By contrast, CDSfold has to use an extended nucleotides representation for codon choices, which makes it impossible to do joint optimization with CAI (Supplementary Fig. 6b,c).

To summarize, our framework easily incorporates codon optimality into the joint optimization that previous work did not (and could not be extended to) tackle. Our objective integrates (the logarithm of) CAI and MFE together, while the objective of CDSfold is not able to reconcile these two factors. Furthermore, even if using our objective formulation, CDSfold’s representation of codon choices still rules out the possibility of CAI integration.

##### Generalizability

Our DFA framework is so general that it can also represent arbitrary (non-standard) genetic codes, modified nucleotides, and coding constraints such as adjacent codon pair preference, which previous work could not handle even with major modifications. See ‘DFAs for other genetic codes, coding constraints and modified nucleotides’ for details.

##### Linear-time version for long sequence and suboptimal candidates

We further develop a faster, linear-time, approximate version which greatly reduces runtime for long sequences with small sacrifices in search quality, which we also use to generate multiple suboptimal candidates with varying folding stability and codon optimality as candidates for experimentation.

##### Verification of wet laboratory experiments

Extensive experiments confirm that compared to the standard codon-optimization benchmark, our designs are substantially better in chemical stability and protein expression in vitro, and the corresponding mRNA vaccines elicit up to 128 times higher antibody responses in vivo.

Another recent work^29^ optimized mRNA designs and screened them via an experimental platform. LinearDesign had a central role in their work as the starting point of their optimizations (see figure 4b of their paper), followed by fine-tunings by both human players and a Monte Carlo tree search algorithm. The resulting coding regions are flanked by different UTRs, and then tested on stability and protein expression. LinearDesign-generated sequences showed strong stability and protein expression results with different UTRs (figures 2g and 4a of their paper), independently confirming our in vitro experiments. However, they did not perform any in vivo validations.

#### Benchmark dataset and machine

To estimate the time complexity of LinearDesign, we collected 114 human protein sequences from UniProt^24^, with lengths from 78 to 3,333 amino acids (not including the stop codon); see Supplementary Table 1. We benchmarked LinearDesign on a Linux machine with 2 Intel Xeon E5-2660 v3 CPUs (2.60 GHz) and 377 GB memory, and used Clang (11.0.0) to compile. The code only uses a single thread.

#### Additional design constraints

Some studies have shown that protein expression level drops if the 5′-end leader region has more secondary structure^5,50–53^. To design sequences with less structures at 5′-end leader region, we take a simple ‘design, enumerate and concatenate’ strategy to avoid structure in the leader region: (1) design the CDS region except for the 5′-end leader region (that is, the first 15 nucleotides); (2) enumerate all possible subsequences in the 5′-end leader region; and (3) concatenate each subsequence with the designed sequence, refold, and choose the one whose 5′-end leader region has the most unpaired nucleotides.

In addition, it has been revealed that long double-stranded regions may induce unwanted innate immune responses by previous studies^27,54,55^. Considering this, we do not allow long double-stranded regions that include 33 or more base pairs by adding this constraint in the design process.

#### RNA secondary structure prediction and visualization

Vienna RNAfold from ViennaRNA package (version 2.4.14) is used for predicting and drawing the secondary structure of mRNA sequence, and calculating the MFE of secondary structures.

### In vitro and in vivo experiments

#### Preparation of mRNA and its formulation

mRNA molecules were synthesized in vitro by T7 RNA polymerase using linearized plasmid as DNA template. The open reading frame region is flanked with the 5′ and 3′ UTRs followed by a 70-nt poly-A tail. For all spike protein-coding sequences, the in vitro transcription reaction was conducted at 37 °C for 4 h, followed by digestion with DNase I (Hongene Biotech). mRNA encoding full-length spike protein without proline substitution was then capped using Vaccinia Capping Enzyme (Hongene Biotech) and purified with magnetic Dynabeads (Thermo Fisher). Eluted mRNA was further treated with Antarctic Phosphatase (Hongene Biotech) at 37 °C for 30 min to remove residual 5′-triphosphates. For all VZV gE sequences, mRNA was co-transcriptionally capped using m7(3′OMeG)(5′)ppp(5′)(2′OMeA)pG capping reagent (Hongene Biotech) in a ‘one-pot’ reaction at 30 °C for 16 h, followed by treatment with DNase I. Capped mRNA encoding spike or VZV gE protein was then purified using beads. For the preparation of formulated mRNA vaccines, lipopolyplex (LPP) formulation was used to encapsulate mRNA cargo as described previously^56^. LPP is a lipid-based mRNA delivery system and has been demonstrated to provide high efficacy and good safety profile^26^.

#### Agarose gel electrophoresis and integrity assay of mRNA

To study the electrophoretic mobility profile of mRNA molecules, mRNA samples suspended in Ambion RNA storage buffer (Thermo Fisher) were denatured at 75 °C for 5 min and snap-cooled on ice before being loaded onto 1% non-denaturing agarose gel (130 V for 1 h at room temperature). Gel image was taken by Gel Doc XR+ Gel Documentation System (Bio-Rad).

To assess the in-solution stability of mRNA, samples were incubated in PBS buffer containing 10 mM Mg^2+^. Sampling was conducted at time points (0, 1, 2, 4, 8, 12, 16, 24, 32, 48 and 60 h). For a faster degradation process, PBS buffer containing 20 mM Mg^2+^ instead of 10 mM was used. Sampling was done in a relatively shorter time span (0, 1, 2, 4, 8, 12, 15, 18, 21, and 24 h). RNA integrity was analysed by Qsep100 Capillary Electrophoresis System. The integrity was represented as the proportion of full-length mRNA calculated on electropherogram. The data were normalized to time point 0 h. To extrapolate the half-life of each sequence, one-phase decay equation:

$$Y=\left({Y}_{0}-{\rm{plateau}}\right)\cdot {{\rm{e}}}^{-KX}+{\rm{plateau}}$$

was used to fit the curve. The ${Y}_{0}$ and plateau were set as 100 and 0, respectively. Half-life was computed as $\mathrm{ln}\left(2\right)/K$, where $K$ refers to decay rate constant.

#### Protein expression assay

HEK293 cells (ATCC) were cultured in Dulbecco’s Modified Eagle’s Medium (DMEM) (Hyclone) containing 10% fetal bovine serum (FBS) (GEMINI) and 1% penicillin-streptomycin (Gibco). All cells were cultured at 37 °C in a 5% CO_2_ condition.

For the measurement of protein expression, cells were transfected with mRNA using Lipofectamine MessengerMAX (Thermo Scientific). In brief, a mix of 2 µg mRNA and 6 µl of Lipofectamine reagent was prepared following the manual instructions and then incubated with cells for 24 or 48 h. For flow cytometric analysis, cells were collected and stained with Live/Dead cell dye (Fixable Viability Stain 510, BD) for 5 min. After washing, cells were incubated with anti-spike receptor-binding domain (RBD) chimeric monoclonal antibody (1:100 dilution, Sino Biological) for 30 min, followed by washing and incubation with PE-anti-human IgG Fc (1:100 dilution, Biolegend) for 30 min. Samples were analysed on BD Canto II (BD Biosciences). Data were processed using FlowJo V10.1 (Tree Star).

#### In vivo immunogenicity study

C57BL/6 mice (6 to 8 weeks of age) were intramuscularly immunized twice with 10 µg LPP-formulated mRNA vaccines at a 2-week interval. Sera and spleens were collected 14 days after boost shot.

##### Surrogate virus neutralization assay

Neutralizing antibody titre was measured using surrogate virus neutralization assay as previously described^57^, with some modifications. In brief, 96-well plates (Greiner Bio-one) were coated with recombinant with human ACE2 protein (100 ng per well, Genscript) overnight at 4 °C. Plates were washed with 1 × PBS-T and blocked with 2% BSA for 2 h at room temperature. HRP-conjugated RBD (100 ng ml^−1^) was incubated with serially diluted serum from immunized mice at an equal volume (60 µl each) for 30 min at 37 °C. Sera collected from PBS-treated mice were used as negative control. Then a 100 µl mixture of RBD and serum was added into each well and incubated for 15 min at 37 °C. After washing, TMB substrate (Invitrogen) was used for colour development and the absorbance at 450 nm was recorded using BioTek microplate reader. The IC_50_ value was calculated using four-parameter logistic non-linear regression.

##### Enzyme-linked immunosorbent assays

In brief, recombinant SARS- CoV-2 spike ectodomain protein or VZV gE protein (Genscript) diluted in coating buffer (Biolegend) were used to coat 96-well EIA/RIA plates (Greiner Bio-one, 100 ng per well) at 4 °C overnight. The plates were then washed with 1$\times$PBS-T (0.05% Tween-20) and blocked with 2% BSA in PBS-T for 2 h at room temperature. Serum samples with serial dilutions were added and incubated for 2 h at room temperature. After washing, HRP-conjugated goat anti-mouse IgG antibody (1:10,000) was added and incubated for 1 h. TMB substrate (Invitrogen) was then used for colour development and the absorbance was read at 450 nm using BioTek microplate reader. End-point titres were calculated as the largest sample dilution factor yielding a signal that exceeds 2.1-fold value of the background^58^.

##### ELISpot assay

Frequency of spike (or VZV gE) antigen-specific IFNγ-secreting T cells was evaluated using Mouse IFNγ ELISpotplus Kit (Mabtech) according to the manual. In brief, 3 × 10^5^ mouse splenocytes were added to wells pre-coated with anti-mouse IFNγ capturing antibodies and were incubated with spike protein or VZV gE peptide pool (10 µg ml^−1^) for 20 h. After washing, plates were incubated with Streptavidin–alkaline phosphatase (1:1,000) for 1 h at room temperature. Spots were developed with BCIP/NBT substrate solution and counted using Immunospot S6 analyzer (CTL). Due to multiple steps and exponential change of antibody and antigen-specific T cells during the immunity induction process, in vivo immunogenicity data usually have high data variations. Inoculation of mRNA vaccine involves extra processes such as tissue transfection and protein translation, and the variations in these process efficiencies together with variable dosing and differences in individual mouse’s immune status usually bring more immunogenicity variations than protein-based vaccines. From our experience, the variations observed in this study are typical for mRNA vaccines.

#### Ethics statement

All mouse studies were performed in strict accordance with the guidelines set by the Chinese Regulations of Laboratory Animals and Laboratory Animal-Requirements of Environment and Housing Facilities. Animal experiments were carried out with the approval from the Institutional Animal Care and Use Committee (IACUC) of Shanghai Model Organisms Center.

#### Statistics and reproducibility

Geometric means or arithmetic means are represented by the heights of bars, or symbols, and error bars represent the corresponding s.d. Two-tailed Mann–Whitney U tests were used to compare two experimental groups for the in vivo studies. To compare more than two experimental groups, one-way ANOVA with Dunn’s multiple comparisons tests were applied in the in vitro protein expression experiment. Statistical analyses were performed using Prism v.8 (GraphPad). *P < 0.05, **P < 0.01, ***P < 0.001. The raw *P* values from the statistical analysis are summarized in the figshare file (10.6084/m9.figshare.22193251). In vitro experiments were independently repeated in triplicate. Animal experiments were completed once. Gel electrophoresis experiment was repeated three times to obtain similar results.

#### Data reporting

No statistical methods were used to predetermine sample size. The experiments were not randomized. The investigators were not blinded to allocation during experiments and outcome assessment.

### Reporting summary

Further information on research design is available in the Nature Portfolio Reporting Summary linked to this article.

## Online content

Any methods, additional references, Nature Portfolio reporting summaries, source data, extended data, supplementary information, acknowledgements, peer review information; details of author contributions and competing interests; and statements of data and code availability are available at 10.1038/s41586-023-06127-z.

### Supplementary information

Supplementary Information This file contains Supplementary Figs. 1–12, Supplementary Tables 1–3, and sequence information.

Reporting Summary

### Source data

Source Data Fig. 4

Source Data Fig. 5

Source Data Extended Data Fig. 7

## Figures and Tables

**Extended Data Table 1:**
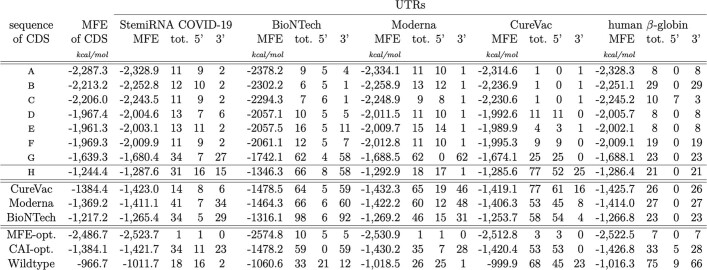
The LinearDesign-generated coding-region sequences, due to more secondary structures within the coding region, are less likely to form base pairs with or interfere with the structures of the UTRs

Here we show the numbers of predicted base pairs between UTRs and the coding regions of SARS-CoV-2 Spike protein. We used 5 different UTRs: StemiRNA COVID-19 UTRs used in wet lab experiments, BNT-162b2 (BioNTech) UTRs, mRNA-1273 (Moderna) UTRs, CV2CoV (CureVac) UTRs, and a widely used human *β*-globin mRNA UTRs. We tested 14 different sequences of the coding region: sequences A–H for wet lab experiments, sequences from three main mRNA vaccine companies, MFE-opt. and CAI-opt. sequences (i.e., sequences with the lowest folding free energy and with CAI = 1, respectively), and the wildtype sequence. Most of the LinearDesign-generated mRNA sequences (sequences A–F and MFE-opt.) form fewer base pairs with UTRs. The folding free energies and structures are predicted by Vienna RNAfold (-d0 mode); MFEs of CDS are calculated without stop codon.

**Extended Data Table 2:**
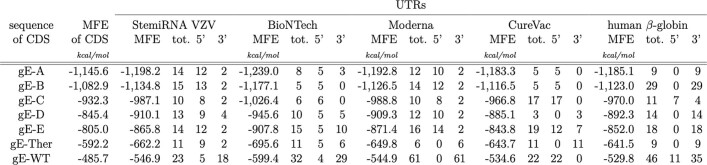
Similar to Extended Data Table 1, LinearDesign-generated coding-regions for the VZV gE protein form less base pairs with the UTRs

Here we used 5 different UTRs: StemiRNA VZV UTRs used in wet lab experiments, BNT-162b2 (BioNTech) UTRs, mRNA-1273 (Moderna) UTRs, CV2CoV (CureVac) UTRs, and human *β*-globin mRNA UTRs. The 7 coding sequences are gE A–E, gE-Ther and gE-WT, which are used in the wet lab experiments of VZV. The folding free energies and structures are predicted by Vienna RNAfold (-d0 mode); MFEs of CDS are calculated without stop codon.

## Data Availability

The UniProt sequences used to estimate the time complexity of LinearDesign are included in Supplementary Table 1 and are deposited at our figshare repository (10.6084/m9.figshare.22193251). The SARS-CoV-2 spike and VZV gE protein-coding sequences and UTR sequences used in the biological experiments are included at the end of the Supplementary Information file and are available at our figshare repository.  Source data are provided with this paper.

## Code availability

The LinearDesign source code is available to all parties on GitHub (https://github.com/LinearDesignSoftware/LinearDesign) and Zenodo (10.5281/zenodo.7839739), and is free for academic and research use.
