# Supplementary material for: Algorithm for optimized mRNA design improves stability and immunogenicity
Source: Nature. 2023 May 2;621(7978):396–403. doi: 10.1038/s41586-023-06127-z (PMC10499610; doi:10.1038/s41586-023-06127-z)
Supplement: Supplementary file 1 — This file contains Supplementary Figs. 1–12, Supplementary Tables 1–3, and sequence information. [file 41586_2023_6127_MOESM1_ESM.pdf]

nature portfolio

https://doi.org/10.1038/s41586-023-06127-z

Supplementary information

Algorithm for optimized mRNA design improves stability and immunogenicity

In the format provided by the authors and unedited

Supplementary Information

Algorithm for Optimized mRNA Design Improves Stability and Immunogenicity

List of Supplementary Information

- Supplementary Figures
  - Supplementary Figure 1: uncropped non-denaturing agarose gel images
  - Supplementary Figure 2: pseudocode of a (simplified) bottom-up version of our mRNA Design algorithm
  - Supplementary Figure 3: pseudocode of supporting functions
  - Supplementary Figure 4: pseudocode of (simplified) LinearDesign algorithm
  - Supplementary Figure 5: examples of DFA representations for coding constraints
  - Supplementary Figure 6: illustration of the difference in codon representation between LinearDesign and CDSfold
  - Supplementary Figure 7: illustration of the difference in computational complexity between LinearDesign and CDSfold
  - Supplementary Figure 8: additional time complexity analysis
  - Supplementary Figure 9: search error of LinearDesign's beam search mode
  - Supplementary Figure 10: secondary structures of sequences used in the COVID mRNA vaccines experiments
  - Supplementary Figure 11: secondary structures of sequences used in the VZV mRNA vaccines experiments
  - Supplementary Figure 12: protein expression of Spike mRNAs in HEK293 cells
- Supplementary Tables
  - Supplementary Table 1: UniProt sequences used for runtime analysis
  - Supplementary Table 2: statistics of sequences used in the COVID mRNA vaccine experiments
  - Supplementary Table 3: statistics of sequences used in the VZV mRNA vaccine experiments
- Supplementary Sequence Information
  - Sequences used in the COVID mRNA vaccine experiments, including coding region sequences A–H and BNT, and 5' and 3' UTR sequences
  - Sequences used in the VZV mRNA vaccine experiments, including coding region sequences gE A–E, gE-Ther and gE-WT, and 5' and 3' UTR sequences

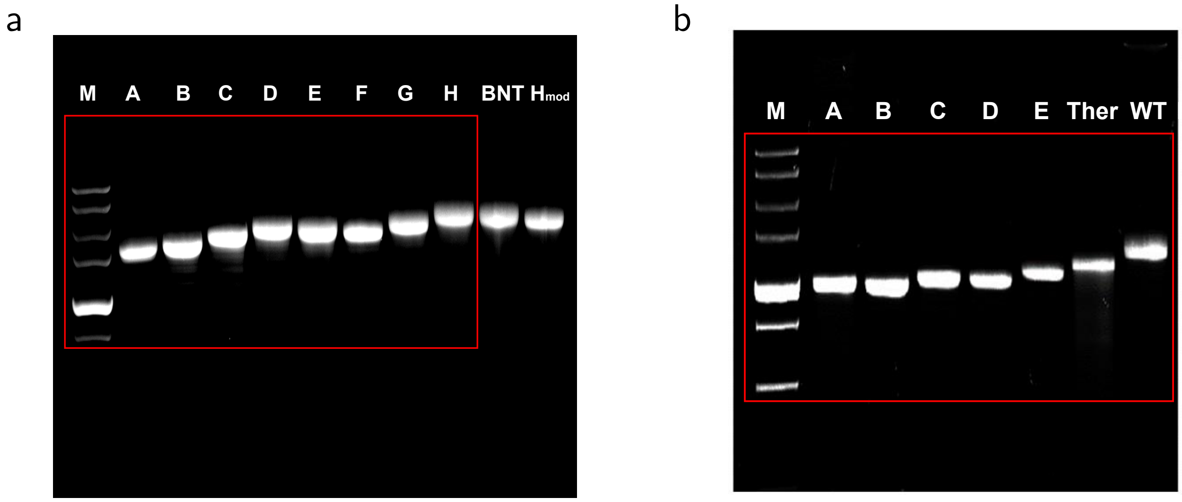

Supplementary Figure 1: Uncropped non-denaturing agarose gel images. **a**, Gel image of the Spike mRNAs. H_{mod} represents the chemical modified version of H. **b**, Gel image of the VZV gE mRNAs. Red frames indicate the cropped sections shown in Figs. 4b and 5b.

function BottomUpDesign($\mathbf{p}$, λ) ▷ $\mathbf{p}$: protein sequence; λ: weight of CAI
  $n \leftarrow 3 \cdot (|\mathbf{p}| + 1)$ ▷ mRNA length; +1 for the stop codon
  $D \leftarrow D(x_1) \circ D(x_2) \circ \ldots \circ D(\text{stop})$ ▷ build (CAI-integrated) mRNA DFA
  $\mathit{best} \leftarrow \text{hash}()$ ▷ hash table: from $[X, q_i, q_j]$ to score
  $\mathit{back} \leftarrow \text{hash}()$ ▷ hash table: from $[X, q_i, q_j]$ to backpointer
  for $i = 0 \ldots (n-3)$ **do** ▷ base case: $S \overset{0}{\rightarrow} N\ N\ N$
    for each $q_i \in \text{nodes}(D, i)$ **do**
      for each $q_{i+3} \in \text{nodes}(D, i+3)$ **do**
        $\mathit{best}[S, q_i, q_{i+3}] \leftarrow \text{mincost}(q_i, q_{i+3}, \lambda)$ ▷ best cost of any $q_i \rightsquigarrow q_{i+3}$ path (Eq. 7)
  for $l = 2 \ldots n$ **do** ▷ $l = (j - i)$ is the span width
    for $i = 0 \ldots (n - l)$ **do**
      $j \leftarrow i + l$
      for each $q_i \in \text{nodes}(D, i)$ **do**
        for each $q_j \in \text{nodes}(D, j)$ **do**
          for each $q_{j-1} \xrightarrow{b:w_b} q_j \in \mathit{in\_edges}(D, q_j)$ **do** ▷ $q_i \overset{S}{\rightsquigarrow} q_{j-1} \overset{b}{\rightsquigarrow} q_j$
            Update($S, q_i, q_j, \mathit{best}[S, q_i, q_{j-1}] + \lambda w_b, (q_{j-1}, b)$) ▷ $S \overset{0}{\rightarrow} S\ N$
            if $j - i > 4$ **then** ▷ pairing (no sharp turn)
              for each $q_i \xrightarrow{a:w_a} q_{i+1} \in \mathit{out\_edges}(D, q_i)$ **do** ▷ $q_i \overset{a}{\rightarrow} q_{i+1} \overset{S}{\rightsquigarrow} q_{j-1} \overset{b}{\rightarrow} q_j$
                if $\Delta G(a, b) < 0$ **then** ▷ $\Delta G(\text{C},\text{G}) = -3; \Delta G(\text{A},\text{U}) = -2; \ldots$
                  $\mathit{score} \leftarrow \mathit{best}[S, q_{i+1}, q_{j-1}] + \lambda(w_a + w_b) + \Delta G(a, b)$ ▷ $P \overset{-3}{\rightarrow} \text{C}\ S\ \text{G} \mid \ldots$
                  Update($P, q_i, q_j, \mathit{score}, (a, q_{i+1}, q_{j-1}, b)$)
          for $k = (i+1) \ldots (j-1)$ **do** ▷ bifurcation midpoint
            for each $q_k \in \text{nodes}(D, k)$ **do** ▷ $q_i \overset{S}{\rightsquigarrow} q_k \overset{P}{\rightsquigarrow} q_j$
              $\mathit{score} \leftarrow \mathit{best}[S, q_i, q_k] + \mathit{best}[P, q_k, q_j]$ ▷ $S \overset{0}{\rightarrow} S\ P$
              Update($S, q_i, q_j, \mathit{score}, q_k$)
  return $\mathit{best}[S, q_0, q_n]$, Backtrace($S, q_0, q_n$)

Supplementary Figure 2: The pseudocode of a simplified bottom-up version of our mRNA Design algorithm for the joint optimization between stability and codon optimality. The costs in purple are for CAI integration. See Methods §1.5 for more algorithm description, and Supplementary Figure 3 for Update and Backtrace functions.

function UPDATE($X, q_i, q_j, \mathit{score}, \mathit{backpointer}$)
  if key (X, q_i, q_j) not in *best* or $\mathit{score} < \mathit{best}[X, q_i, q_j]$ **then** ▷ minimizing weight
    $\mathit{best}[X, q_i, q_j] \leftarrow \mathit{score}$
    $\mathit{back}[X, q_i, q_j] \leftarrow \mathit{backpointer}$

function BACKTRACE(X, q_i, q_j) ▷ returns a (sequence, structure) pair
  $\mathit{backpointer} \leftarrow \mathit{back}[X, q_i, q_j]$
  if $\mathrm{index}(q_j) - \mathrm{index}(q_i) = 3$ **then** ▷ $\mathrm{index}(q)$: the string index of state q
    return ANYPATH(q_i, q_j), "..." ▷ $S \overset{0}{\to} N\ N\ N$; any $q_i \rightsquigarrow q_j$ path is fine
  if $\mathrm{length}(\mathit{backpointer}) = 4$ **then** ▷ pairing: $P \overset{-3}{\to} \text{C}\ S\ \text{G} \mid \ldots$
    $a, q_{i+1}, q_{j-1}, b \leftarrow \mathit{backpointer}$ ▷ $q_i \overset{a}{\to} q_{i+1} \overset{seq}{\rightsquigarrow} q_{j-1} \overset{b}{\to} q_j$
    $\mathit{seq}, \mathit{struct} \leftarrow$ BACKTRACE(S, q_{i+1}, q_{j-1})
    return $a + \mathit{seq} + b$, "(" $+ \mathit{struct} +$ ")"
  else if $\mathrm{length}(\mathit{backpointer}) = 2$ **then** ▷ skip: $S \overset{0}{\to} S\ N$
    $q_{j-1}, b \leftarrow \mathit{backpointer}$ ▷ $q_i \overset{seq}{\rightsquigarrow} q_{j-1} \overset{b}{\to} q_j$
    $\mathit{seq}, \mathit{struct} \leftarrow$ BACKTRACE(S, q_i, q_{j-1})
    return $\mathit{seq} + b, \mathit{struct} +$ "."
  else ▷ bifurcation: $S \overset{0}{\to} S\ P$
    $q_k \leftarrow \mathit{backpointer}$ ▷ $q_i \overset{seq_1}{\rightsquigarrow} q_k \overset{seq_2}{\rightsquigarrow} q_j$
    $\mathit{seq}_1, \mathit{struct}_1 \leftarrow$ BACKTRACE(S, q_i, q_k)
    $\mathit{seq}_2, \mathit{struct}_2 \leftarrow$ BACKTRACE(P, q_k, q_j)
    return $\mathit{seq}_1 + \mathit{seq}_2, \mathit{struct}_1 + \mathit{struct}_2$

function BEAMPRUNE(X, j, b)
  $\mathit{cands} \leftarrow \mathrm{hash}()$ ▷ hash table: from q_i to score $\mathit{best}[S, q_0, q_i] + \mathit{best}[X, q_i, q_j]$
  for each $q_j \in \mathit{nodes}(j)$ **do**
    for each key $(X, q_i, q_j) \in \mathit{best}$ **do**
      $\mathit{cands}[q_i] \leftarrow \mathit{best}[S, q_0, q_i] + \mathit{best}[X, q_i, q_j]$ ▷ $\mathit{best}[S, q_0, q_i]$ as prefix score
  $\mathit{cands} \leftarrow$ SELECTTOPB(cands, b) ▷ select top-b by score
  for each key $(X, q_i, q_j) \in \mathit{best}$ **do**
    if key q_i not in *cands* **then**
      delete (X, q_i, q_j) in *best* ▷ prune out low-scoring states

Supplementary Figure 3: The pseudocode for UPDATE, BACKTRACE (used in BOTTOMUPDESIGN, see Supplementary Figure 2) and BEAMPRUNE (used in LINEARDESIGN, see Supplementary Figure 4) functions.

1: **function** LinearDesign($\mathbf{p}, \lambda, b$) ▷ $b = \infty$ means exact search

10: **for** $j = 4 \ldots n$ **do**

11: **for each** $q_{j-1} \in \text{nodes}(D, j-1)$ **do**

12: **for each** q_i such that $(S, q_i, q_{j-1}) \in best$ **do**

13: **for each** $q_{j-1} \xrightarrow{b:w_b} q_j \in out\_edges(D, q_{j-1})$ **do** ▷ $q_i \overset{S}{\rightsquigarrow} q_{j-1} \overset{b}{\rightarrow} q_j$

14: Update($S, q_i, q_j, best[q_i, q_{j-1}] + \lambda w_b, (q_{j-1}, b)$) ▷ $S \overset{0}{\rightarrow} S\ N$

15: **for each** $q_{i-1} \xrightarrow{a:w_a} q_i \in in\_edges(D, q_i)$ **do** ▷ $q_{i-1} \overset{a}{\rightarrow} q_i \overset{S}{\rightsquigarrow} q_{j-1} \overset{b}{\rightarrow} q_j$

16: **if** $\Delta G(a, b) < 0$ **then** ▷ $\Delta G(\text{C},\text{G}) = -3; \Delta G(\text{A},\text{U}) = -2; \ldots$

17: $score \leftarrow best[S, q_i, q_{j-1}] + \lambda(w_a + w_b) + \Delta G(a, b)$

18: Update($P, q_{i-1}, q_j, score, (a, q_i, q_{j-1}, b)$) ▷ $P \overset{-3}{\rightarrow} \text{C}\ S\ \text{G} \mid \ldots$

19: **if** $b \neq \infty$ **then** BeamPrune(P, j, b) ▷ choose top-b among all (P, q_i, q_j)'s

20: **for each** $q_j \in nodes(D, j)$ **do**

21: **for each** q_i such that $(P, q_i, q_j) \in best$ **do**

22: **for each** q_k such that $(S, q_k, q_i) \in best$ **do**

23: $score \leftarrow best[S, q_k, q_i] + best[P, q_i, q_j]$ ▷ $q_i \overset{S}{\rightsquigarrow} q_k \overset{P}{\rightsquigarrow} q_j$

24: Update($S, q_k, q_j, score, q_i$) ▷ $S \overset{0}{\rightarrow} S\ P$

25: **if** $b \neq \infty$ **then** BeamPrune(S, j, b) ▷ choose top-b among all (S, q_i, q_j)'s

26: **return** $best[S, q_0, q_n]$, Backtrace(S, q_0, q_n)

Supplementary Figure 4: The pseudocode of (simplified) LinearDesign algorithm for the joint optimization between stability and codon optimality. The costs in purple are for CAI integration. The first 9 lines are the same as in BottomUpDesign (see Supplementary Figure 2). See Methods §1.6 for more algorithm description, and Supplementary Figure 3 for Update, Backtrace, and BeamPrune functions.

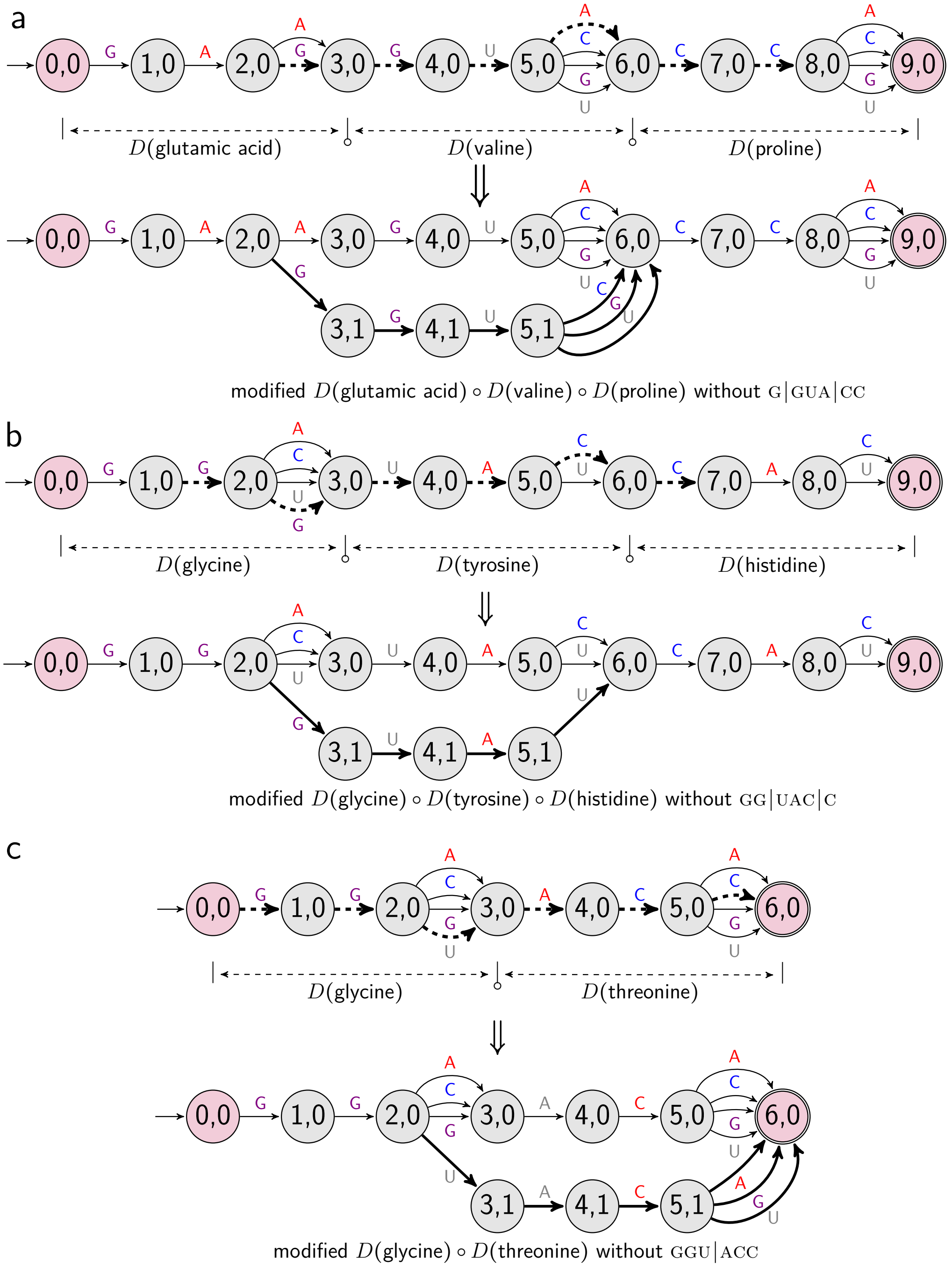

Supplementary Figure 5: Avoiding a restriction enzyme-specific recognition sequence (KpnI restriction enzyme recognization site: GGUACC). The enzyme recognization sequence is beyond one codon. **a–c** show three different partitions that split the sequence into different codons.

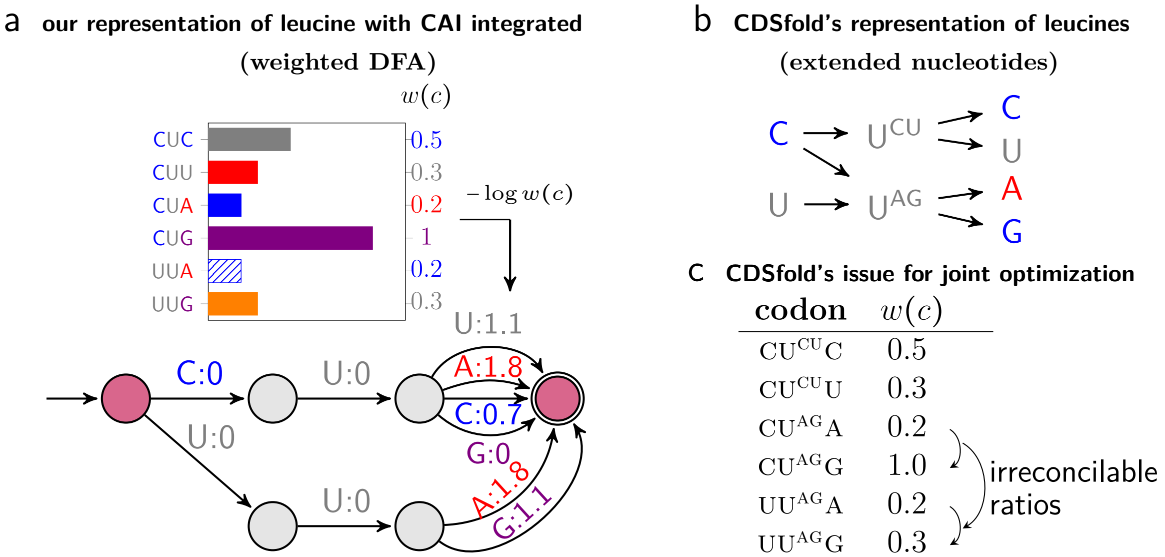

Supplementary Figure 6: In our DFA representation, it is easy to encode CAI; by contrast, this CAI integration is impossible or would require substantial revisions under CDSfold's representation. **a,** The weighted DFA of leucine, integrating each codon's (the logarithm of) CAI into edges. **b,** CDSfold's representation of leucine. **c,** The "extended nucleotides" representation of CDSfold is impossible to be extended to do such joint optimization, since the CAI is not integratable in their representation. For example, all six codons of leucine share the same second position (U), but it is classified into two different U's, denoted as U^{AG} and U^{CU}, to handle the dependencies between the first and the third positions in the six codons. In such a representation of leucine, the codons CU^{AG}A and UU^{AG}A share the same A at the last position, and the codons CU^{AG}G and UU^{AG}G share the same G at the last position. The codon CAIs (i.e., $w(c)$) of CU^{AG}A and CU^{AG}G are 0.2 and 1, respectively; while the codon CAIs of UU^{AG}A and UU^{AG}G are 0.2 and 0.3, respectively. The difference between the two CAI ratios (0.2:1 vs. 0.2:0.3) is irreconcilable, thus there is no way to reflect such a difference in this extended nucleotides representation. The case of arginine is similar. Therefore, even if CDSfold were to use our objective, it is still fundamentally impossible to be extended to do joint optimization.

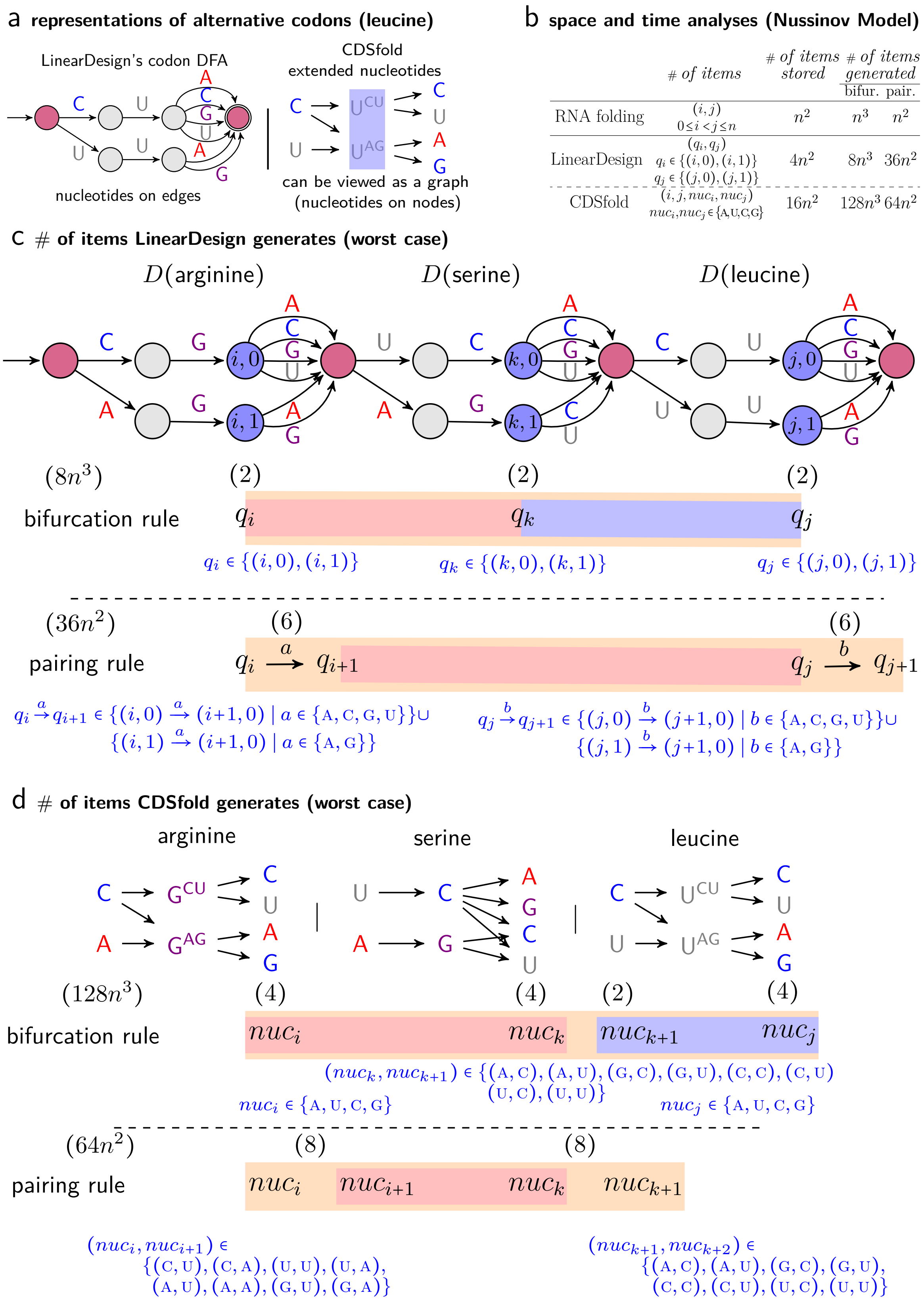

Supplementary Figure 7: Complexity comparison between CDSfold and LinearDesign. **a,** representation of leucine in LinearDesign and CDSfold. **b,** storage and time analyses. LinearDesign's cubic-time bifurcation rule is so efficient that it is dominated by the quadratic-time pairing rule in practice. See **c–d** for details. **c–d,** number of items generated analyses of bifurcation and pairing rules.

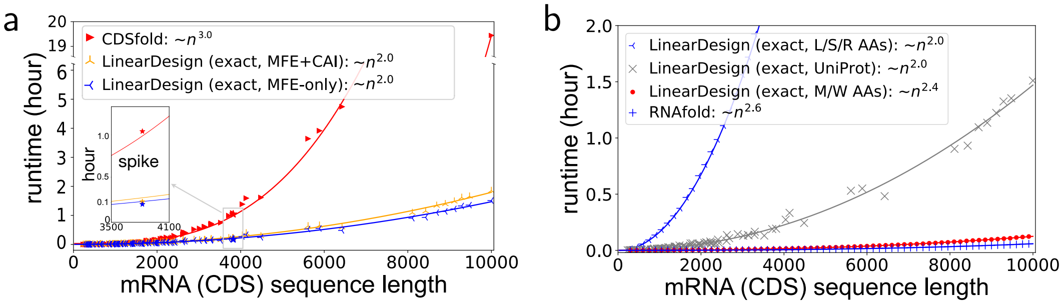

Supplementary Figure 8: Additional time complexity analysis. **a,** Runtime comparison between LinearDesign and CDSfold on UniProt proteins. Overall, LinearDesign is substantially faster than CDSfold, and more importantly, LinearDesign scales quadratically with sequence length in practice, while CDSfold runs in super-cubic time empirically. This difference can be explained by the analysis in **b**. On the other hand, our MFE+CAI mode (with $\lambda = 3$) is only slightly slower than our MFE-only version, while CDSfold cannot jointly optimize MFE and CAI. **b,** Runtime comparison of LinearDesign on proteins composed completely of amino acids with the most (i.e. 6) synonymous codons, natural (UniProt) proteins, and composed entirely of unambiguous (1-codon) amino acids, the last of which is equivalent to single sequence folding. We also ran RNAfold on RNA sequences that encode the unambiguous amino acids. See also Fig. 3 for more *in silico* results of LinearDesign, and Supplementary Table 1 for sequences used in runtime benchmarking.

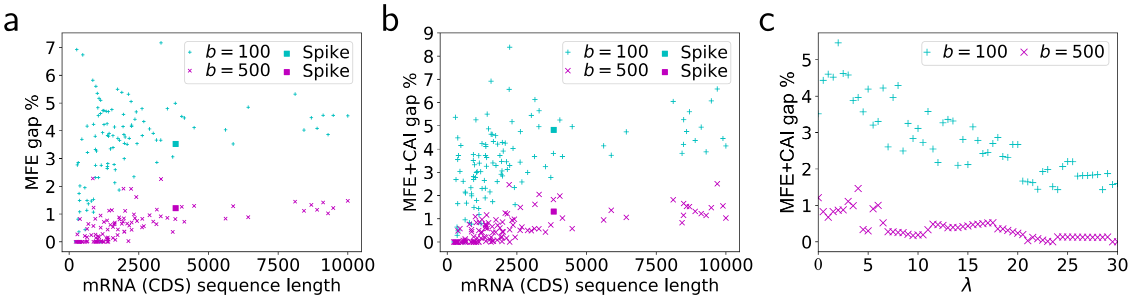

Supplementary Figure 9: Search error of LinearDesign's beam search mode against sequence length and λ (the weight of CAI in the joint optimization). **a–b,** Search error is relatively small, and does not deteriorate with sequence length. Here we used beam sizes 500 (purple) and 100 (cyan), on UniProt proteins (crosses) and SARS-CoV-2 Spike protein (squares), and we use $\lambda = 3$ for **b**. **c,** Search error decreases with λ. Note that the search error in **a** is the free energy gap % for $\lambda = 0$, defined as $1 - \mathrm{MFE}(\mathbf{r}_{\text{approx\_design}})/\mathrm{MFE}(\mathbf{r}_{\text{exact\_design}})$; the search error in **b–c** is defined as $1 - \mathrm{MFECAI}_\lambda(\mathbf{r}_{\text{approx\_design}})/\mathrm{MFECAI}_\lambda(\mathbf{r}_{\text{exact\_design}})$, where $\mathbf{r}_{\text{approx\_design}}$ and $\mathbf{r}_{\text{exact\_design}}$ are designed mRNAs from the beam search mode and the exact search mode, respectively. See also Fig. 3 for more *in silico* results, and Supplementary Table 1 for sequences used in these analyses.

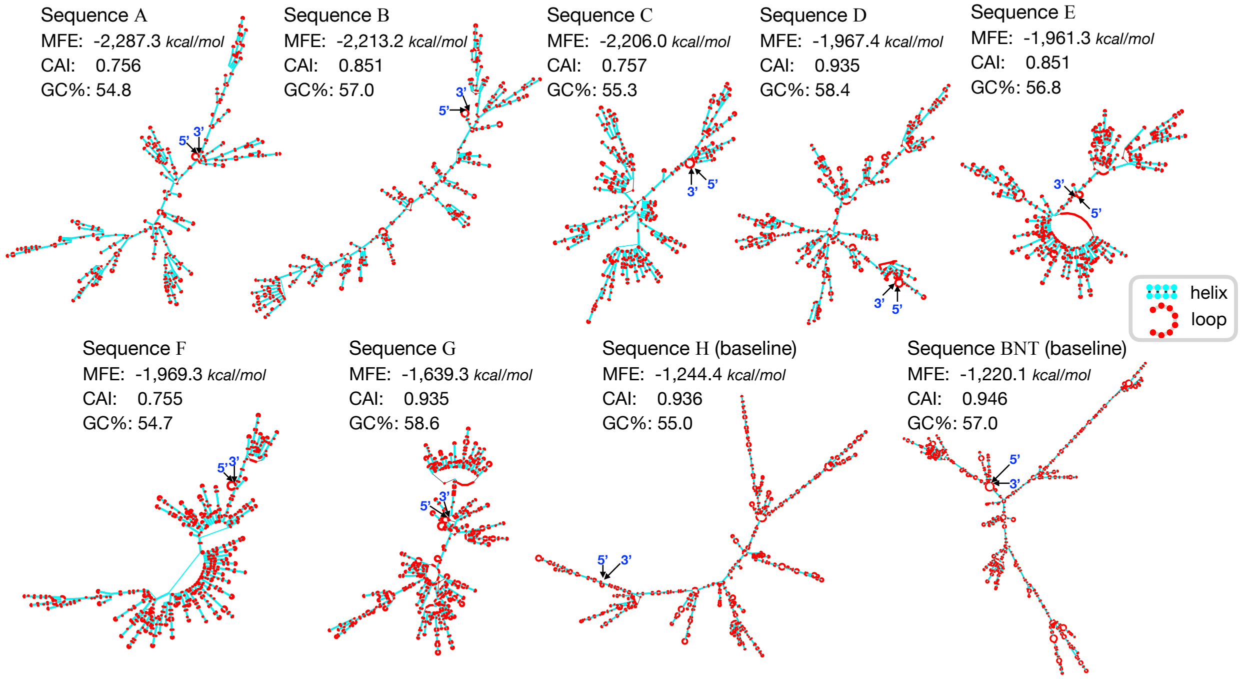

Supplementary Figure 10: The secondary structures of LinearDesign-generated sequences (A–G), and the baseline sequences (H and BNT) used in the COVID-19 wet lab experiments. The stable helices are in cyan and unstable loops are in red. Sequences A–D clearly have less and smaller loops, and they have higher levels of antibody responses compared to the baseline H (see Fig. 4e–g). The secondary structures are predicted by Vienna RNAfold (-d0 mode) and visualized by RNAplot.

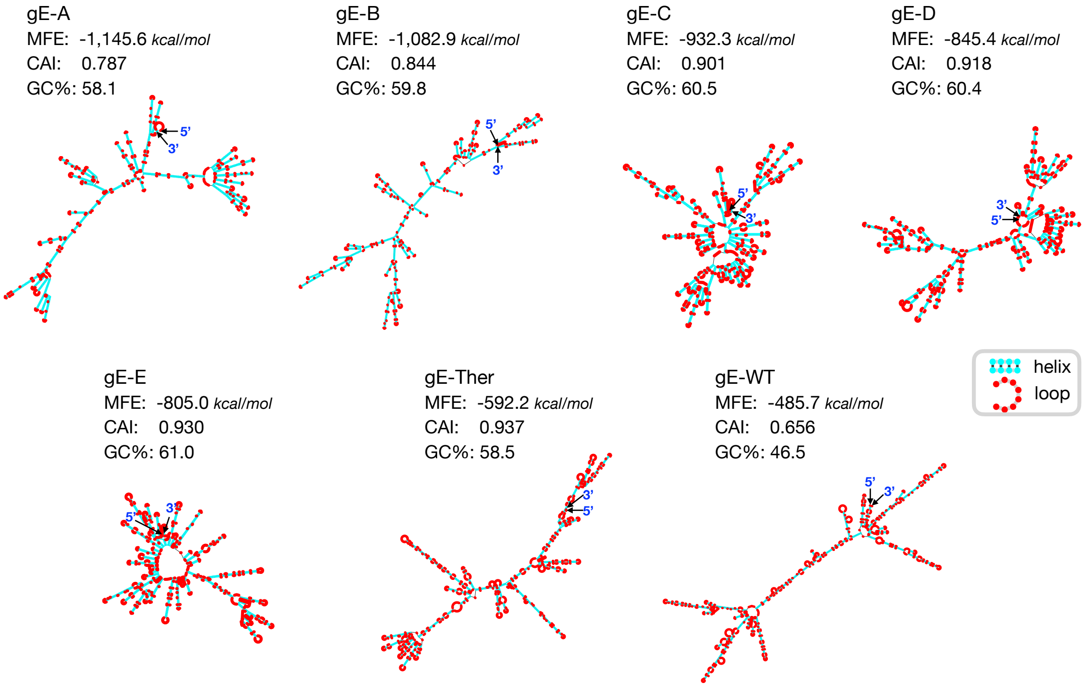

Supplementary Figure 11: The secondary structures of LinearDesign-generated sequences (gE A–E) and the baseline sequences (gE-Ther and WT) used in the VZV wet lab experiments. The stable helices are in cyan and unstable loops are in red. The secondary structures are predicted by Vienna RNAfold (-d0 mode) and visualized by RNAplot.

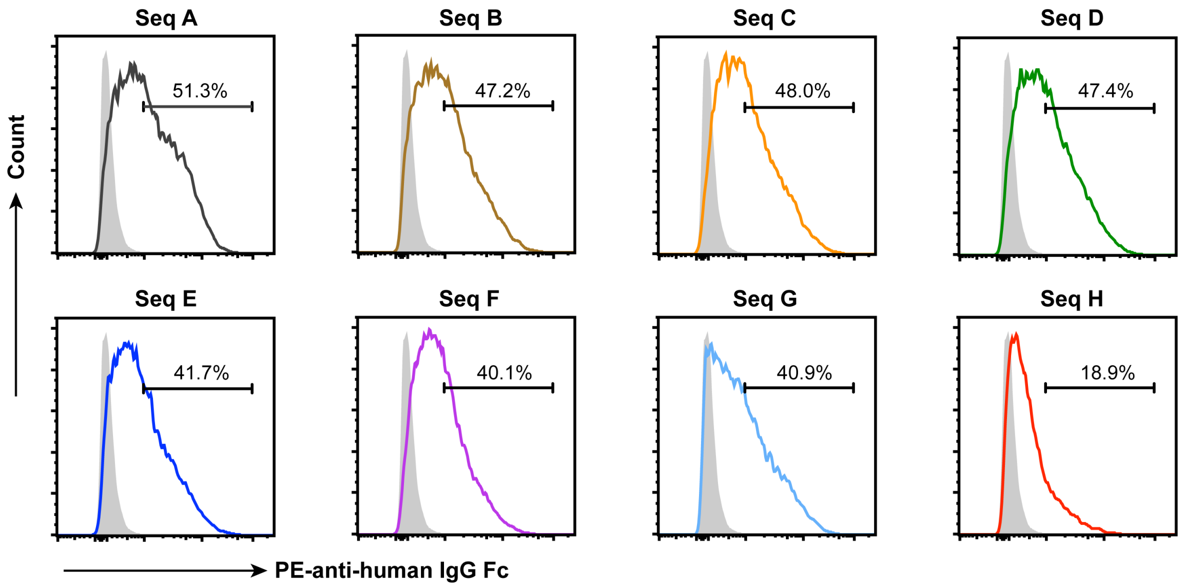

Supplementary Figure 12: Protein expression of Spike mRNAs in HEK293 cells. mRNAs were transfected into HEK293 cells and cells were harvested after 48 hours of incubation. Representative histograms show the surface expression of Spike antigen. Gray histogram represents cells stained by secondary antibody only (negative control).

UniProt ID	protein length	UniProt ID	protein length	UniProt ID	protein length	UniProt ID	protein length
P15421	78	Q6PF06	316	Q86TJ5	538	Q6UXY8	1006
P13501	91	Q13155	320	P07947	543	Q6ZRS4	1044
Q99525	98	Q9BYB4	327	P40222	546	Q8IZA0	1049
P31949	105	P78382	337	Q494X3	552	O75151	1096
P0CE72	109	A6NFE2	343	Q5T749	579	O60721	1099
P0CL80	117	P47211	349	Q8N1G4	583	Q15147	1175
P01707	119	Q3MIR4	351	Q9HA90	598	P54098	1239
O95867	125	Q9BY21	358	Q8N6Q8	603	Q6VMQ6	1270
Q9GZQ8	125	Q8TDG2	376	P31645	630	Q5TZJ5	1347
Q8NC38	126	P01860	377	Q6PI48	645	Q96HA7	1378
P15382	129	Q96DV4	380	Q9UNN5	650	Q5VYS8	1495
Q9NV29	134	Q16690	384	O75509	655	P51805	1871
O14880	152	Q92734	400	Q06187	659	K4DIA1	1962
P54852	163	A6NKF1	404	P05160	661	A7TUB1	2142
Q14442	188	Q5VV16	416	Q7Z340	670	E7EVM7	2703
Q15669	191	P15086	417	Q9H461	694	Q75N90	2809
Q9HAE3	211	Q9BY11	444	Q92542	709	B2RWP4	2826
Q7RTU1	214	Q5SWX8	454	Q96NI6	719	Q9NR99	2828
Q5T7N8	215	Q6NT16	456	P78563	741	P50851	2863
P49755	219	Q05901	458	Q9BXB4	747	Q3L8U1	2897
P98173	230	Q9NWZ3	460	Q9UFB7	747	Q59FF8	2966
P26436	265	Q15465	462	Q9UKQ2	775	H9A532	3038
P78345	283	Q9Y512	469	Q9HCI6	787	O75962	3097
P57076	290	Q6B0B8	471	C9J798	803	Q96L91	3159
A6NH11	291	Q9UBM8	478	Q9QC07	812	Q6ZRS2	3230
P30281	292	P47974	494	Q9H3R1	872	Q96JQ0	3298
Q8NH43	312	Q8IW19	511	Q8TF61	875	Q16787	3333
Q9HC38	313	Q8TAA9	524	Q8NB90	893		
Q8NGJ5	315	Q13087	525	Q9Y5G9	962		

Supplementary Table 1: Protein sequences used for runtime benchmarking in Fig. 3 and Supplementary Figure 8, and search error analysis in Supplementary Figure 9.

design	MFE ($kcal/mol$)	CAI	GC%	U%	Molecular weight	Mobi. shift relat. dist.	Mg^{2+}= $10mM$ half-life(h)	Mg^{2+}= $10mM$ $K(10^{-2})$	Mg^{2+}= $20mM$ half-life(h)	Mg^{2+}= $20mM$ $K(10^{-2})$	Protein expr. (MFI)	BAb titers	NAb titers	IFN-γ+ T cells
A	-2,287.3	0.756	54.8	22.7	1,229,541	**3.20**	**20.0**	3.46	**12.6**	5.52	**6,837**	11,404	217.9	**130.0**
B	-2,213.2	0.851	57.0	20.8	1,229,943	3.00	17.1	4.06	8.9	7.83	5,328	8,063	231.5	63.8
C	-2,206.0	0.757	55.3	22.2	1,230,380	2.97	11.1	6.25	7.2	9.58	5,939	**18,102**	**496.4**	98.5
D	-1,967.4	0.935	58.4	19.1	1,229,927	2.80	5.4	1.29	6.5	1.07	6,801	8,063	221.1	79.7
E	-1,961.3	0.851	56.8	21.1	1,229,328	2.72	5.5	1.26	6.4	1.09	4,499	2,540	68.2	52.7
F	-1,969.3	0.755	54.7	23.0	1,230,153	2.75	6.5	1.06	6.1	1.14	2,973	6,400	187.5	64.5
G	-1,639.3	0.935	58.6	18.9	1,229,526	2.50	5.4	1.28	4.8	1.45	6,446	2,851	64.0	70.5
H	-1,244.4	0.936	55.0	21.2	1,228,180	2.20	3.9	1.78	3.3	2.12	4,491	141	24.4	22.3

Supplementary Table 2: Detailed computational and experimental data for the 8 designs of COVID-19 mRNA vaccine. MFI: mean fluorescence intensity. See Supplementary Figure 10 for their secondary structures.

design	MFE ($kcal/mol$)	CAI	GC%	U%	Molecular weight	Mg^{2+}= 10mM half-life(h)	Mg^{2+}= 10mM $K(10^{-2})$	Mg^{2+}= 20mM half-life(h)	Mg^{2+}= 20mM $K(10^{-2})$	Protein expr. (MFI) (24h)	Protein expr. (MFI) (48h)	anti-gE IgG (Reciprocal titer)
gE-A	-1,145.6	0.787	58.1	20.1	602,907	66.5	1.04	50.7	1.37	7,886	3,220	16,890
gE-B	-1,082.9	0.844	59.8	18.8	602,330	39.1	1.78	18.7	3.72	19,736	10,632	77,605
gE-C	-932.3	0.901	60.5	17.2	603,570	22.2	3.12	9.5	7.32	20,950	11,585	67,559
gE-D	-845.4	0.918	60.4	16.9	602,793	14.0	4.96	7.9	8.74	22,457	13,098	22,286
gE-E	-805.0	0.930	61.0	16.3	602,478	12.1	5.71	7.4	9.42	23,246	4,958	44,572
gE-Ther	-592.2	0.937	58.5	17.4	602,225	10.9	6.35	5.9	1.18	9,257	2,457	9,701
gE-WT	-485.7	0.656	46.5	25.5	601,951	10.8	6.41	6.0	1.15	758	777	35

Supplementary Table 3: Detailed computational and experimental data for 7 sequences of VZV mRNA vaccine. Chemical stability of mRNAs upon incubation in buffer at 37 °C. MFI: mean fluorescence intensity. See Supplementary Figure 11 for their secondary structures.

Supplementary Sequence Information

Sequences used in COVID mRNA vaccine experiments

The mRNA sequences (coding region) used in the COVID mRNA vaccine experiments are listed below (stop codons not included). Sequences A–G are our designed mRNA sequences, H is the baseline sequence designed by conventional codon optimization method, and BNT is another baseline sequence from BioNTech/Pfizer COVID-19 vaccine (without “2P” mutations). In the experiments, we use two stop codons UAAUAG for all sequences.

```
>Sequence A: (MFE -2287.3 kcal/mol, CAI 0.756)
AUGUUCGUAUUCUUAGUGCUCCUGCCCCUGGUGUCCAGCCAGUGCGUGAACCUUACCACCCGGACCCAGUUGCCCCCCGCGUAUACGAACUCGUUUACGCGGGGGGU
GUACUAUCCGGAUAAGGUGUUCCGGAGCUCGGUUCUCCAUUCAACUCAGGACCUCUUCCUCCCGUUCUUCUCCAACGUGACAUGGUUCCAUGCCAUUCACGUUUCGG
GGACGAACGGGACGAAGAGGUUCGACAACCCCGUGUUGCCCUUCAACGACGGGGUGUACUUCGCCUCGACGGAGAAGAGCAACAUCAUACGGGGUUGGAUCUUUGGU
ACCACGUUGGAUAGCAAGACACAGUCAUUGUUGAUUGUGAACAAUGCUACCAACGUGGUUAUAAAGGUCUGUGAGUUCCAGUUCUGUAAUGACCCCUUCCUGGGGGU
CUAUUACCACAAGAACAACAAGUCAUGGAUGGAGAGCGAGUUCCGGGUGUACUCGAGUGCAAACAAUUGCACUUUCGAGUACGUGUCCCAGCCAUUCCUGAUGGAUC
UCGAAGGUAAGCAGGGGAACUUCAAGAAUCUGCGAGAGUUUGUGUUCAAGAACAUAGACGGGUACUUCAAGAUUUACAGUAAGCACACCCCGAUCAACUUGGUGCGG
GACUUACCCCAGGGCUUCAGCGCCCUGGAGCCGCUGGUGGACUUGCCGAUAGGCAUCAACAUCACGCGGUUCCAGACGCUGCUGGCCCUGCACCGGUCGUACCUCAC
CCCGGGGGACUCUUCCUCGGGGUGGACGGCCGGUGCAGCGGCGUACUAUGUAGGGUACCUGCAGCCACGCACGUUCUUGUUGAAGUACAACGAGAACGGCACAAUCA
CGGACGCCGUGGAUUGUGCGUUGGAUCCGCUUUCGGAGACAAAGUGUACACUGAAGAGCUUCACCGUGGAGAAGGGCAUCUACCAGACGUCCAACUUCAGGGUGCAG
CCGACCGAGUCGAUUGUGCGCUUCCCCAAUAUCACAAACCUGUGUCCCUUUGGGGAAGUGUUCAACGCGACUCGGUUCGCUUCAGUGUACGCUUGGAACCGAAAGCG
GAUCUCCAACUGCGUGGCUGACUACAGCGUGCUCUACAACAGUGCGUCGUUUAGCACGUUCAAGUGCUAUGGGGUGAGUCCCACCAAGUUGAACGACCUCUGCUUCA
CCAACGUCUACGCGGAUAGCUUCGUUAUCCGCGGAGACGAGGUGAGGCAGAUCGCUCCCGGUCAGACCGGGAAGAUCGCGGACUACAACUACAAGUUGCCGGACGAC
UUUACGGGUUGUGUUAUUGCUUGGAAUAGCAAUAACCUCGACUCGAAAGUCGGAGGUAAUUACAAUUACCUCUACAGACUGUUUCGGAAAAGCAAUCUGAAGCCCUU
CGAGCGUGAUAUAUCUACCGAGAUAUAUCAGGCUGGAUCCACCCCGUGCAACGGGGUGGAGGGCUUCAAUUGCUACUUUCCGUUACAGUCGUACGGCUUCCAGCCCA
CCAACGGGGUGGGCUACCAGCCGUACCGCGUGGUGGUGCUCAGCUUUGAGCUCCUCCACGCGCCGGCAACUGUAUGUGGUCCGAAGAAGUCGACGAACUUGGUGAAG
AACAAGUGCGUCAACUUCAACUUCAACGGUCUGACCGGGACGGGGGUGCUUACUGAGUCUAACAAGAAGUUCCUUCCCUUCCAACAGUUUGGAAGGGACAUCGCCGA
UACUACAGACGCGGUCCGGGAUCCCCAGACCCUGGAGAUCCUGGACAUCACGCCCUGUAGUUUCGGCGGUGUGUCUGUUAUCACCCCUGGGACGAACACCUCCAACC
AGGUGGCGGUGCUGUAUCAGGACGUGAAUUGCACCGAGGUGCCGGUGGCAAUUCACGCUGAUCAGCUGACCCCCACCUGGCGUGUAUAUAGUACAGGCUCGAACGUG
UUUCAGACACGUGCUGGGUGCCUGAUUGGCGCCGAGCACGUGAAUAACUCGUACGAGUGCGACAUUCCGAUCGGCGCGGGGAUUUGUGCGUCGUACCAGACGCAGAC
CAAUUCCCCGCGCCGAGCUCGGAGUGUCGCCUCCCAGAGCAUCAUCGCCUACACGAUGAGCCUGGGAGCGGAGAAUUCAGUGGCGUAUAGCAAUAACUCUAUUGCUA
UACCCACGAAUUUUACCAUCUCCGUGACAACGGAGAUUCUGCCGGUGUCUAUGACCAAGACAUCGGUAGACUGUACUAUGUACAUCUGCGGCGACAGUACUGAGUGC
AGUAACCUGUUGCUGCAGUAUGGGAGUUUUUGUACCCAGCUCAACCGCGCCCUGACUGGGAUCGCGGUUGAGCAGGACAAGAACACCCAGGAGGUGUUCGCCCAGGU
GAAGCAGAUAUACAAGACGCCCCCGAUCAAGGACUUCGGGGGCUUCAAUUUCUCGCAGAUUCUCCCGGAUCCGUCAAAGCCGAGCAAGAGGUCCUUUAUUGAGGACC
UCUUGUUCAACAAGGUGACUUUGGCGGAUGCGGGCUUCAUCAAGCAGUACGGUGACUGCCUGGGUGAUAUUGCUGCGCGCGAUCUGAUUUGCGCGCAGAAGUUCAAC
GGGCUCACCGUGCUGCCUCCCCUGCUUACCGACGAGAUGAUAGCGCAGUACACCUCUGCGCUGUUGGCUGGGACAAUCACGUCUGGCUGGACCUUCGGGGCAGGAGC
AGCACUCCAGAUCCCGUUCGCGAUGCAGAUGGCGUAUCGCUUCAACGGGAUCGGAGUGACCCAGAACGUACUCUAUGAGAAUCAGAAGCUGAUCGCCAACCAGUUCA
ACUCAGCUAUUGGCAAGAUCCAGGACAGCUUGUCCAGCACGGCCUCCGCGCUGGGCAAGCUCCAGGAUGUUGUCAAUCAGAACGCACAAGCGUUGAAUACGCUUGUG
AAGCAGCUGAGUUCGAACUUCGGGGCGAUCAGCUCUGUUCUCAACGACAUCCUGUCGCGGCUAGAUAAGGUGGAGGCCGAGGUUCAGAUCGACCGGUUGAUUACCGG
UCGAUUGCAGAGCCUCCAGACCUACGUAACUCAGCAGCUCAUCAGAGCUGCUGAGAUACGGGCCUCCGCCAAUCUAGCCGCGACAAAGAUGUCGGAGUGCGUUCUUG
GUCAGUCCAAGCGGGUGGACUUCUGCGGGAAGGGCUAUCACCUGAUGUCCUUCCCGCAGUCCGCCCCGCAUGGAGUAGUGUUUCUUCACGUCACUUAUGUACCGGCU
CAGGAGAAGAACUUCACGACUGCGCCUGCCAUCUGCCAUGAUGGCAAGGCGCAUUUCCCUCGUGAAGGCGUCUUCGUCAGCAACGGGACCCACUGGUUCGUCACGCA
GCGCAACUUCUAUGAGCCCCAGAUAAUUACCACUGACAAUACCUUUGUCAGUGGUAACUGUGACGUGGUCAUAGGGAUCGUGAACAACACUGUUUACGAUCCGCUCC
AGCCAGAGCUUGACAGCUUCAAGGAGGAGCUGGACAAGUACUUCAAGAAUCAUACUUCCCCAGACGUUGAUCUCGGAGACAUCUCCGGGAUCAACGCUUCUGUAGUG
AACAUACAGAAGGAGAUCGACAGGCUCAAUGAGGUUGCCAAGAACCUCAACGAGAGCCUGAUCGAUCUCCAGGAGCUGGGGAAGUAUGAGCAGUAUAUCAAGUGGCC
UUGGUAUAUCUGGCUGGGGUUUAUCGCGGGGCUCAUAGCGAUCGUGAUGGUCACGAUCAUGUUGUGCUGCAUGACGUCCUGCUGCAGCUGCCUGAAGGGCUGCUGCA
GCUGUGGGUCCUGUUGCAAGUUUGACGAAGACGAUUCUGAGCCGGUGCUGAAGGGCGUGAAGUUACACUACACC
```

>Sequence B: (MFE -2213.2 kcal/mol, CAI 0.851)

AUGUUCGUAUUUCUUGUGCUGCUUCCCCUCGUGUCAUCCCAGUGCGUGAACCUGACGACCAGAACCCAGCUGCCGCCAGCGUACACCAACAGCUUCACUCGGGGUGU
GUAUUACCCCGACAAGGUGUUCAGGUCCAGCGUGCUGCACAGCACGCAGGACCUGUUCCUGCCGUUCUUCUCCAACGUGACAUGGUUCCAUGCCAUUCACGUGUCUG
GGACCAAUGGUACGAAGCGCUUCGACAACCCGGUGUUGCCCUUCAACGAUGGGGUGUACUUCGCCUCAACGGAGAAGAGCAACAUCAUUCGAGGGUGGAUCUUCGGG
ACCACCCUCGACAGCAAGACUCAGAGCUUGCUCAUCGUGAACAACGCCACUAAUGUGGUGAUCAAGGUGUGCGAGUUCCAGUUUUGCAAUGACCCGUUCCUGGGGGU
GUACUAUCAUAAGAAUAACAAGUCCUGGAUGGAGAGUGAGUUCCGGGUGUACUCGAGUGCAAACAAUUGCACUUUCGAGUAUGUCAGUCAGCCCUUCCUGAUGGAUC
UGGAGGGGAAGCAGGGGAACUUCAAGAACCUGCGAGAGUUCGUGUUCAAGAACAUCGACGGGUACUUCAAGAUCUAUUCCAAGCACACGCCCAUCAACCUCGUGCGG
GACCUCCCGCAGGGGUUCUCUGCCCUGGAGCCUCUGGUGGACCUGCCUAUUGGCAUCAACAUCACCCGGUUUCAGACGCUCCUGGCCCUGCACAGGAGCUAUCUGAC
ACCGGGUGAUUCCAGCAGCGGCUGGACGGCCGGGGCUGCAGCCUACUACGUGGGCUACCUGCAGCCCCGGACCUUCUUGCUCAAGUACAACGAGAACGGCACAAUCA
CGGACGCCGUGGAUUGUGCCCUCGACCCCUUGAGCGAGACCAAGUGCACCCUGAAGAGCUUCACGGUGGAGAAGGGCAUCUACCAGACAAGCAACUUCAGGGUGCAG
CCAACCGAGUCGAUUGUGCGCUUCCCCAAUAUCACAAACCUGUGUCCCUUUGGGGAGGUGUUCAACGCGACUCGGUUCGCUAGCGUGUACGCAUGGAAUAGGAAGCG
AAUCAGCAACUGUGUCGCUGACUAUUCCGUGCUGUACAACAGCGCUAGCUUUAGCACUUUCAAGUGCUACGGCGUCUCCCCCACGAAGCUGAACGAUCUGUGUUUCA
CUAAUGUGUACGCAGACAGCUUCGUGAUCCGGGGAGACGAGGUCCGCCAGAUCGCUCCAGGGCAGACCGGUAAGAUCGCCGACUACAACUACAAGUUGCCGGACGAU
UUUACCGGGUGCGUGAUUGCUUGGAAUAGCAACAACCUGGACAGCAAGGUGGGAGGUAAUUACAAUUACCUCUACCGGCUGUUCAGGAAGUCCAACCUGAAGCCCUU
CGAGAGAGACAUCUCUACGGAGAUCUAUCAGGCUGGAUCCACCCCGUGCAACGGGGUGGAGGGGUUCAACUGCUACUUUCCCCUCCAGAGCUACGGCUUCCAGCCCA
CCAACGGGGUGGGCUACCAGCCGUACCGGGUGGUGGUGCUCUCCUUCGAGCUGCUGCACGCCCCCGCCACGGUGUGCGGCCCGAAGAAGAGCACCAACCUGGUGAAG
AAUAAGUGCGUGAACUUUAACUUCAACGGGCUGACUGGCACCGGCGUCCUGACUGAGAGCAAUAAGAAGUUCCUGCCAUUCCAGCAGUUUGGCAGGGACAUUGCUGA
CACCACGGACGCCGUCCGGGAUCCCCAGACCCUGGAGAUCCUGGACAUCACGCCAUGCUCCUUCGGGGGCGUGAGCGUGAUCACACCCGGAACUAACACCUCCAACC
AGGUCGCCGUCCUGUACCAGGACGUGAACUGCACGGAGGUGCCGGUGGCUAUACACGCCGACCAGCUGACCCCCACCUGGCGGGUGUAUUCCACCGGCAGCAACGUG
UUCCAGACACGUGCUGGGUGCCUGAUUGGCGCCGAGCACGUGAACAAUUCUUAUGAGUGUGACAUCCCCAUCGGAGCGGGCAUUUGCGCUUCGUACCAGACCCAGAC
CAACUCUCCACGGCGGGCCCGGUCAGUGGCCUCCCAGAGCAUCAUCGCCUACACGAUGAGCCUGGGCGCGGAGAAUAGUGUGGCGUAUAGCAAUAACUCUAUUGCUA
UACCCACCAACUUCACUAUCUCCGUGACCACAGAGAUACUGCCAGUCAGCAUGACGAAGACGUCUGUUGACUGCACCAUGUAUAUCUGUGGUGAUUCAACUGAGUGC
AGCAACCUGCUGCUGCAGUACGGCAGCUUCUGCACUCAGUUGAACAGGGCCCUGACCGGGAUCGCCGUGGAGCAGGACAAGAACACGCAGGAGGUGUUUGCUCAGGU
CAAGCAGAUCUACAAGACACCCCCCAUCAAGGACUUUGGGGGGUUCAACUUCAGUCAGAUCCUGCCUGACCCAAGCAAGCCGAGCAAGAGGUCCUUUAUUGAGGACC
UCUUGUUCAACAAGGUGACGCUGGCGGACGCUGGGUUCAUCAAGCAGUACGGUGACUGCCUGGGGGACAUCGCUGCGCGCGAUCUGAUUUGCGCGCAGAAGUUCAAC
GGGCUCACCGUGCUGCCCCCCCUGCUGACCGAUGAGAUGAUCGCUCAGUACACCAGCGCCCUGCUCGCUGGUACCAUUACCAGCGGGUGGACCUUCGGCGCUGGUGC
UGCAUUGCAGAUCCCUUUUGCAAUGCAGAUGGCGUACAGGUUCAACGGCAUUGGGGUGACACAGAACGUGCUGUACGAGAAUCAGAAGCUGAUCGCCAACCAGUUCA
ACUCGGCGAUCGGCAAGAUUCAGGACAGCCUGUCCAGCACCGCCUCCGCCCUGGGGAAGCUCCAGGACGUGGUCAAUCAGAACGCUCAGGCCCUGAAUACGCUGGUC
AAGCAGCUGAGCAGCAAUUUCGGGGCCAUCUCGAGCGUUCUGAACGACAUCCUGUCGCGGCUGGAUAAGGUGGAGGCCGAGGUGCAGAUCGACCGGCUGAUCACCGG
CCGGUUGCAGAGUCUGCAGACCUACGUGACUCAGCAGCUCAUCAGAGCUGCUGAGAUCAGGGCCUCCGCCAAUCUGGCCGCGACAAAGAUGUCUGAGUGCGUCCUGG
GGCAGUCCAAGCGGGUGGACUUCUGCGGGAAGGGCUAUCACCUGAUGUCCUUCCCGCAGUCCGCCCCGCAUGGAGUGGUCUUCCUGCACGUGACUUAUGUGCCGGCG
CAGGAGAAGAACUUCACCACUGCGCCGGCCAUUUGUCACGACGGGAAGGCCCACUUCCCCAGGGAGGGCGUCUUCGUCAGCAACGGGACCCACUGGUUUGUGACUCA
GAGAAACUUCUAUGAGCCACAGAUCAUUACCACUGACAAUACCUUUGUCAGUGGUAAUUGUGACGUGGUGAUCGGGAUCGUGAACAACACUGUUUACGAUCCCCUGC
AGCCCGAGCUGGACAGCUUCAAGGAGGAGCUGGACAAGUACUUCAAGAAUCAUACUUCCCCAGACGUUGAUCUCGGAGACAUCUCCGGGAUCAACGCUUCUGUGGUG
AACAUCCAGAAGGAGAUCGACAGGCUCAAUGAGGUGGCCAAGAACCUCAACGAGAGCCUGAUCGAUCUCCAGGAGCUGGGGAAGUAUGAGCAGUAUAUCAAGUGGCC
UUGGUAUAUCUGGCUCGGGUUCAUUGCAGGGCUGAUCGCCAUCGUCAUGGUGACGAUCAUGCUGUGCUGCAUGACGUCAUGCUGCAGCUGCCUGAAGGGCUGCUGCA
GCUGUGGGUCCUGUUGCAAGUUUGACGAGGACGACUCGGAGCCGGUGCUGAAGGGGGUGAAGCUGCACUACACC

>Sequence C:(MFE -2206.0 kcal/mol, CAI 0.757)

AUGUUUGUCUUCCUAGUGCUGCUGCCGCUGGUGAGUAGUCAGUGUGUGAACCUGACUACUCGCACCCAGCUGCCCCCCGCGUAUACGAACUCGUUUACGCGGGGGGU
GUACUACCCGGACAAAGUGUUCAGGUCCAGCGUGCUUCAUAGCACGCAGGACCUGUUCCUCCCGUUCUUCUCCAACGUGACAUGGUUCCAUGCCAUUCACGUUUCGG
GGACGAACGGGACGAAGCGCUUUGACAAUCCGGUGUUGCCCUUCAACGACGGGGUGUACUUCGCCUCGACGGAGAAGAGCAACAUCAUCCGGGGUUGGAUAUUCGGA
ACGACGCUGGACUCGAAGACCCAGUCACUGCUGAUCGUCAACAACGCGACGAACGUAGUGAUUAAGGUCUGCGAGUUCCAGUUCUGUAAUGACCCCUUCCUGGGGGU
CUAUUACCACAAGAACAACAAGUCCUGGAUGGAGUCGGAGUUUCGAGUGUACUCGAGUGCAAACAAUUGCACUUUCGAGUACGUUUCCCAGCCCUUUCUGAUGGAUU
UAGAAGGGAAGCAGGGAAACUUCAAGAACUUGAGGGAGUUCGUCUUUAAGAAUAUAGACGGAUACUUCAAGAUCUACUCGAAGCACACUCCGAUUAAUCUCGUCCGG
GACUUGCCGCAGGGCUUCAGCGCUCUGGAGCCGUUGGUGGACUUGCCGAUAGGCAUCAACAUCACUCGGUUCCAGACGCUGCUGGCCCUGCAUCGUUCCUAUCUAAC
ACCGGGUGACAGCAGCAGCGGCUGGACCGCCGGAGCAGCGGCUUACUAUGUGGGGUACCUGCAGCCCCGCACGUUCUUGUUGAAGUACAACGAGAACGGCACGAUCA
CCGACGCCGUCGACUGCGCUUUGGAUCCGUUGUCGGAGACAAAGUGCACGCUCAAGAGCUUCACGGUAGAGAAGGGCAUCUACCAGACCAGCAACUUCCGGGUCCAG

CCCACGGAGUCGAUUGUGCGCUUCCCCAAUAUCACAAACCUGUGUCCCUUUGGGGAAGUGUUCAACGCCACGCGGUUCGCUAGUGUGUAUGCGUGGAAUCGCAAGCG
CAUUAGCAACUGCGUGGCGGAUUAUUCAGUUUUGUACAACUCAGCCUCGUUCAGCACGUUCAAGUGCUACGGGGUGAGUCCUACAAAGCUGAAUGACCUCUGCUUCA
CCAACGUCUACGCGGAUAGCUUCGUUAUCCGCGGGGACGAGGUGAGGCAGAUCGCUCCGGGGCAGACCGGGAAGAUUGCUGACUACAACUAUAAGCUCCCGGAUGAC
UUCACCGGGUGCGUUAUAGCGUGGAAUUCGAACAACCUGGACAGCAAGGUAGGAGGUAAUUACAAUUACCUCUACCGGCUGUUCAGGAAGUCGAAUUUGAAGCCUUU
UGAGCGGGAUAUCUCGACGGAGAUAUACCAGGCUGGAAGCACGCCGUGCAAUGGAGUGGAAGGUUUCAACUGUUACUUUCCACUCCAGUCGUACGGCUUCCAGCCUA
CCAACGGGGUAGGGUACCAGCCCUACCGCGUUGUGGUGCUCUCCUUCGAGCUGCUCCACGCACCGGCCACGGUGUGUGGGCCGAAGAAGAGCACCAACUUGGUCAAG
AACAAGUGCGUGAACUUCAACUUUAAUGGGUUGACGGGCACGGGCGUUCUGACCGAGUCCAACAAGAAGUUCCUCCCGUUCCAGCAGUUCGGGAGGGACAUCGCGGA
CACGACGGACGCUGUCCGGGAUCCCCAGACCCUGGAGAUCCUGGACAUCACGCCGUGUUCGUUCGGCGGCGUCUCGGUGAUCACACCUGGUACGAACACUUCGAACC
AGGUGGCCGUGCUGUACCAAGAUGUAAAUUGUACGGAGGUUCCGGUGGCCAUUCACGCUGAUCAGCUUACUCCCACGUGGAGGGUGUACUCCACGGGGAGUAAUGUG
UUCCAGACACGUGCUGGGUGCCUGAUUGGCGCCGAGCACGUGAACAACUCUUAUGAGUGCGACAUUCCGAUCGGCGCGGGGAUUUGUGCGUCGUACCAGACGCAGAC
CAAUUCCCCGCGCCGAGCUCGGAGUGUCGCCUCCCAGAGCAUCAUCGCCUACACGAUGAGCCUGGGAGCGGAGAAUUCAGUGGCGUAUAGCAAUAACUCUAUUGCUA
UACCCACGAAUUUUACCAUCAGCGUGACCACCGAGAUCCUCCCAGUCAGCAUGACGAAGACGUCUGUUGACUGCACAAUGUACAUCUGUGGAGACAGCACGGAGUGC
UCGAACCUUCUGCUGCAGUACGGAUCCUUCUGUACUCAGCUGAAUCGAGCACUCACUGGCAUUGCCGUUGAGCAGGACAAGAACACCCAGGAGGUGUUUGCCCAGGU
CAAGCAGAUCUACAAGACACCUCCUAUCAAGGAUUUCGGGGGAUUCAACUUCUCCCAGAUCCUCCCCGAUCCCAGUAAACCGAGCAAGAGGUCCUUUAUUGAGGACC
UCUUGUUCAACAAAGUGACGCUGGCGGAUGCUGGGUUCAUCAAGCAGUACGGUGACUGCCUCGGGGAUAUUGCUGCGCGCGAUCUGAUUUGCGCGCAGAAGUUCAAC
GGGCUCACCGUGCUGCCGCCGUUGCUGACGGACGAGAUGAUCGCCCAGUACACGUCAGCGUUACUUGCCGGUACGAUCACAUCUGGGUGGACGUUCGGGGCUGGAGC
CGCGCUCCAGAUCCCGUUCGCCAUGCAGAUGGCGUACCGGUUUAAUGGGAUCGGGGUGACGCAGAAUGUGCUGUAUGAGAAUCAGAAGCUGAUUGCCAAUCAGUUCA
AUUCUGCGAUUGGCAAGAUCCAGGACAGCUUGUCCAGCACGGCCUCCGCGCUGGGCAAGCUCCAGGAUGUUGUCAAUCAGAAUGCUCAGGCGCUUAACACCCUCGUU
AAGCAGCUGAGCAGCAACUUCGGCGCCAUCUCCAGCGUGCUCAAUGACAUAUUGAGCAGGCUGGACAAGGUGGAGGCCGAAGUGCAGAUCGACCGGCUGAUCACGGG
GCGUCUGCAGAGCCUGCAGACGUACGUGACUCAGCAGUUGAUCCGCGCUGCGGAGAUCCGGGCGUCCGCCAACCUGGCGGCGACCAAGAUGUCCGAGUGCGUGCUGG
GGCAGUCGAAGCGGGUGGACUUCUGCGGGAAGGGCUAUCACCUGAUGUCCUUCCCGCAGUCCGCCCCGCACGGAGUUGUUUUCCUGCACGUGACUUAUGUCCCGGCG
CAGGAGAAGAACUUCACGACUGCCCCAGCAAUCUGUCACGACGGGAAGGCCCACUUCCCGCGUGAGGGUGUUUUUGUCUCCAACGGCACCCACUGGUUUGUCACCCA
GAGAAACUUCUAUGAGCCACAGAUAAUUACCACUGACAAUACCUUUGUAAGUGGUAAUUGCGAUGUGGUCAUAGGGAUCGUGAACAACACUGUUUACGAUCCUCUGC
AGCCGGAGCUGGACUCGUUCAAGGAAGAACUGGACAAGUACUUCAAGAACCACACCAGCCCGGAUGUAGAUCUGGGUGACAUCAGUGGGAUAAAUGCCAGUGUGGUC
AAUAUCCAGAAGGAGAUUGACCGACUCAAUGAGGUUGCCAAGAACCUCAAUGAGUCGCUCAUAGAUCUCCAGGAGCUGGGGAAGUAUGAGCAGUAUAUCAAGUGGCC
UUGGUAUAUUUGGCUCGGCUUCAUAGCCGGGCUCAUCGCGAUAGUGAUGGUGACGAUCAUGCUGUGCUGCAUGACGUCAUGCUGCAGCUGCCUGAAGGGCUGCUGCA
GCUGCGGUUCUUGUUGUAAGUUCGACGAGGACGAUAGUGAGCCGGUGCUCAAGGGGGUCAAGCUGCACUACACC

>Sequence D: (MFE -1967.4 kcal/mol, CAI 0.935)

AUGUUCGUAUUCCUGGUGCUGCUGCCCCUGGUGAGCAGCCAGUGCGUGAACCUGACCACACGCACUCAGCUGCCACCAGCGUACACCAACAGCUUCACCCGCGGGGU
GUACUACCCCGACAAGGUGUUCAGGUCCAGCGUGCUGCACAGCACGCAGGACCUGUUCCUGCCUUUCUUCAGCAACGUGACUUGGUUCCAUGCCAUUCACGUGAGUG
GCACCAAUGGAACCAAGCGCUUCGACAACCCUGUGCUGCCAUUUAAUGACGGCGUGUACUUCGCCAGCACCGAGAAGUCCAACAUCAUCAGAGGGUGGAUCUUUGGA
ACCACCCUGGACUCCAAGACCCAGAGCUUGCUCAUCGUGAACAACGCCACCAACGUGGUGAUCAAGGUGUGCGAGUUCCAGUUCUGUAAUGACCCCUUCCUGGGGGU
CUAUUACCACAAGAACAACAAGAGCUGGAUGGAGUCUGAGUUCAGGGUGUAUUCCAGCGCCAAUAAUUGCACAUUCGAGUACGUGUCUCAGCCCUUCCUGAUGGAUC
UGGAGGGGAAGCAGGGGAACUUCAAGAACCUGCGGGAGUUCGUGUUCAAGAACAUCGACGGGUACUUCAAGAUCUAUUCCAAGCACACACCCAUCAACCUGGUGCGC
GAUCUGCCUCAGGGCUUCAGCGCCCUGGAGCCCCUGGUGGACCUGCCCAUCGGCAUCAACAUCACCAGGUUCCAGACCCUGCUGGCCCUGCACAGGAGUUACCUGAC
UCCUGGCGACUCCAGCAGCGGCUGGACCGCCGGGGCUGCAGCCUACUACGUGGGCUACCUGCAGCCCCGGACCUUCCUGCUGAAGUACAACGAGAACGGCACCAUCA
CCGAUGCCGUGGACUGCGCCCUGGAUCCUCUGUCCGAGACAAAGUGUACACUGAAGAGCUUCACCGUGGAGAAGGGGAUCUACCAGACCUCCAACUUCAGGGUGCAG
CCAACCGAGAGCAUUGUGCGCUUCCCCAAUAUCACAAACCUGUGUCCCUUUGGGGAGGUGUUCAACGCUACUCGGUUCGCUUCAGUGUACGCUUGGAAUCGGAAGAG
GAUCAGCAAUUGCGUGGCUGAUUACAGCGUGCUGUACAACAGCGCCUCCUUCAGCACAUUCAAGUGCUACGGGGUGAGUCCCACAAAGCUGAACGAUCUGUGUUUCA
CCAACGUGUACGCAGACAGCUUUGUGAUCCGGGGCGAUGAGGUGAGGCAGAUCGCCCCCGGGCAGACCGGCAAGAUCGCCGACUACAACUACAAGCUGCCCGACGAC
UUCACCGGGUGUGUGAUUGCUUGGAAUAGCAACAACCUGGACAGCAAGGUGGGGGGCAACUACAACUACCUCUACCGGCUGUUCAGGAAGUCCAACCUGAAGCCCUU
UGAGAGAGACAUCUCUACAGAGAUCUAUCAGGCUGGCUCCACCCCAUGCAAUGGGGUGGAGGGGUUCAACUGCUACUUUCCCCUCCAGAGCUAUGGCUUCCAGCCCA
CCAACGGGGUGGGCUACCAGCCAUACCGGGUGGUGGUGCUCAGCUUUGAGCUGCUGCACGCCCCCGCCACCGUGUGCGGCCCAAAGAAGAGCACCAACCUGGUGAAG
AACAAGUGCGUGAACUUCAACUUCAAUGGCCUGACAGGCACCGGGGUGCUGACCGAGUCCAACAAGAAGUUCCUGCCAUUCCAGCAGUUUGGCAGGGACAUCGCCGA
CACUACAGAUGCCGUCCGGGAUCCCCAGACCCUGGAGAUCCUGGACAUCACCCCCUGUAGUUUCGGCGGGGUGAGCGUGAUCACCCCCGGCACAAACACCUCCAACC
AGGUGGCUGUGCUGUACCAGGACGUGAACUGCACCGAGGUGCCUGUGGCCAUCCACGCCGAUCAGCUGACCCCCACCUGGCGCGUGUACAGCACAGGCAGCAAUGUG
UUCCAGACACGCGCCGGGUGCCUGAUCGGCGCUGAGCACGUGAACAACAGCUACGAGUGUGACAUCCCUAUUGGCGCCGGGAUCUGCGCCAGCUACCAGACCCAGAC

CAACUCUCCACGGCGGGCCCGGUCAGUGGCCUCCCAGAGCAUCAUUGCCUACACAAUGAGCCUGGGGGCCGAGAACAGCGUGGCCUAUAGCAAUAACUCUAUUGCUA
UCCCCACAAAUUUUACAAUCAGCGUGACCACAGAGAUCUUGCCCGUGAGCAUGACCAAGACCUCUGUGGACUGCACCAUGUACAUUUGUGGGGACAGCACCGAGUGC
AGCAACCUGCUGCUGCAGUACGGCAGCUUCUGCACUCAGCUGAACAGGGCCCUGACCGGGAUCGCCGUGGAGCAGGACAAGAACACCCAGGAGGUGUUUGCCCAGGU
GAAGCAGAUCUACAAGACCCCCCCCAUCAAGGACUUCGGGGGGUUCAACUUCAGCCAGAUUCUGCCAGAUCCCUCCAAGCCCAGCAAGCGGUCCUUCAUCGAGGACC
UGCUGUUCAACAAGGUGACUCUGGCAGAUGCUGGCUUCAUCAAGCAGUACGGUGACUGCCUGGGGGACAUCGCGGCCAGAGACCUGAUCUGCGCCCAGAAGUUCAAC
GGGCUCACCGUGCUGCCCCCACUGCUGACUGACGAGAUGAUCGCUCAGUACACCAGUGCUCUGCUCGCCGGGACAAUCACCUCCGGGUGGACUUUUGGGGCUGGAGC
UGCCCUGCAGAUCCCCUUCGCCAUGCAGAUGGCCUAUCGGUUCAACGGCAUUGGGGUGACCCAGAACGUGCUGUACGAGAACCAGAAGCUGAUCGCCAAUCAGUUUA
AUAGCGCCAUCGGGAAGAUUCAGGACAGCCUGAGCAGCACCGCCAGCGCCCUGGGGAAGCUCCAGGACGUGGUGAACCAGAAUGCUCAGGCCCUGAAUACCCUGGUG
AAGCAGCUGUCCAGCAACUUCGGCGCCAUCUCCAGCGUGCUCAACGACAUCCUGAGCAGGCUGGACAAGGUGGAGGCCGAGGUGCAGAUCGAUCGGCUGAUCACAGG
UAGGCUGCAGAGCCUGCAGACCUACGUGACCCAGCAGCUGAUCCGGGCUGCAGAGAUUCGUGCCAGUGCCAACCUGGCCGCUACCAAGAUGAGCGAGUGUGUGCUGG
GACAGAGCAAGCGGGUGGACUUCUGUGGGAAGGGCUAUCACCUGAUGUCCUUCCCACAGUCCGCCCCUCACGGGGUGGUGUUCCUGCACGUGACCUACGUGCCCGCC
CAGGAGAAGAACUUCACCACCGCCCCCGCCAUCUGCCACGACGGGAAGGCCCACUUCCCUCGGGAGGGCGUGUUCGUGUCCAACGGCACACACUGGUUCGUGACCCA
GCGGAACUUUUACGAGCCCCAGAUCAUCACCACAGACAACACUUUCGUGAGUGGCAACUGUGACGUGGUGAUCGGGAUCGUGAACAACACUGUGUACGAUCCUCUGC
AGCCCGAGCUGGACAGCUUCAAGGAGGAGCUGGACAAGUACUUCAAGAACCACACCUCUCCUGAUGUGGACCUGGGUGACAUCAGCGGCAUCAACGCCAGCGUGGUG
AACAUCCAGAAGGAGAUCGACAGGCUGAAUGAGGUGGCCAAGAACCUCAACGAGAGCCUGAUCGAUCUCCAGGAGCUGGGGAAGUACGAGCAGUACAUUAAGUGGCC
CUGGUACAUCUGGCUGGGGUUCAUCGCCGGCCUGAUCGCCAUCGUGAUGGUGACCAUCAUGCUGUGCUGCAUGACCAGCUGCUGCAGCUGCCUGAAGGGCUGCUGCA
GCUGCGGGUCUUGCUGUAAGUUCGACGAGGACGACAGCGAGCCCGUGCUGAAGGGGGUGAAGCUGCAUUACACG

>Sequence E:(MFE -1961.3 kcal/mol, CAI 0.851)
AUGUUCGUUUUUUUAGUGCUGCUGCCCCUGGUGAGCUCUCAGUGCGUGAACCUGACCACGCGUACGCAGCUGCCCCCUGCGUACACCAACAGCUUCACACGGGGGGU
CUACUACCCUGACAAGGUGUUUAGAUCCUCCGUGCUGCACAGUACGCAGGACCUCUUCCUGCCGUUUUUCUCUAACGUGACAUGGUUCCAUGCCAUCCACGUUAGCG
GAACCAACGGCACGAAGAGGUUCGACAACCCCGUGCUGCCCUUCAACGACGGGGUGUACUUUGCCUCUACUGAGAAGAGUAACAUCAUCCGCGGGUGGAUCUUCGGC
ACUACCCUGGACUCGAAGACCCAGUCACUGCUGAUCGUGAACAACGCGACCAACGUGGUGAUUAAGGUCUGCGAGUUCCAGUUCUGUAAUGACCCCUUCCUGGGGGU
CUAUUACCACAAGAACAACAAGAGCUGGAUGGAGUCUGAGUUCAGAGUCUAUUCCAGCGCCAACAACUGCACCUUCGAGUACGUCUCUCAGCCCUUCCUGAUGGACC
UCGAGGGUAAGCAGGGCAACUUCAAGAACCUGCGGGAGUUCGUGUUCAAGAACAUUGAUGGGUACUUUAAGAUCUACUCUAAGCACACCCCCAUCAAUCUUGUGCGC
GAUCUCCCGCAGGGGUUCAGUGCCCUGGAGCCACUCGUGGAUCUGCCUAUUGGCAUCAACAUCACGCGGUUCCAGACCCUGCUGGCACUGCACAGGAGUUACCUGAC
CCCAGGUGACUCCUCCAGCGGGUGGACCGCUGGGGCAGCUGCGUACUACGUGGGUUAUCUUCAGCCCAGGACCUUCCUGCUGAAGUAUAACGAGAACGGCACCAUCA
CCGACGCCGUGGAUUGUGCCCUGGAUCCACUGUCAGAGACUAAGUGCACACUGAAGUCUUUCACAGUGGAGAAGGGCAUAUACCAGACCUCGAACUUCCGGGUCCAG
CCAACCGAGAGCAUUGUGCGCUUCCCCAAUAUCACAAACCUGUGUCCCUUUGGGGAGGUGUUCAACGCUACUCGGUUCGCUUCCGUGUACGCCUGGAACAGGAAGAG
AAUCAGCAACUGCGUUGCUGAUUACUCUGUGCUGUACAACAGCGCCUCCUUUAGCACUUUCAAGUGCUAUGGAGUGUCCCCCACGAAGCUGAACGAUCUGUGUUUCA
CUAAUGUGUACGCAGAUUCGUUCGUGAUUCGGGGGGACGAGGUGCGGCAGAUCGCUCCCGGGCAGACCGGGAAGAUCGCCGACUACAACUACAAGCUUCCAGACGAU
UUCACUGGCUGUGUGAUCGCCUGGAAUUCCAACAACCUUGACUCCAAGGUUGGGGGAAACUACAACUACCUGUACAGGCUGUUCCGGAAGUCCAACUUGAAGCCAUU
CGAGCGGGACAUCAGCACUGAGAUCUACCAGGCCGGCAGCACUCCAUGCAACGGCGUGGAGGGGUUUAACUGCUACUUCCCCCUGCAGAGCUACGGGUUUCAGCCCA
CCAACGGGGUGGGGUACCAGCCCUACCGCGUGGUGGUGCUGAGCUUCGAGCUGCUGCACGCCCCGGCGACGGUCUGCGGUCCCAAGAAGAGCACCAACCUGGUGAAG
AAUAAGUGCGUUAAUUUCAACUUUAACGGGCUUACCGGCACCGGGGUGCUCACGGAGAGCAACAAGAAGUUCUUGCCUUUCCAGCAGUUCGGCCGGGACAUUGCUGA
CACCACCGACGCUGUCCGGGAUCCCCAGACCCUGGAGAUCCUGGACAUUACUCCGUGUUCGUUCGGGGGGGUGAGCGUGAUCACCCCCGGAACGAACACGAGUAACC
AGGUGGCGGUGCUGUACCAGGAUGUCAACUGCACAGAGGUGCCAGUUGCCAUCCACGCCGAUCAGCUGACCCCCACCUGGCGGGUGUACAGCACCGGCUCGAACGUG
UUCCAGACACGUGCUGGGUGCCUGAUUGGCGCCGAGCACGUGAACAACUCGUACGAGUGCGACAUCCCCAUCGGGGCUGGCAUUUGUGCGAGUUACCAGACCCAGAC
CAACUCGCCGAGGCGUGCACGCUCGGUGGCCUCCCAGAGCAUCAUCGCCUACACGAUGAGCCUGGGAGCGGAGAAUAGCGUGGCGUAUAGCAAUAACAGCAUUGCUA
UACCCACGAAUUUCACCAUCUCCGUGACAACGGAGAUUCUGCCGGUGUCUAUGACCAAGACAUCGGUGGACUGCACGAUGUACAUCUGCGGCGACAGCACCGAGUGC
AGCAACCUGCUGCUGCAGUACGGAUCCUUCUGUACUCAGCUGAACAGGGCGCUGACUGGGAUUGCUGUCGAGCAGGACAAGAACACCCAGGAGGUGUUCGCCCAGGU
CAAGCAGAUCUACAAGACCCCCCCGAUAAAGGACUUCGGAGGGUUUAACUUCAGUCAGAUCCUGCCUGACCCAAGUAAGCCCUCCAAGAGGUCCUUUAUCGAGGACC
UGCUGUUCAACAAGGUGACCCUGGCUGAUGCCGGGUUCAUCAAGCAGUACGGUGACUGCCUGGGUGAUAUUGCUGCGCGCGACCUGAUCUGCGCGCAGAAGUUCAAC
GGGCUCACCGUGCUGCCCCCCCUGCUGACAGACGAGAUGAUUGCCCAGUACACAUCUGCUCUGUUAGCAGGGACCAUCACAUCUGGAUGGACGUUUGGUGCAGGGGC
CGCCCUGCAGAUCCCAUUCGCCAUGCAGAUGGCAUACCGGUUCAACGGAAUCGGUGUGACUCAGAACGUUCUGUACGAGAACCAGAAGCUGAUAGCUAACCAGUUCA
ACAGCGCUAUCGGUAAGAUUCAGGACUCACUGAGUUCCACCGCUAGCGCUCUGGGCAAGCUGCAGGACGUUGUGAACCAGAACGCCCAGGCGCUGAACACGCUGGUG
AAGCAGCUGUCCAGCAACUUCGGCGCCAUCUCGUCCGUGCUGAAUGACAUCCUGUCACGGCUGGACAAGGUGGAGGCCGAGGUGCAGAUCGACCGGCUGAUCACCGG
UAGGCUGCAGAGCCUGCAGACCUACGUGACCCAGCAGUUGAUCCGCGCCGCGGAGAUCCGGGCCUCCGCCAACCUGGCGGCCACAAAGAUGUCAGAGUGUGUCCUGG

GCCAGAGCAAGAGGGUGGACUUUUGCGGCAAGGGAUACCAUCUGAUGUCUUUCCCCCAGAGCGCCCCUCAUGGGGUGGUCUUCCUGCACGUGACUUAUGUGCCGGCG

CAGGAGAAGAACUUCACCACUGCGCCGGCCAUUUGUCACGACGGGAAGGCCCACUUUCCGCGGGAGGGGGUGUUCGUGAGCAACGGCACCCACUGGUUUGUGACUCA

GAGAAACUUCUAUGAGCCACAGAUCAUUACCACUGACAAUACCUUUGUCAGUGGUAAUUGCGACGUGGUGAUUGGGAUCGUGAACAACACCGUUUACGAUCCCCUCC

AGCCCGAGCUGGACUCCUUCAAGGAGGAGCUUGAUAAGUACUUCAAGAAUCACACGUCGCCGGACGUUGAUCUCGGAGACAUCUCCGGGAUCAACGCCUCCGUGGUC

AAUAUCCAGAAGGAGAUUGACCGGCUGAACGAGGUUGCCAAGAACCUGAACGAGAGUCUGAUUGACCUCCAGGAGCUUGGCAAGUACGAGCAGUACAUCAAGUGGCC

CUGGUACAUCUGGCUCGGCUUCAUUGCCGGGCUGAUUGCCAUCGUCAUGGUGACCAUCAUGUUGUGCUGCAUGACGAGCUGCUGCAGCUGCCUGAAGGGCUGCUGCA

GCUGCGGCUCGUGCUGCAAGUUCGACGAGGAUGACAGCGAGCCCGUGCUCAAGGGGGUGAAGCUGCAUUAUACC

>Sequence F:(MFE -1969.3 kcal/mol, CAI 0.755)

AUGUUCGUGUUCUUGGUCCUGCUCCCGUUGGUGUCCAGUCAGUGUGUUAAUCUGACCACCCGGACCCAGUUGCCCCCCGCGUAUACGAACUCGUUUACGCGGGGGGU

GUACUAUCCGGACAAGGUCUUCCGGUCGAGUGUGCUUCACUCGACCCAGGACCUCUUCCUCCCGUUUUUCUCUAACGUGACAUGGUUCCAUGCCAUUCACGUUAGUG

GAACUAACGGGACGAAGAGGUUUGAUAAUCCUGUUCUUCCGUUCAACGACGGGGUGUACUUCGCGUCGACGGAGAAGAGCAAUAUUAUCAGAGGGUGGAUCUUCGGG

ACCACCCUCGACAGUAAGACGCAGUCGCUUCUUAUUGUCAACAACGCCACUAAUGUGGUGAUCAAGGUUUGUGAGUUCCAGUUCUGUAAUGACCCCUUCCUUGGGGU

CUAUUACCACAAGAACAACAAAUCUUGGAUGGAGUCGGAGUUCCGAGUCUAUAGCAGCGCCAACAACUGCACGUUCGAGUACGUGUCCCAGCCAUUCCUUAUGGACC

UGGAGGGUAAGCAGGGUAAUUUUAAGAACCUGCGAGAGUUCGUGUUCAAGAACAUCGACGGGUAUUUUAAAAUUUACUCUAAGCAUACCCCUAUUAAUCUCGUUCGG

GAUCUCCCCCAGGGGUUCAGUGCUCUGGAGCCCCUGGUGGAUUUGCCGAUAGGGAUUAAUAUCACGCGUUUCCAGACGCUGUUGGCGCUGCACCGGAGUUACCUGAC

UCCGGGGGAUAGCUCAUCAGGCUGGACUGCUGGUGCCGCGGCGUACUAUGUGGGGUACCUCCAGCCCCGGACGUUCUUGUUGAAGUACAACGAGAACGGCACGAUCA

CUGACGCGGUGGACUGCGCCCUGGAUCCGCUUUCGGAGACCAAGUGUACUCUCAAGAGCUUCACCGUGGAGAAGGGGAUCUACCAGACCUCUAACUUCAGGGUGCAG

CCUACUGAGAGUAUUGUUCGGUUCCCGAACAUCACGAACUUAUGCCCGUUCGGGGAGGUGUUCAACGCCACCCGAUUCGCGAGCGUAUACGCGUGGAACCGAAAGCG

GAUCAGCAAUUGUGUGGCUGACUACUCGGUGCUGUACAACAGCGCCAGUUUCAGCACAUUCAAGUGCUACGGGGUGAGUCCAACCAAGCUCAAUGAUCUGUGCUUCA

CGAAUGUCUACGCUGACAGCUUCGUGAUCCGGGGCGACGAGGUGCGCCAGAUAGCGCCGGGGCAGACCGGCAAGAUCGCCGACUACAAUUAUAAGCUCCCCGACGAC

UUCACAGGUUGUGUUAUUGCCUGGAAUAGCAACAACCUGGAUAGUAAGGUCGGGGGGAAUUAUAAUUACCUGUACCGCCUGUUCCGGAAGUCCAACUUGAAGCCAUU

CGAGCGGGACAUCUCAACGGAGAUAUAUCAGGCUGGAUCCACUCCAUGCAACGGCGUGGAGGGAUUCAACUGUUAUUUUCCGUUGCAGUCGUACGGGUUCCAACCGA

CGAAUGGGGUCGGGUACCAGCCGUACCGCGUGGUGGUGCUCUCCUUUGAGCUCCUCCACGCGCCGGCUACGGUAUGCGGCCCCAAGAAGUCGACUAAUCUUGUGAAG

AACAAGUGUGUCAACUUCAACUUUAAUGGGUUGACGGGAACAGGCGUGCUGACGGAGAGCAACAAGAAGUUCCUCCCGUUCCAGCAGUUCGGCCGGGAUAUCGCCGA

CACCACGGAUGCCGUCCGGGAUCCCCAGACCCUGGAGAUCCUGGACAUUACUCCGUGUUCGUUCGGGGGGGUGAGCGUGAUCACCCCCGGAACGAACACGAGUAACC

AGGUUGCCGUCCUGUACCAGGACGUCAACUGCACCGAGGUGCCGGUUGCUAUACACGCGGACCAGUUGACCCCAACUUGGCGCGUGUAUAGCACGGGCUCGAACGUG

UUUCAGACACGUGCUGGGUGCCUGAUUGGCGCCGAGCACGUGAAUAACUCGUACGAGUGCGAUAUCCCCAUUGGAGCUGGCAUCUGCGCCAGCUACCAGACGCAGAC

GAACAGUCCGCGUCGCGCGCGGAGCGUUGCGUCACAGAGUAUCAUCGCCUACACGAUGUCGUUAGGCGCUGAGAACUCUGUGGCGUACAGUAACAAUUCGAUCGCCA

UCCCGACGAAUUUUACGAUCUCCGUGACAACGGAGAUCCUGCCGGUGAGCAUGACGAAGACCUCCGUAGACUGUACCAUGUACAUCUGCGGAGACUCUACGGAGUGC

UCAAACCUGCUGCUCCAGUACGGGAGCUUCUGCACUCAGCUGAACCGAGCCCUCACGGGUAUUGCUGUCGAGCAGGACAAGAAUACCCAGGAGGUGUUCGCCCAGGU

GAAGCAGAUCUACAAGACUCCUCCGAUAAAGGACUUUGGAGGGUUUAACUUCUCGCAGAUCCUGCCAGAUCCCUCGAAGCCCUCCAAACGUUCAUUUAUCGAGGACC

UCUUGUUCAACAAGGUGACGCUGGCUGAUGCCGGCUUCAUCAAGCAGUACGGUGACUGCCUGGGUGAUAUUGCUGCGCGCGACCUGAUCUGCGCGCAGAAGUUCAAC

GGGCUCACCGUGCUGCCCCCCCUGCUGACGGACGAGAUGAUCGCCCAGUACACGUCGGCCCUGCUGGCCGGAACGAUCACCAGCGGGUGGACGUUCGGCGCAGGGGC

GGCGCUCCAGAUACCGUUCGCCAUGCAGAUGGCGUAUCGGUUCAAUGGAAUCGGGGUUACCCAGAACGUUCUCUACGAGAAUCAGAAGCUGAUUGCCAAUCAGUUUA

AUUCUGCGAUAGGGAAGAUUCAGGAUAGCCUGAGUUCAACGGCGAGCGCGUUGGGUAAGCUGCAGGACGUAGUUAACCAGAACGCGCAGGCCUUGAACACGCUGGUG

AAGCAGCUCUCGAGUAAUUUCGGGGCUAUCAGCAGCGUUCUAAAUGAUAUUCUGUCUCGACUUGACAAAGUCGAGGCAGAAGUUCAGAUAGAUCGGCUGAUCACGGG

GCGGCUGCAGUCGUUACAGACCUAUGUGACGCAGCAGCUGAUCCGUGCAGCCGAGAUCCGUGCCUCAGCUAAUCUAGCCGCUACGAAGAUGUCCGAGUGCGUUCUGG

GACAGUCGAAGCGGGUAGAUUUCUGUGGCAAGGGUUAUCAUUUGAUGAGCUUUCCCCAGUCCGCCCCUCAUGGGGUGGUCUUCCUGCACGUGACUUAUGUGCCGGCG

CAGGAGAAGAACUUCACCACUGCGCCGGCCAUAUGUCACGACGGGAAGGCUCAUUUCCCGCGCGAGGGCGUGUUCGUGUCUAAUGGCACGCACUGGUUCGUGACGCA

GCGGAACUUCUAUGAGCCUCAGAUUAUCACAACUGACAACACCUUCGUGAGCGGGAAUUGCGAUGUCGUGAUAGGCAUCGUAAAUAAUACAGUUUAUGACCCACUUC

AGCCGGAGCUCGACUCCUUCAAGGAGGAGCUGGAUAAGUAUUUCAAGAAUCACACGUCGCCGGACGUGGAUUUGGGAGAUAUAUCCGGCAUCAACGCCUCGGUGGUC

AAUAUCCAGAAGGAGAUUGACCGCCUGAACGAGGUGGCCAAGAACCUGAACGAGAGUCUGAUUGACCUCCAGGAGCUUGGCAAGUACGAGCAGUAUAUCAAGUGGCC

UUGGUAUAUCUGGUUGGGCUUCAUAGCAGGCCUUAUAGCAAUUGUCAUGGUGACGAUCAUGCUGUGCUGCAUGACGUCAUGCUGCAGUUGCUUGAAGGGCUGCUGUA

GCUGUGGUUCUUGUUGUAAGUUCGACGAGGACGACAGCGAGCCCGUGCUGAAGGGGGUCAAGCUGCAUUAUACC

>Sequence G: (MFE -1639.3 kcal/mol, CAI 0.935)

AUGUUCGUAUUCCUAGUGCUGCUGCCCCUGGUGAGCAGCCAGUGCGUGAACCUGACCACACGCACUCAGCUGCCACCAGCCUACACCAACUCAUUCACCAGGGGCGU

GUACUAUCCUGACAAGGUGUUCAGGAGCAGCGUGCUGCACUCCACCCAGGAUCUGUUCCUGCCCUUCUUCAGCAACGUGACAUGGUUCCACGCCAUCCACGUGAGCG
GGACAAACGGCACCAAGCGGUUUGACAAUCCCGUUCUGCCAUUCAACGAUGGCGUGUACUUCGCCAGCACCGAGAAGAGCAACAUCAUCCGGGGCUGGAUCUUCGGC
ACCACACUGGACAGCAAGACCCAGAGUCUGCUGAUCGUGAACAACGCCACCAACGUGGUGAUCAAGGUGUGUGAGUUCCAGUUCUGUAAUGACCCCUUCCUGGGGGU
CUAUUACCACAAGAACAAUAAAAGCUGGAUGGAGAGCGAGUUUCGGGUGUACAGUAGUGCCAACAACUGUACAUUCGAGUACGUGUCCCAGCCAUUCCUGAUGGACC
UGGAGGGGAAGCAGGGGAACUUCAAGAACCUGCGGGAGUUCGUGUUUAAGAAUAUCGACGGAUAUUUUAAGAUCUACUCCAAGCACACCCCCAUCAACCUGGUGCGG
GAUCUGCCCCAGGGCUUCAGCGCCCUGGAGCCCCUGGUGGACCUGCCCAUCGGGAUCAACAUCACCCGGUUCCAGACCCUGCUGGCCCUGCACCGGAGUUACCUGAC
CCCAGGUGACUCCAGCAGCGGCUGGACAGCCGGGGCUGCAGCCUACUACGUGGGCUACCUGCAGCCCCGGACCUUCCUCCUGAAGUACAAUGAGAACGGCACCAUCA
CCGAUGCCGUGGAUUGUGCUCUGGACCCCCUGUCUGAGACCAAGUGCACCCUGAAGAGCUUCACCGUGGAGAAGGGGAUCUACCAGACAAGCAACUUUCGGGUGCAG
CCCACCGAGAGUAUCGUGAGGUUCCCCAACAUCACCAACCUGUGUCCAUUCGGGGAGGUGUUCAACGCCACCCGGUUCGCAAGCGUGUACGCUUGGAACCGGAAGCG
CAUCAGCAAUUGCGUGGCUGAUUACAGCGUGCUGUACAACAGCGCCUCCUUCAGCACAUUCAAGUGCUACGGCGUCUCCCCCACAAAGCUGAACGAUCUGUGUUUCA
CCAACGUGUACGCAGACAGCUUUGUGAUCCGGGGAGACGAGGUGCGGCAGAUCGCACCCGGGCAGACCGGCAAGAUUGCUGACUACAAUUACAAGCUGCCUGAUGAC
UUCACAGGCUGCGUGAUUGCCUGGAACAGCAAUAACCUGGACAGCAAGGUGGGAGGUAAUUACAAUUACCUCUACCGGCUGUUCAGGAAGUCCAAUCUGAAGCCCUU
CGAGAGGGACAUCUCCACCGAGAUCUACCAGGCUGGGUCCACCCCAUGCAAUGGGGUGGAGGGCUUCAACUGCUACUUCCCCCUGCAGUCCUACGGCUUCCAGCCCA
CCAAUGGGGUGGGCUACCAGCCCUACCGGGUGGUGGUGCUGAGCUUCGAGCUCCUGCACGCCCCCGCCACUGUGUGCGGCCCCAAGAAGUCCACCAACCUGGUGAAG
AACAAGUGCGUGAACUUCAACUUCAACGGGUUGACCGGGACUGGGGUGCUCACAGAGAGCAACAAGAAGUUCCUGCCCUUCCAGCAGUUCGGAAGGGACAUCGCCGA
CACCACGGAUGCCGUGAGGGAUCCCCAGACCCUCGAGAUCCUGGACAUCACGCCAUGCUCCUUCGGGGGCGUGAGCGUGAUCACUCCUGGCACAAACACCUCCAACC
AGGUGGCUGUGCUGUACCAGGACGUGAACUGCACCGAGGUGCCUGUGGCCAUCCAUGCUGACCAGCUGACCCCCACCUGGCGGGUCUACAGCACCGGCAGCAACGUG
UUCCAGACCCGGGCCGGCUGCCUGAUCGGGGCCGAGCACGUGAACAACAGCUACGAGUGCGAUAUCCCCAUCGGCGCCGGCAUCUGCGCUAGCUACCAGACACAGAC
CAAUAGCCCCAGACGGGCUAGGUCUGUGGCUAGCCAGAGCAUCAUCGCCUACACUAUGUCUCUGGGCGCAGAGAAUAGUGUGGCUUACAGCAACAACAGCAUCGCCA
UUCCCACUAAUUUCACAAUUAGUGUGACCACCGAGAUCCUGCCUGUGAGCAUGACCAAGACCAGCGUGGACUGCACCAUGUACAUCUGCGGGGAUAGCACCGAGUGC
AGCAACCUGCUGCUGCAGUACGGCAGCUUCUGCACACAGCUGAACCGGGCCCUGACCGGUAUCGCUGUGGAGCAGGACAAGAACACCCAGGAGGUGUUUGCGCAGGU
GAAGCAGAUCUACAAGACCCCUCCUAUCAAGGACUUCGGGGGCUUUAACUUCAGCCAGAUCCUGCCAGACCCCUCCAAGCCCUCCAAGAGGAGCUUCAUCGAGGAUC
UCCUGUUCAACAAGGUGACCCUGGCCGAUGCCGGCUUCAUCAAGCAGUACGGAGAUUGCCUGGGCGACAUCGCCGCCAGGGAUCUCAUCUGCGCCCAGAAGUUCAAC
GGCCUGACCGUGCUGCCCCCCCUGCUGACCGAUGAGAUGAUCGCUCAGUACACCAGCGCCCUGCUGGCUGGGACCAUCACCAGCGGGUGGACCUUCGGGGCCGGCGC
CGCCCUGCAGAUCCCCUUCGCCAUGCAGAUGGCCUACCGGUUCAACGGUAUUGGGGUGACCCAGAAUGUGCUGUAUGAGAACCAGAAGCUGAUCGCCAACCAGUUCA
ACUCUGCCAUCGGCAAGAUCCAGGACUCCCUGAGCAGCACUGCCAGCGCCCUGGGCAAGCUGCAGGACGUGGUGAACCAGAACGCCCAGGCCCUGAAUACCCUGGUG
AAGCAGCUGUCCAGCAACUUCGGGGCCAUCAGCAGCGUGCUGAACGACAUCCUGUCCCGCCUGGACAAGGUGGAGGCAGAGGUGCAGAUCGAUCGGCUGAUCACAGG
UAGGCUGCAGAGCCUGCAGACCUACGUGACCCAGCAGCUGAUCCGCGCCGCUGAGAUUAGGGCCAGCGCCAACCUGGCCGCCACUAAGAUGAGCGAGUGCGUGCUGG
GCCAGAGCAAGCGGGUGGACUUCUGCGGCAAGGGGUAUCACCUGAUGAGCUUCCCUCAGUCCGCCCCCCACGGGGUGGUGUUCCUGCACGUGACAUACGUGCCCGCC
CAGGAGAAGAACUUCACCACCGCCCCUGCCAUCUGCCACGAUGGCAAGGCCCACUUUCCCCGGGAGGGCGUGUUCGUGUCCAAUGGCACACAUUGGUUCGUGACCCA
GCGGAACUUCUACGAGCCACAGAUCAUUACCACUGACAAUACCUUUGUCAGUGGUAACUGUGACGUGGUGAUCGGCAUUGUGAACAACACAGUGUACGAUCCUCUGC
AGCCAGAGCUGGACAGCUUCAAGGAGGAGCUGGACAAGUACUUCAAGAACCACACCAGCCCCGACGUGGAUCUUGGAGACAUCUCUGGCAUCAACGCCAGCGUGGUC
AACAUCCAGAAGGAGAUUGACCGCCUGAACGAGGUUGCCAAGAACCUGAACGAGAGUCUGAUCGACCUCCAGGAGCUUGGCAAGUACGAGCAGUACAUCAAGUGGCC
AUGGUACAUCUGGCUGGGCUUCAUCGCCGGCCUGAUCGCCAUCGUGAUGGUGACCAUCAUGCUGUGCUGCAUGACCUCUUGUUGCAGCUGCCUGAAGGGGUGCUGCA
GCUGCGGUAGUUGCUGUAAGUUCGACGAGGACGACAGCGAGCCCGUGCUGAAGGGCGUGAAGCUGCACUACACG

>Sequence H: (MFE -1244.4 kcal/mol, CAI 0.936)

AUGUUCGUCUUCCUGGUGCUGCUGCCUCUGGUGUCUUCCCAGUGCGUGAAUCUGACUACCAGGACCCAGCUGCCCCCUGCCUAUACCAAUUCCUUCACACGGGGCGU
GUACUAUCCCGACAAGGUGUUUAGAAGCUCCGUGCUGCACUCUACACAGGAUCUGUUUCUGCCUUUCUUUAGCAACGUGACCUGGUUCCACGCCAUCCACGUGAGCG
GCACCAAUGGCACAAAGCGGUUCGACAAUCCAGUGCUGCCCUUUAACGAUGGCGUGUACUUCGCCUCUACCGAGAAGAGCAACAUCAUCAGAGGCUGGAUCUUUGGC
ACCACACUGGACUCCAAGACACAGUCUCUGCUGAUCGUGAACAAUGCCACCAACGUGGUCAUCAAGGUGUGCGAGUUCCAGUUUUGUAAUGAUCCAUUCCUGGGCGU
GUACUAUCACAAGAACAAUAAGAGCUGGAUGGAGUCCGAGUUUCGCGUGUAUUCUAGCGCCAACAAUUGCACAUUUGAGUACGUGUCCCAGCCCUUCCUGAUGGACC
UGGAGGGCAAGCAGGGCAAUUUCAAGAACCUGAGGGAGUUCGUGUUUAAGAAUAUCGAUGGCUACUUCAAGAUCUACUCUAAGCACACCCCAAUCAACCUGGUGCGC
GACCUGCCACAGGGCUUCAGCGCCCUGGAGCCACUGGUGGAUCUGCCCAUCGGCAUCAACAUCACCCGGUUUCAGACACUGCUGGCCCUGCACAGAAGCUACCUGAC
ACCAGGCGACUCCUCUAGCGGAUGGACCGCAGGAGCAGCAGCCUACUAUGUGGGCUAUCUGCAGCCCAGGACCUUCCUGCUGAAGUACAACGAGAAUGGCACCAUCA
CAGACGCCGUGGAUUGCGCCCUGGAUCCCCUGAGCGAGACAAAGUGUACACUGAAGUCCUUUACCGUGGAGAAGGGCAUCUAUCAGACAUCCAAUUUCAGGGUGCAG
CCUACCGAGUCUAUCGUGCGCUUUCCCAAUAUCACAAACCUGUGCCCUUUUGGCGAGGUGUUCAACGCAACCAGGUUCGCAAGCGUGUACGCAUGGAAUAGGAAGCG
CAUCUCUAACUGCGUGGCCGACUAUAGCGUGCUGUACAACUCCGCCUCUUUCAGCACCUUUAAGUGCUAUGGCGUGUCCCCCACAAAGCUGAAUGACCUGUGCUUUA

CCAACGUGUACGCCGAUUCUUUCGUGAUCAGGGGCGACGAGGUGCGCCAGAUCGCACCAGGACAGACAGGCAAGAUCGCAGACUACAAUUAUAAGCUGCCUGACGAU
UUCACCGGCUGCGUGAUCGCCUGGAACAGCAACAAUCUGGAUUCCAAAGUGGGCGGCAACUACAAUUAUCUGUACCGGCUGUUUAGAAAGAGCAAUCUGAAGCCAUU
CGAGAGGGACAUCUCUACAGAGAUCUACCAGGCAGGAAGCACCCCAUGCAAUGGAGUGGAGGGCUUUAACUGUUAUUUCCCUCUGCAGUCCUACGGCUUCCAGCCAA
CCAACGGCGUGGGCUAUCAGCCCUACCGCGUGGUGGUGCUGAGCUUUGAGCUGCUGCACGCACCUGCAACAGUGUGCGGACCAAAGAAGUCCACCAAUCUGGUGAAG
AACAAGUGCGUGAACUUCAACUUCAACGGCCUGACCGGAACAGGCGUGCUGACCGAGUCCAACAAGAAGUUCCUGCCUUUUCAGCAGUUCGGCAGGGACAUCGCAGA
UACCACAGACGCCGUGCGCGACCCUCAGACCCUGGAGAUCCUGGAUAUCACACCAUGCUCUUUCGGCGGCGUGAGCGUGAUCACACCAGGCACCAAUACAAGCAACC
AGGUGGCCGUGCUGUAUCAGGACGUGAAUUGUACCGAGGUGCCAGUGGCAAUCCACGCAGAUCAGCUGACCCCUACAUGGCGGGUGUACAGCACCGGCUCCAACGUG
UUCCAGACAAGAGCAGGAUGUCUGAUCGGAGCAGAGCACGUGAACAAUUCCUAUGAGUGCGACAUCCCUAUCGGCGCCGGCAUCUGUGCCUCUUACCAGACCCAGAC
AAACUCUCCAAGGAGAGCACGGAGCGUGGCAUCCCAGUCUAUCAUCGCCUAUACCAUGUCCCUGGGCGCCGAGAAUUCUGUGGCCUACUCUAACAAUAGCAUCGCCA
UCCCUACCAACUUCACAAUCUCUGUGACCACAGAGAUCCUGCCAGUGUCCAUGACCAAGACAUCUGUGGACUGCACAAUGUAUAUCUGUGGCGAUUCUACCGAGUGC
AGCAACCUGCUGCUGCAGUACGGCAGCUUUUGUACCCAGCUGAAUAGAGCCCUGACAGGCAUCGCCGUGGAGCAGGAUAAGAACACACAGGAGGUGUUCGCCCAGGU
GAAGCAGAUCUACAAGACCCCACCCAUCAAGGACUUUGGCGGCUUCAAUUUUUCCCAGAUCCUGCCCGAUCCUUCCAAGCCCUCUAAGCGGAGCUUUAUCGAGGACC
UGCUGUUCAACAAGGUGACCCUGGCCGAUGCCGGCUUCAUCAAGCAGUAUGGCGAUUGCCUGGGCGACAUCGCAGCACGGGACCUGAUCUGUGCCCAGAAGUUUAAU
GGCCUGACCGUGCUGCCUCCACUGCUGACAGAUGAGAUGAUCGCACAGUACACAAGCGCCCUGCUGGCAGGAACCAUCACAUCCGGAUGGACCUUCGGCGCAGGAGC
CGCCCUGCAGAUCCCCUUUGCCAUGCAGAUGGCCUAUCGGUUCAACGGCAUCGGCGUGACCCAGAAUGUGCUGUACGAGAACCAGAAGCUGAUCGCCAAUCAGUUUA
ACUCCGCCAUCGGCAAGAUCCAGGACAGCCUGUCCUCUACAGCCUCCGCCCUGGGCAAGCUGCAGGAUGUGGUGAAUCAGAACGCCCAGGCCCUGAAUACCCUGGUG
AAGCAGCUGAGCUCCAACUUCGGCGCCAUCUCUAGCGUGCUGAAUGAUAUCCUGAGCCGGCUGGACAAGGUGGAGGCAGAGGUGCAGAUCGACCGGCUGAUCACAGG
CAGACUGCAGUCUCUGCAGACCUAUGUGACACAGCAGCUGAUCAGGGCAGCAGAGAUCAGGGCAAGCGCCAAUCUGGCAGCAACCAAGAUGUCCGAGUGCGUGCUGG
GCCAGUCUAAGAGAGUGGACUUUUGUGGCAAGGGCUAUCACCUGAUGUCCUUCCCACAGUCUGCCCCUCACGGAGUGGUGUUUCUGCACGUGACCUACGUGCCAGCC
CAGGAGAAGAACUUCACCACAGCACCAGCAAUCUGCCACGAUGGCAAGGCACACUUUCCUAGGGAGGGCGUGUUCGUGUCCAACGGCACCCACUGGUUUGUGACACA
GCGCAAUUUCUACGAGCCACAGAUCAUCACCACAGACAAUACCUUCGUGAGCGGCAACUGUGACGUGGUCAUCGGCAUCGUGAACAAUACCGUGUAUGAUCCUCUGC
AGCCAGAGCUGGACAGCUUUAAGGAGGAGCUGGAUAAGUACUUCAAGAAUCACACCUCCCCCGACGUGGAUCUGGGCGACAUCAGCGGCAUCAAUGCCUCCGUGGUG
AACAUCCAGAAGGAGAUCGACAGGCUGAACGAGGUGGCCAAGAAUCUGAACGAGAGCCUGAUCGAUCUGCAGGAGCUGGGCAAGUAUGAGCAGUACAUCAAGUGGCC
UUGGUACAUCUGGCUGGGCUUCAUCGCCGGCCUGAUCGCCAUCGUGAUGGUGACCAUCAUGCUGUGCUGUAUGACAUCCUGCUGUUCUUGCCUGAAGGGCUGCUGUA
GCUGCGGCUCCUGUUGUAAAUUCGAUGAGGAUGAUUCCGAGCCUGUGCUGAAGGGCGUGAAACUGCAUUAUACC

>BNT: (MFE -1220.1 kcal/mol, CAI 0.946)
AUGUUCGUGUUCCUGGUGCUGCUGCCUCUGGUGUCCAGCCAGUGUGUGAACCUGACCACCAGAACACAGCUGCCUCCAGCCUACACCAACAGCUUUACCAGAGGCGU
GUACUACCCCGACAAGGUGUUCAGAUCCAGCGUGCUGCACUCUACCCAGGACCUGUUCCUGCCUUUCUUCAGCAACGUGACCUGGUUCCACGCCAUCCACGUGUCCG
GCACCAAUGGCACCAAGAGAUUCGACAACCCCGUGCUGCCCUUCAACGACGGGGUGUACUUUGCCAGCACCGAGAAGUCCAACAUCAUCAGAGGCUGGAUCUUCGGC
ACCACACUGGACAGCAAGACCCAGAGCCUGCUGAUCGUGAACAACGCCACCAACGUGGUCAUCAAAGUGUGCGAGUUCCAGUUCUGCAACGACCCCUUCCUGGGCGU
CUACUACCACAAGAACAACAAGAGCUGGAUGGAAAGCGAGUUCCGGGUGUACAGCAGCGCCAACAACUGCACCUUCGAGUACGUGUCCCAGCCUUUCCUGAUGGACC
UGGAAGGCAAGCAGGGCAACUUCAAGAACCUGCGCGAGUUCGUGUUUAAGAACAUCGACGGCUACUUCAAGAUCUACAGCAAGCACACCCCUAUCAACCUCGUGCGG
GAUCUGCCUCAGGGCUUCUCUGCUCUGGAACCCCUGGUGGAUCUGCCCAUCGGCAUCAACAUCACCCGGUUUCAGACACUGCUGGCCCUGCACAGAAGCUACCUGAC
ACCUGGCGAUAGCAGCAGCGGAUGGACAGCUGGUGCCGCCGCUUACUAUGUGGGCUACCUGCAGCCUAGAACCUUCCUGCUGAAGUACAACGAGAACGGCACCAUCA
CCGACGCCGUGGAUUGUGCUCUGGAUCCUCUGAGCGAGACAAAGUGCACCCUGAAGUCCUUCACCGUGGAAAAGGGCAUCUACCAGACCAGCAACUUCCGGGUGCAG
CCCACCGAAUCCAUCGUGCGGUUCCCCAAUAUCACCAAUCUGUGCCCCUUCGGCGAGGUGUUCAAUGCCACCAGAUUCGCCUCUGUGUACGCCUGGAACCGGAAGCG
GAUCAGCAAUUGCGUGGCCGACUACUCCGUGCUGUACAACUCCGCCAGCUUCAGCACCUUCAAGUGCUACGGCGUGUCCCCUACCAAGCUGAACGACCUGUGCUUCA
CAAACGUGUACGCCGACAGCUUCGUGAUCCGGGGAGAUGAAGUGCGGCAGAUUGCCCCUGGACAGACAGGCAAGAUCGCCGACUACAACUACAAGCUGCCCGACGAC
UUCACCGGCUGUGUGAUUGCCUGGAACAGCAACAACCUGGACUCCAAAGUCGGCGGCAACUACAAUUACCUGUACCGGCUGUUCCGGAAGUCCAAUCUGAAGCCCUU
CGAGCGGGACAUCUCCACCGAGAUCUAUCAGGCCGGCAGCACCCCUUGUAACGGCGUGGAAGGCUUCAACUGCUACUUCCCACUGCAGUCCUACGGCUUUCAGCCCA
CAAAUGGCGUGGGCUAUCAGCCCUACAGAGUGGUGGUGCUGAGCUUCGAACUGCUGCAUGCCCCUGCCACAGUGUGCGGCCCUAAGAAAAGCACCAAUCUCGUGAAG
AACAAAUGCGUGAACUUCAACUUCAACGGCCUGACCGGCACCGGCGUGCUGACAGAGAGCAACAAGAAGUUCCUGCCAUUCCAGCAGUUUGGCCGGGAUAUCGCCGA
UACCACAGACGCCGUUAGAGAUCCCCAGACACUGGAAAUCCUGGACAUCACCCCUUGCAGCUUCGGCGGAGUGUCUGUGAUCACCCCUGGCACCAACACCAGCAAUC
AGGUGGCAGUGCUGUACCAGGACGUGAACUGUACCGAAGUGCCCGUGGCCAUUCACGCCGAUCAGCUGACACCUACAUGGCGGGUGUACUCCACCGGCAGCAAUGUG
UUUCAGACCAGAGCCGGCUGUCUGAUCGGAGCCGAGCACGUGAACAAUAGCUACGAGUGCGACAUCCCCAUCGGCGCUGGAAUCUGCGCCAGCUACCAGACACAGAC
AAACAGCCCUCGGAGAGCCAGAAGCGUGGCCAGCCAGAGCAUCAUUGCCUACACAAUGUCUCUGGGCGCCGAGAACAGCGUGGCCUACUCCAACAACUCUAUCGCUA
UCCCCACCAACUUCACCAUCAGCGUGACCACAGAGAUCCUGCCUGUGUCCAUGACCAAGACCAGCGUGGACUGCACCAUGUACAUCUGCGGCGAUUCCACCGAGUGC

```
UCCAACCUGCUGCUGCAGUACGGCAGCUUCUGCACCCAGCUGAAUAGAGCCCUGACAGGGAUCGCCGUGGAACAGGACAAGAACACCCAAGAGGUGUUCGCCCAAGU
GAAGCAGAUCUACAAGACCCCUCCUAUCAAGGACUUCGGCGGCUUCAAUUUCAGCCAGAUUCUGCCCGAUCCUAGCAAGCCCAGCAAGCGGAGCUUCAUCGAGGACC
UGCUGUUCAACAAAGUGACACUGGCCGACGCCGGCUUCAUCAAGCAGUAUGGCGAUUGUCUGGGCGACAUUGCCGCCAGGGAUCUGAUUUGCGCCCAGAAGUUUAAC
GGACUGACAGUGCUGCCUCCUCUGCUGACCGAUGAGAUGAUCGCCCAGUACACAUCUGCCCUGCUGGCCGGCACAAUCACAAGCGGCUGGACAUUUGGAGCAGGCGC
CGCUCUGCAGAUCCCCUUUGCUAUGCAGAUGGCCUACCGGUUCAACGGCAUCGGAGUGACCCAGAAUGUGCUGUACGAGAACCAGAAGCUGAUCGCCAACCAGUUCA
ACAGCGCCAUCGGCAAGAUCCAGGACAGCCUGAGCAGCACAGCAAGCGCCCUGGGAAAGCUGCAGGACGUGGUCAACCAGAAUGCCCAGGCACUGAACACCCUGGUC
AAGCAGCUGUCCUCCAACUUCGGCGCCAUCAGCUCUGUGCUGAACGAUAUCCUGAGCAGACUGGACaaagUgGAGGCCGAGGUGCAGAUCGACAGACUGAUCACAGG
CAGACUGCAGAGCCUCCAGACAUACGUGACCCAGCAGCUGAUCAGAGCCGCCGAGAUUAGAGCCUCUGCCAAUCUGGCCGCCACCAAGAUGUCUGAGUGUGUGCUGG
GCCAGAGCAAGAGAGUGGACUUUUGCGGCAAGGGCUACCACCUGAUGAGCUUCCCUCAGUCUGCCCCUCACGGCGUGGUGUUUCUGCACGUGACAUAUGUGCCCGCU
CAAGAGAAGAAUUUCACCACCGCUCCAGCCAUCUGCCACGACGGCAAAGCCCACUUUCCUAGAGAAGGCGUGUUCGUGUCCAACGGCACCCAUUGGUUCGUGACACA
GCGGAACUUCUACGAGCCCCAGAUCAUCACCACCGACAACACCUUCGUGUCUGGCAACUGCGACGUCGUGAUCGGCAUUGUGAACAAUACCGUGUACGACCCUCUGC
AGCCCGAGCUGGACAGCUUCAAAGAGGAACUGGACAAGUACUUUAAGAACCACACAAGCCCCGACGUGGACCUGGGCGAUAUCAGCGGAAUCAAUGCCAGCGUCGUG
AACAUCCAGAAAGAGAUCGACCGGCUGAACGAGGUGGCCAAGAAUCUGAACGAGAGCCUGAUCGACCUGCAAGAACUGGGGAAGUACGAGCAGUACAUCAAGUGGCC
CUGGUACAUCUGGCUGGGCUUUAUCGCCGGACUGAUUGCCAUCGUGAUGGUCACAAUCAUGCUGUGUUGCAUGACCAGCUGCUGUAGCUGCCUGAAGGGCUGUUGUA
GCUGUGGCAGCUGCUGCAAGUUCGACGAGGACGAUUCUGAGCCCGUGCUGAAGGGCGUGAAACUGCACUACACA
```

The 5’ and 3’ UTR sequences used in the COVID mRNA vaccine experiments are listed below.

```
> 5’ UTR
GGGAAAAAGAGAGAAAAGAAGAGAAGAAGAAAAAAGAGCCACC
> 3’ UTR
GCUGCCUUCUGCGGGGCUUGCCUUCUGGCCAUGCCCUUCUUCUCUCCCUUGCACCUGUACCUCUUGGUCUUUGAAUAAAGCCUGAGUAGGAAGU
```

Sequences used in VZV mRNA vaccine experiments

The mRNA sequences (coding region) used in the VZV mRNA vaccine experiments are listed below (stop codons not included). All sequences encode VZV gE protein. gE A–E are our designed mRNA sequences, gE-Ther is the baseline sequence designed by conventional codon optimization method, and gE-WT is the wildtype sequence. In the experiments, we use one stop codon `UGA` for all sequences.

```
>gE-A: (MFE -1145.60 kcal/mol, CAI 0.787)
AUGGGAACAGUAAACAAGCCGGUGGUUGGUGUGCUGAUGGGUUUCGGUAUCAUUACCGGAACCCUUCGGAUCACCAACCCUGUUCGUGCCAGCGUCCUCCGAUAUGA
UGACUUUCAUAUCGACGAGGACAAGCUGGACACGAACAGUGUCUAUGAGCCGUACUACCACUCCGACCAUGCGGAGAGUUCCUGGGUCAAUCGUGGGGAGAGUAGUC
GUAAGGCUUACGACCACAACUCUCCCUACAUUUGGCCCAGGAACGACUAUGACGGGUUCCUGGAGAACGCCCAUGAGCAUCAUGGGGUUUACAACCAGGGACGCGGC
AUAGACUCGGGCGAGAGGCUCAUGCAGCCAACCCAGAUGAGCGCGCAGGAGGAUCUCGGUGACGACACCGGGAUCCACGUCAUCCCCACUCUGAACGGUGACGACAG
GCACAAGAUUGUGAAUGUCGAUCAGCGUCAGUAUGGGGAUGUGUUCAAGGGAGACCUCAAUCCCAAGCCGCAGGGCCAGCGGCUGAUUGAGGUCUCCGUGGAGGAGA
ACCAUCCCUUCACCCUGCGCGCUCCCAUCCAGCGGAUCUACGGGGUCCGGUAUACAGAGACGUGGAGUUUUCUCCCGUCUCUGACUUGUACUGGCGAUGCAGCGCCA
GCGAUCCAGCACAUCUGCCUGAAGCACACCACGUGCUUCCAGGAUGUGGUGGUGGACGUGGACUGCGCGGAGAACACGAAGGAGGAUCAGCUUGCUGAGAUUAGCUA
CAGAUUCCAGGGCAAGAAGGAGGCCGACCAGCCCUGGAUUGUGGUUAAUACCAGCACGCUGUUCGACGAGCUGGAGCUCGAUCCUCCUGAGAUCGAGCCCGGCGUCC
UCAAGGUGCUGCGGACCGAGAAGCAGUACCUUGGGGUCUACAUAUGGAACAUGCGUGGAAGUGACGGCACUUCCACGUAUGCCACUUUUCUCGUCACCUGGAAAGGU
GACGAGAAGACUCGGAACCCCACGCCGGCUGUGACCCCACAGCCGCGUGGGGCCGAGUUCCAUAUGUGGAACUAUCACUCCCACGUGUUCUCCGUGGGGGACACGUU
CUCGCUGGCGAUGCAUCUGCAGUACAAGAUACACGAGGCCCCGUUCGAUCUGCUGCUGGAGUGGCUGUAUGUGCCGAUCGACCCGACCUGUCAGCCGAUGCGGCUGU
ACAGCACCUGCCUGUACCACCCGAACGCUCCUCAGUGUCUCAGCCACAUGAACAGUGGCUGCACUUUCACGAGUCCUCACCUGGCCCAGAGAGUGGCCAGUACGGUG
UAUCAGAACUGCGAGCACGCAGACAACUAUACAGCGUACUGUCUGGGGAUCAGCCACAUGGAGCCCAGCUUCGGGCUGAUCCUCCACGACGGGGGUACCACGUUGAA
GUUCGUGGAUACCCCCGAGUCACUCUCUGGGCUCUACGUGUUCGUUGUGUACUUCAACGGACACGUGGAGGCGGUGGCGUACACGGUGGUCAGCACCGUUGACCACU
```

UCGUGAACGCCAUCGAGGAGCGGGGGUUUCCUCCUACGGCUGGUCAGCCCCCGGCUACGACCAAGCCGAAGGAGAUAACCCCCGUUAACCCAGGCACAAGUCCACUG
CUGCGGUACGCCGCCUGGACAGGCGGCCUGGCCGCAGUAGUGCUCUUGUGCCUGGUGAUAUUCCUGAUCUGCACCGCCAAGCGGAUGCGGGUCAAGGCUUAUCGCGU
CGACAAGAGCCCGUAUAAUCAGAGCAUGUAUUAUGCGGGCUUGCCUGUCGACGAUUUUGAGGACUCGGAGAGCACAGACACUGAGGAGGAGUUCGGGAAUGCUAUAG
GCGGGUCGCAUGGUGGGAGUAGUUAUACGGUCUACAUAGACAAGACCCGG

>gE-B: (MFE -1082.9 kcal/mol, CAI 0.844)
AUGGGAACAGUAAACAAGCCCGUGGUCGGAGUUCUGAUGGGAUUCGGAAUAAUCACAGGCACGCUCCGGAUUACCAAUCCGGUGCGUGCCUCUGUCCUCCGGUAUGA
UGACUUUCAUAUCGACGAGGACAAGCUGGACACCAACAGCGUGUACGAGCCCUAUUACCACUCCGACCACGCGGAGUCCUCGUGGGUCAACCGCGGGGAGUCCUCCA
GAAAGGCCUAUGACCACAAUAGCCCUUACAUCUGGCCCCGGAACGAUUACGACGGCUUCCUGGAGAACGCCCACGAGCACCACGGGGUGUACAACCAGGGCCGCGGA
AUCGAUUCCGGGGAGAGGCUGAUGCAGCCUACCCAGAUGUCCGCCCAGGAGGACCUGGGCGACGACACUGGGAUCCAUGUCAUCCCCACCCUGAAUGGGGAUGACAG
GCACAAGAUCGUGAACGUGGACCAGCGGCAGUACGGGGACGUGUUCAAGGGAGACCUCAAUCCCAAGCCGCAGGGCCAGCGGCUGAUUGAGGUCUCCGUGGAGGAGA
ACCACCCCUUCACGCUGCGGGCCCCCAUUCAGCGCAUCUACGGGGUGCGGUACACCGAGACAUGGAGCUUCCUGCCAAGCCUCACAUGUACCGGUGAUGCCGCACCC
GCCAUCCAGCACAUCUGCCUGAAGCACACCACGUGCUUCCAGGAUGUGGUGGUGGACGUGGAUUGCGCUGAGAAUACCAAGGAGGAUCAGCUGGCGGAGAUCUCCUA
UCGGUUCCAGGGGAAGAAGGAGGCGGACCAGCCGUGGAUCGUCGUCAACACAAGCACCUUGUUUGACGAGCUGGAGCUCGAUCCUCCUGAGAUCGAGCCCGGCGUGC
UGAAGGUGCUGCGCACCGAGAAGCAGUAUCUCGGUGUGUACAUCUGGAACAUGCGUGGGAGUGACGGUACCUCCACAUAUGCCACCUUUCUCGUCACCUGGAAAGGU
GACGAGAAGACUCGGAACCCCACGCCGGCUGUGACCCCACAGCCGCGUGGGGCCGAGUUCCAUAUGUGGAACUAUCACUCCCACGUGUUCAGCGUGGGAGAUACCUU
CUCCCUCGCUAUGCACCUUCAGUACAAGAUCCACGAGGCCCCCUUCGAUCUUCUCCUGGAGUGGCUGUACGUGCCCAUUGACCCCACGUGCCAGCCUAUGAGGCUGU
ACAGCACGUGCCUGUACCACCCGAACGCGCCACAGUGCCUGAGUCACAUGAAUUCUGGCUGCACCUUUACUAGCCCCCAUCUGGCCCAGAGGGUGGCUAGUACGGUG
UACCAGAAUUGUGAGCAUGCAGACAACUAUACAGCCUAUUGCCUGGGCAUCAGCCACAUGGAGCCCAGCUUUGGGCUGAUUCUGCAUGACGGGGUACCACACUGAA
GUUUGUGGAUACCCCCGAGUCCCUCUCAGGCCUGUACGUGUUCGUGGUGUACUUCAAUGGGCACGUGGAGGCCGUGGCGUACACAGUGGUCAGCACCGUUGACCACU
UCGUGAACGCCAUCGAGGAGCGGGGGUUCCCGCCCACGGCGGGACAGCCCCCCGCUACCACGAAGCCGAAGGAGAUCACGCCAGUGAAUCCUGGUACCAGUCCACUG
CUGCGGUACGCAGCGUGGACUGGGGGGCUCGCAGCGGUGGUUCUCCUCUGCCUGGUGAUCUUCCUGAUCUGCACCGCCAAGCGGAUGCGGGUCAAGGCUUACCGGGU
GGACAAGUCCCCGUACAACCAGUCCAUGUAUUACGCAGGACUCCCCGUGGACGACUUCGAGGACUCCGAGUCUACCGACACCGAGGAAGAAUUCGGGAACGCCAUCG
GGGGUUCCCACGGGGGCUCCUCCUACACGGUGUACAUCGACAAGACUCGG

>gE-C: (MFE -932.3 kcal/mol, CAI 0.901)
AUGGGAACAGUAAACAAGCCCGUAGUAGGAGUGCUCAUGGGUUUCGGAAUCAUCACCGGAACCCUGAGAAUCACCAACCCAGUGCGGGCUUCUGUCCUGCGGUAUGA
UGACUUUCAUAUCGACGAGGACAAGCUGGACACCAACUCUGUGUACGAGCCCUACUACCACAGCGACCACGCUGAGUCCUCCUGGGUGAACCGGGGGGAGUCCUCCC
GGAAGGCCUAUGACCACAAUAGCCCUUACAUCUGGCCCCGGAACGAUUACGACGGCUUCCUGGAGAACGCCCACGAGCACCACGGGGUGUACAACCAGGGCCGCGGA
AUCGAUUCCGGGGAGAGGCUGAUGCAGCCUACCCAGAUGUCAGCCCAGGAGGACCUGGGCGACGACACAGGGAUCCAUGUCAUCCCCACCCUGAAUGGGGAUGACAG
GCACAAGAUUGUGAACGUCGAUCAGCGCCAGUAUGGGGAUGUGUUCAAGGGCGACCUGAACCCAAAGCCCCAGGGCCAGCGCCUGAUCGAGGUGAGCGUGGAGGAGA
ACCACCCAUUCACGCUGCGCGCACCGAUCCAGCGGAUCUACGGGGUGCGCUACACUGAGACCUGGAGCUUCCUGCCCAGUCUCACCUGCACUGGGGACGCAGCCCCC
GCAAUCCAGCACAUCUGCCUGAAGCACACCACGUGCUUCCAGGAUGUGGUGGUGGACGUGGACUGCGCCGAGAACACCAAGGAGGACCAGCUGGCCGAGAUCAGCUA
CCGCUUCCAGGGCAAGAAGGAGGCCGACCAGCCCUGGAUCGUGGUGAACACCAGCACCCUGUUCGACGAGCUGGAACUCGAUCCACCUGAGAUCGAGCCCGGCGUGU
UGAAGGUGCUGCGCACCGAGAAGCAGUAUCUCGGUGUGUACAUCUGGAAUAUGCGGGGGUCCGACGGGACCUCCACAUACGCCACCUUCUUGGUGACUUGGAAGGGU
GACGAGAAGACUAGAAACCCUACACCUGCUGUGACACCACAGCCAAGAGGGGCUGAGUUCCACAUGUGGAACUACCACAGCCACGUGUUUAGCGUGGGAGAUACCUU
CUCCCUCGCUAUGCACCUCCAGUACAAGAUCCACGAGGCCCCCUUCGAUCUGCUGCUGGAGUGGCUGUACGUGCCCAUCGACCCCACAUGUCAGCCAAUGAGGCUGU
ACAGCACAUGCCUGUACCACCCAAACGCACCACAGUGCCUGAGCCACAUGAACAGCGGGUGUACCUUCACCAGCCCUCAUCUGGCUCAGCGGGUGGCUAGCACGGUG
UACCAGAACUGCGAGCACGCAGACAACUACACCGCCUACUGCCUGGGGAUCAGCCACAUGGAGCCCAGCUUCGGGCUGAUCCUGCACGAUGGAGGCACCACGCUCAA
GUUCGUGGACACCCCCGAGAGCUUGAGCGGCCUCUACGUGUUCGUGGUCUACUUCAAUGGCCACGUGGAGGCCGUGGCCUACACCGUGGUCAGCACCGUGGACCACU
UCGUGAAUGCCAUCGAGGAGCGGGGCUUUCCUCCUACGGCCGGGCAGCCUCCCGCCACCACCAAGCCCAAGGAGAUCACCCCGGUGAAUCCGGGGACAUCUCCUCUG
CUGCGGUACGCUGCCUGGACCGGUGGGCUGGCAGCCGUGGUGCUGCUGUGCCUGGUCAUCUUCCUGAUCUGCACAGCAAAGCGCAUGCGGGUGAAGGCCUACCGGGU
GGACAAGAGUCCCUACAACCAGUCCAUGUACUACGCAGGACUGCCUGUGGACGACUUCGAGGACUCUGAGUCCACCGACACCGAGGAGGAGUUCGGCAACGCCAUCG
GUGGCAGCCACGGAGGGUCCAGCUACACCGUGUACAUCGACAAGACACGG

>gE-D: (MFE -845.4 kcal/mol, CAI 0.918)
AUGGGAACAGUAAAUAAGCCCGUAGUAGGAGUGCUGAUGGGCUUCGGCAUCAUCACCGGCACCCUGCGGAUAACCAACCCCGUGCGGGCCUCUGUCCUGCGGUAUGA
CGACUUCCAUAUCGACGAGGACAAGCUGGACACCAACUCUGUGUACGAGCCCUACUACCACAGCGACCACGCUGAGAGUAGCUGGGUGAACAGAGGGGAGUCCAGCC

GGAAGGCCUACGAUCACAAUAGCCCCUACAUCUGGCCCCGGAAUGACUACGAUGGGUUUCUGGAGAACGCCCACGAGCAUCACGGAGUGUACAACCAAGGAAGAGGC
AUUGAUUCAGGCGAGCGCCUGAUGCAGCCCACCCAGAUGUCCGCCCAGGAGGACCUGGGCGACGACACUGGGAUCCAUGUCAUCCCCACCCUGAAUGGGGAUGACAG
GCACAAGAUUGUGAACGUGGACCAGAGGCAGUACGGGGAUGUGUUCAAGGGCGAUCUGAAUCCUAAGCCCCAGGGGCAGAGACUGAUCGAGGUGAGCGUGGAGGAGA
ACCACCCAUUCACGCUGCGCGCACCAAUACAGCGGAUUUACGGGGUGCGCUACACCGAGACUUGGUCUUUCCUGCCCUCCCUGACCUGCACCGGGGACGCAGCCCCA
GCCAUCCAACACAUCUGCCUGAAGCACACCACCUGCUUCCAGGAUGUGGUGGUGGACGUGGACUGCGCCGAGAACACCAAGGAGGAUCAGCUGGCUGAGAUCUCCUA
CCGGUUCCAGGGGAAGAAGGAGGCAGAUCAGCCCUGGAUCGUGGUGAAUACCAGCACCCUGUUCGACGAGCUGGAGCUGGAUCCACCAGAGAUCGAGCCCGGGGUGC
UGAAGGUGCUGCGGACCGAGAAGCAGUACCUGGGAGUGUACAUCUGGAAUAUGCGGGGGUCCGACGGGACCUCCACAUACGCCACAUUCCUGGUCACCUGGAAGGGG
GACGAGAAGACUCGCAACCCAACUCCAGCAGUGACACCUCAGCCUCGGGGGGCUGAGUUCCACAUGUGGAACUACCACAGCCACGUGUUUAGCGUGGGAGAUACCUU
CUCCCUCGCUAUGCACCUCCAGUACAAGAUCCACGAGGCCCCCUUCGAUCUGCUGCUGGAGUGGCUGUACGUGCCCAUCGACCCCACAUGUCAGCCAAUGAGGCUGU
ACAGCACAUGCCUGUACCACCCAAACGCACCACAGUGCCUGAGCCACAUGAACAGUGGCUGCACUUUCACCAGCCCUCACCUGGCCCAGAGGGUGGCCUCCACCGUC
UACCAGAACUGUGAGCACGCUGACAACUACACAGCCUACUGCCUGGGCAUCAGCCACAUGGAGCCCAGCUUUGGGCUGAUCCUCCACGACGGGGGCACCACCCUGAA
GUUCGUGGACACCCCCGAGAGCCUGAGCGGGCUGUACGUGUUCGUGGUGUACUUCAACGGACACGUGGAGGCCGUGGCCUACACCGUGGUCUCCACCGUGGACCACU
UCGUGAAUGCCAUCGAGGAGCGGGGGUUCCCUCCUACCGCCGGGCAGCCUCCUGCCACCACCAAGCCCAAGGAGAUCACCCCCGUGAACCCAGGCACCAGUCCACUG
CUGCGGUACGCAGCAUGGACUGGGGGCCUGGCCGCCGUGGUGCUGCUGUGCCUGGUGAUCUUCCUCAUCUGCACCGCCAAGCGGAUGAGGGUGAAGGCUUACAGAGU
CGACAAGUCCCCCUACAACCAGUCCAUGUACUACGCAGGACUGCCUGUGGACGACUUCGAGGACUCUGAGAGCACCGACACCGAGGAGGAGUUCGGCAACGCCAUCG
GUGGCAGCCACGGAGGGUCCAGCUACACCGUGUACAUCGACAAGACACGG

>gE-E: (MFE -805.0 kcal/mol, CAI 0.930)

AUGGGAACAGUAAACAAGCCUGUGGUGGGCGUGCUGAUGGGCUUCGGCAUCAUCACAGGCACCCUGCGGAUCACCAACCCAGUGCGCGCCAGCGUGCUGCGGUACGA
UGACUUCCACAUCGACGAGGACAAGCUGGACACCAACAGCGUGUACGAGCCCUACUACCACUCCGACCACGCUGAGUCCAGCUGGGUGAACCGCGGGGAGAGCUCCA
GAAAGGCCUAUGACCACAAUAGCCCUUACAUCUGGCCCCGGAACGAUUACGACGGCUUCCUGGAGAACGCCCACGAGCACCACGGGGUGUACAACCAGGGCCGCGGC
AUCGAUUCCGGGGAGAGGCUGAUGCAGCCUACCCAGAUGUCCGCCCAGGAGGACCUGGGCGACGACACUGGGAUCCAUGUCAUCCCCACCCUGAACGGGGAUGACAG
GCACAAGAUCGUGAACGUGGACCAGCGCCAGUACGGCGAUGUGUUCAAGGGCGAUCUGAACCCCAAGCCCCAGGGGCAGCGCCUGAUCGAGGUGAGUGUGGAGGAGA
ACCACCCCUUCACACUGCGCGCUCCCAUCCAGCGGAUCUACGGCGUGCGGUACACAGAGACCUGGAGCUUCCUGCCCUCUCUGACCUGUACCGGCGACGCCGCCCCC
GCCAUCCAGCACAUCUGCCUGAAGCACACCACCUGCUUCCAGGAUGUGGUGGUGGAUGUGGACUGCGCCGAGAACACCAAGGAGGACCAGCUGGCCGAGAUCAGCUA
CCGCUUCCAGGGGAAGAAGGAGGCUGAUCAGCCCUGGAUCGUGGUGAACACAUCCACCCUGUUCGACGAGCUGGAGCUGGAUCCUCCUGAGAUCGAGCCCGGCGUAC
UCAAGGUACUGCGCACCGAAAAGCAGUACCUCGGUGUGUACAUCUGGAACAUGCGGGGCUCCGACGGGACUUCUACAUACGCCACCUUCCUGGUGACCUGGAAGGGC
GAUGAGAAGACAAGAAACCCAACACCAGCUGUGACACCACAGCCAAGAGGAGCCGAGUUCCACAUGUGGAACUACCACUCCCACGUGUUCAGCGUGGGGGACACCUU
CAGCCUGGCCAUGCAUCUGCAGUACAAGAUCCACGAGGCCCCCUUCGACCUGCUGCUGGAGUGGCUGUACGUGCCAAUCGACCCAACAUGCCAGCCAAUGCGGCUGU
ACAGCACAUGCCUGUACCACCCAAACGCACCACAGUGUCUGAGCCACAUGAACAGCGGGUGUACCUUCACCAGCCCCCACCUGGCUCAGAGGGUGGCCUCCACAGUG
UACCAGAACUGUGAGCACGCCGACAACUACACAGCCUACUGCCUGGGCAUCUCCCACAUGGAGCCCAGCUUUGGGCUGAUCCUGCACGACGGCGGGACCACCCUGAA
GUUCGUGGACACCCCUGAGAGCUUAUCGGGACUGUACGUAUUCGUAGUAUACUUCAACGGACACGUGGAGGCCGUGGCUUACACAGUGGUGUCCACAGUGGACCACU
UCGUGAACGCCAUCGAGGAGAGGGGGUUCCCCCCUACCGCCGGGCAGCCACCAGCAACAACAAAGCCAAAAGAGAUCACCCCCGUGAAUCCUGGGACUUCCCCCCUG
CUGCGGUACGCUGCCUGGACCGGCGGCCUGGCCGCCGUGGUGCUGCUGUGCCUGGUGAUCUUCCUGAUCUGCACCGCCAAGCGGAUGCGGGUGAAGGCUUACCGGGU
GGACAAGAGCCCCUACAACCAGUCCAUGUACUACGCUGGACUGCCCGUGGACGACUUCGAGGAUUCCGAGUCCACCGACACCGAGGAGGAGUUCGGAAACGCCAUCG
GAGGCAGCCACGGGGGCUCUAGCUACACCGUGUACAUCGACAAGACACGG

>gE-Ther: (MFE -592.2 kcal/mol, CAI 0.937)

AUGGGCACCGUGAACAAGCCUGUUGUGGGCGUGCUGAUGGGCUUCGGCAUCAUCACAGGCACCCUGCGGAUCACCAAUCCUGUGCGGGCUAGCGUGCUGAGAUACGA
CGACUUCCACAUCGACGAGGACAAGCUGGACACCAACAGCGUGUACGAGCCCUACUACCACAGCGAUCACGCCGAGUCUAGCUGGGUCAACAGAGGCGAGAGCAGCA
GAAAGGCCUACGACCACAACAGCCCCUACAUCUGGCCCCGGAACGACUACGAUGGCUUCCUGGAAAAUGCCCACGAGCACCACGGCGUGUACAAUCAAGGCAGAGGC
AUCGACAGCGGCGAGAGACUGAUGCAGCCUACACAGAUGAGCGCCCAAGAGGACCUGGGAGAUGAUACCGGCAUCCACGUGAUCCCCACACUGAACGGCGACGACAG
ACACAAGAUCGUGAACGUGGACCAGCGGCAGUACGGCGACGUGUUCAAGGGCGACCUGAAUCCUAAGCCUCAGGGCCAGCGCCUGAUCGAGGUGUCCGUGGAAGAGA
AUCACCCCUUCACACUGAGAGCCCCUAUCCAGAGAAUCUACGGCGUGCGCUAUACCGAGACAUGGUCCUUUCUGCCCAGCCUGACAUGUACCGGGGAUGCCGCUCCU
GCCAUCCAGCACAUUUGCCUGAAGCACACCACCUGUUUCCAGGACGUGGUGGUGGAUGUGGACUGCGCCGAGAACACCAAAGAGGAUCAGCUGGCCGAGAUCAGCUA
CCGGUUCCAGGGAAAGAAAGAGGCCGACCAGCCUUGGAUCGUGGUCAACACCAGCACACUGUUCGACGAGCUGGAACUGGACCCUCCUGAGAUUGAACCCGGGGUGC
UGAAGGUGCUGAGAACCGAGAAGCAGUACCUGGGAGUGUACAUCUGGAACAUGAGAGGCAGCGACGGCACCUCUACCUACGCCACCUUUCUGGUCACAUGGAAGGGC
GACGAGAAAACACGGAACCCCACACCAGCUGUGACCCCUCAACCUAGAGGCGCCGAGUUUCACAUGUGGAAUUACCACAGCCACGUGUUCAGCGUGGGCGAUACCUU

```
UAGCCUGGCCAUGCAUCUGCAGUACAAGAUCCACGAGGCCCCUUUCGACCUGCUGCUGGAAUGGCUGUACGUGCCCAUCGAUCCUACCUGCCAGCCUAUGCGGCUGU
ACUCCACCUGUCUGUAUCACCCCAACGCUCCCCAGUGCCUGAGCCACAUGAAUAGCGGCUGCACCUUCACAAGCCCUCACCUGGCUCAGCGAGUGGCCAGCACAGUG
UACCAGAAUUGCGAGCACGCCGACAAUUACACCGCCUACUGUCUGGGCAUCAGCCACAUGGAACCUAGCUUCGGCCUGAUCCUGCACGAUGGCGGCACAACCCUGAA
GUUCGUGGAUACCCCUGAGAGCCUGAGCGGCCUGUAUGUGUUCGUGGUGUACUUCAACGGCCACGUGGAAGCCGUGGCCUACACCGUGGUGUCUACCGUGGACCACU
UCGUGAACGCCAUCGAGGAAAGAGGCUUCCCUCCAACUGCUGGACAGCCUCCUGCCACCACCAAGCCUAAAGAAAUCACACCCGUGAAUCCCGGCACAAGCCCACUG
CUUAGAUACGCCGCUUGGACAGGCGGACUGGCUGCUGUUGUUCUGCUGUGCCUGGUCAUCUUCCUGAUCUGCACCGCCAAGCGGAUGAGAGUGAAGGCCUACAGAGU
GGACAAGAGCCCUUACAACCAGAGCAUGUACUACGCCGGCCUGCCUGUGGACGACUUCGAGGAUAGCGAGAGCACCGACACCGAGGAAGAGUUCGGCAACGCCAUUG
GAGGAUCUCACGGCGGCAGCAGCUAUACCGUGUACAUCGACAAGACCCGG
```

```
>gE-WT: (MFE -485.7 kcal/mol, CAI 0.656)
AUGGGGACAGUUAAUAAACCUGUGGUGGGGGUAUUGAUGGGGUUCGGAAUUAUCACGGGAACGUUGCGUAUAACGAAUCCGGUCAGAGCAUCCGUCUUGCGAUACGA
UGAUUUUCACAUCGAUGAAGACAAACUGGAUACAAACUCCGUAUAUGAGCCUUACUACCAUUCAGAUCAUGCGGAGUCUUCAUGGGUAAAUCGGGGAGAGUCUUCGC
GAAAAGCGUACGAUCAUAACUCACCUUAUAUAUGGCCACGUAAUGAUUAUGAUGGAUUUUUAGAGAACGCACACGAACACCAUGGGGUGUAUAAUCAGGGCCGUGGU
AUCGAUAGCGGGGAACGGUUAAUGCAACCCACACAAAUGUCUGCACAGGAGGAUCUUGGGGACGAUACGGGCAUCCACGUUAUCCCUACGUUAAACGGCGAUGACAG
ACAUAAAAUUGUAAAUGUGGACCAACGUCAAUACGGUGACGUGUUUAAAGGAGAUCUUAAUCCAAAACCCCAAGGCCAAAGACUCAUUGAGGUGUCAGUGGAAGAAA
AUCACCCGUUUACUUUACGCGCACCGAUUCAGCGGAUUUAUGGAGUCCGGUACACCGAGACUUGGAGCUUUUUGCCGUCAUUAACCUGUACGGGAGACGCAGCGCCC
GCCAUCCAGCAUAUAUGUUUAAAACAUACAACAUGCUUUCAAGACGUGGUGGUGGAUGUGGAUUGCGCGGAAAAUACUAAAGAGGAUCAGUUGGCCGAAAUCAGUUA
CCGUUUUCAAGGUAAGAAGGAAGCGGACCAACCGUGGAUUGUUGUAAACACGAGCACACUGUUUGAUGAACUCGAAUUAGACCCCCCCGAGAUUGAACCGGGUGUCU
UGAAAGUACUUCGGACAGAAAAACAAUACUUGGGUGUGUACAUUUGGAACAUGCGCGGCUCCGAUGGUACGUCUACCUACGCCACGUUUUUGGUCACCUGGAAAGGG
GAUGAAAAAACAAGAAACCCUACGCCCGCAGUAACUCCUCAACCAAGAGGGGCUGAGUUUCAUAUGUGGAAUUACCACUCGCAUGUAUUUUCAGUUGGUGAUACGUU
UAGCUUGGCAAUGCAUCUUCAGUAUAAGAUACAUGAAGCGCCAUUUGAUUUGCUGUUAGAGUGGUUGUAUGUCCCCAUCGAUCCUACAUGUCAACCAAUGCGGUUAU
AUUCUACGUGUUUGUAUCAUCCCAACGCACCCCAAUGCCUCUCUCAUAUGAAUUCCGGUUGUACAUUUACCUCGCCACAUUUAGCCCAGCGUGUUGCAAGCACAGUG
UAUCAAAAUUGUGAACAUGCAGAUAACUACACCGCAUAUUGUCUGGGAAUAUCUCAUAUGGAGCCUAGCUUUGGUCUAAUCUUACACGACGGGGGCACCACGUUAAA
GUUUGUAGAUACACCCGAGAGUUUGUCGGGAUUAUACGUUUUUGUGGUGUAUUUUAACGGGCAUGUUGAAGCCGUAGCAUACACUGUUGUAUCCACAGUAGAUCAUU
UUGUAAACGCAAUUGAAGAGCGUGGAUUUCCGCCAACGGCCGGUCAGCCACCGGCGACUACUAAACCCAAGGAAAUUACCCCCGUAAACCCCGGAACGUCACCACUU
CUACGAUAUGCCGCAUGGACCGGAGGGCUUGCAGCAGUAGUACUUUUAUGUCUCGUAAUAUUUUUAAUCUGUACGGCUAAACGAAUGAGGGUUAAAGCCUAUAGGGU
AGACAAGUCCCCGUAUAACCAAAGCAUGUAUUACGCUGGCCUUCCAGUGGACGAUUUCGAGGACUCGGAAUCUACGGAUACGGAAGAAGAGUUUGGUAACGCGAUUG
GAGGGAGUCACGGGGGUUCGAGUUACACGGUGUAUAUAGAUAAGACCCGG
```

The 5’ and 3’ UTR sequences used in the VZV mRNA vaccine experiments are listed below.

```
> 5’ UTR
AGGAAAUUCCAUUUGGCUGCAGCUUCUGGAGGGAGCCGACAGGAGACGUGGGGAGACGGCCACC
> 3’ UTR
GCUGCCUUCUGCGGGGCUUGCCUUCUGGCCAUGCCCUUCUUCUCUCCCUUGCACCUGUACCUCUUGGUCUUUGAAUAAAGCCUGAGUAGGAAGU
```
