# Supplementary material for: Algorithm for optimized mRNA design improves stability and immunogenicity
Source: Nature. 2023 May 2;621(7978):396–403. doi: 10.1038/s41586-023-06127-z (PMC10499610; doi:10.1038/s41586-023-06127-z)
Supplement: Supplementary file 2 — Reporting Summary [file 41586_2023_6127_MOESM2_ESM.pdf]

nature portfolio

Corresponding author(s): Liang Huang; Yujian Zhang; David H. Mathews; Hangwen Li

Last updated by author(s): Apr 6, 2023

Reporting Summary

Nature Portfolio wishes to improve the reproducibility of the work that we publish. This form provides structure for consistency and transparency in reporting. For further information on Nature Portfolio policies, see our Editorial Policies and the Editorial Policy Checklist.

Statistics

For all statistical analyses, confirm that the following items are present in the figure legend, table legend, main text, or Methods section.

n/a	Confirmed	
☐	☒	The exact sample size (*n*) for each experimental group/condition, given as a discrete number and unit of measurement
☐	☒	A statement on whether measurements were taken from distinct samples or whether the same sample was measured repeatedly
☐	☒	The statistical test(s) used AND whether they are one- or two-sided<br>*Only common tests should be described solely by name; describe more complex techniques in the Methods section.*
☒	☐	A description of all covariates tested
☒	☐	A description of any assumptions or corrections, such as tests of normality and adjustment for multiple comparisons
☐	☒	A full description of the statistical parameters including central tendency (e.g. means) or other basic estimates (e.g. regression coefficient) AND variation (e.g. standard deviation) or associated estimates of uncertainty (e.g. confidence intervals)
☐	☒	For null hypothesis testing, the test statistic (e.g. *F*, *t*, *r*) with confidence intervals, effect sizes, degrees of freedom and *P* value noted<br>*Give P values as exact values whenever suitable.*
☒	☐	For Bayesian analysis, information on the choice of priors and Markov chain Monte Carlo settings
☒	☐	For hierarchical and complex designs, identification of the appropriate level for tests and full reporting of outcomes
☒	☐	Estimates of effect sizes (e.g. Cohen's *d*, Pearson's *r*), indicating how they were calculated

Our web collection on statistics for biologists contains articles on many of the points above.

Software and code

Policy information about availability of computer code

Data collection	The LinearDesign source code is available to all parties on GitHub (https://github.com/LinearDesignSoftware/LinearDesign), and is free for academic and research use.
Data analysis	Clang (11.0.0) is used to compile LinearDesign source code. Vienna RNAfold from ViennaRNA package (version 2.4.14; open source) is used for predicting and drawing the secondary structure of mRNA sequence, and calculating the Minimum Free Energy (MFE) of secondary structures. For the wet lab experiments, GraphPad Prism 8.0 was used for the data analysis. Flow cytometry data were analyzed by FlowJo 10.1.

For manuscripts utilizing custom algorithms or software that are central to the research but not yet described in published literature, software must be made available to editors and reviewers. We strongly encourage code deposition in a community repository (e.g. GitHub). See the Nature Portfolio guidelines for submitting code & software for further information.

Data

Policy information about availability of data

All manuscripts must include a data availability statement. This statement should provide the following information, where applicable:

- Accession codes, unique identifiers, or web links for publicly available datasets
- A description of any restrictions on data availability
- For clinical datasets or third party data, please ensure that the statement adheres to our policy

The UniProt sequences used to estimate the time complexity of LinearDesign are included in Supplementary Tab. 1 and deposited at our figshare repository https://

doi.org/10.6084/m9.figshare.22193251. The COVID-19 and VZV mRNA coding region sequences and UTR sequences used in the biological experiments are included at the end of Supplementary Information file and available on our figshare repository. Source data of the animal experiments is provided with this paper, and all source data of wet lab experiments is available on that repository.

Human research participants

Policy information about studies involving human research participants and Sex and Gender in Research.

Reporting on sex and gender	N/A
Population characteristics	N/A
Recruitment	N/A
Ethics oversight	N/A

Note that full information on the approval of the study protocol must also be provided in the manuscript.

Field-specific reporting

Please select the one below that is the best fit for your research. If you are not sure, read the appropriate sections before making your selection.

☒ Life sciences ☐ Behavioural & social sciences ☐ Ecological, evolutionary & environmental sciences

For a reference copy of the document with all sections, see nature.com/documents/nr-reporting-summary-flat.pdf

Life sciences study design

All studies must disclose on these points even when the disclosure is negative.

Sample size	In the animal study, six mice (for COVID mRNA vaccine experiments) and five mice (for VZV mRNA vaccine experiments) were used in the corresponding experiments, respectively. The sample size of mice in each group was determined based on general animal study practice. Five or six mice per group were commonly used, which can also been seen in other publications (Nature 58, 567-571 (2020); Nat Commun 12, 2893 (2021); Molecular Therapy 29.6 (2021): 1970-1983.)
Data exclusions	There is no data exclusion in our study.
Replication	In vitro experiments were independently repeated in triplicate. All replication attempts were successful. Animal experiments were completed once. Gel electrophoresis experiments were repeated three times to obtain similar results.
Randomization	Animals were randomly allocated into each group. No specific randomization method was used. For other experiments, we performed side-by-side comparison at the same time to keep the experimental condition uniform. Therefore no randomization is needed.
Blinding	The investigators were not blinded to the data collection as all the assays were run by the same team that performed the animal immunization.

Reporting for specific materials, systems and methods

We require information from authors about some types of materials, experimental systems and methods used in many studies. Here, indicate whether each material, system or method listed is relevant to your study. If you are not sure if a list item applies to your research, read the appropriate section before selecting a response.

Materials & experimental systems

n/a	Involved in the study	
☐	☒	Antibodies
☐	☒	Eukaryotic cell lines
☒	☐	Palaeontology and archaeology
☐	☒	Animals and other organisms
☒	☐	Clinical data
☒	☐	Dual use research of concern

Methods

n/a	Involved in the study	
☒	☐	ChIP-seq
☐	☒	Flow cytometry
☒	☐	MRI-based neuroimaging

Antibodies

Antibodies used	anti-RBD Fc chimeric mAb (Cat: 40150-D001, Sino Biological) Clone #D001<br>PE-anti-human IgG Fc (Cat: 410707, Biolgend) Clone M1310G05<br>HRP-conjugated goat anti-mouse IgG Ab (Cat: 31430, Invitrogen) Polyclonal<br>Anti-VZV gE protein antibody (Cat: 272686, Abcam) Clone #9<br>Goat Anti-Mouse IgG H&L (PE)) (Cat: 97024, Abcam) Polyclonal<br>Goat Anti-Mouse IgG Fc (HRP) (Cat: 97265, Abcam) Polyclonal
Validation	anti-RBD Fc chimeric mAb:<br>Du L, et al. (2009) The spike protein of SARS-CoV--a target for vaccine and therapeutic development. Nat Rev Microbiol. 7 (3): 226-36.<br>Anti-VZV gE protein antibody:<br>Wu S et al. Transcriptome Analysis Reveals the Role of Cellular Calcium Disorder in Varicella Zoster Virus-Induced Post-Herpetic Neuralgia. Front Mol Neurosci 14:665931 (2021).

Eukaryotic cell lines

Policy information about cell lines and Sex and Gender in Research

Cell line source(s)	HEK-293 cell line from ATCC (Cat# CRL-1573™) was used.
Authentication	Cell line was not authenticated.
Mycoplasma contamination	The cells were tested negative for mycoplasma contamination. MycoBlue Mycoplasma Detector (Vazyme) was used for detection.
Commonly misidentified lines<br>(See ICLAC register)	No commonly misidentified cell lines were used.

Animals and other research organisms

Policy information about studies involving animals; ARRIVE guidelines recommended for reporting animal research, and Sex and Gender in Research

Laboratory animals	C57BL/6 mice (6-8 weeks, female) were used in this study. Mice were maintained on 12 h light:dark cycles with a housing temperature between 20–24 °C and 40-60% humidity.
Wild animals	The study did not involve wild animals.
Reporting on sex	Only female mice were used in this study without specific consideration of the sex impact on the results. Though publications have shown that male and female mice may differ in immune responses to vaccination (PNAS, 2018 Dec 4; 115(49): 12477–12482.). We followed a general practice using female mice in COVID-19 vaccine studies as used in other studies (Nature 586, 567–571 (2020); Cell 182, 1271–1283.e1–e7, September 3, 2020).
Field-collected samples	No field-collected samples were involved in this study.
Ethics oversight	All mice studies were performed in strict accordance with the guidelines set by the Chinese Regulations of Laboratory Animals and Laboratory Animal-Requirements of Environment and Housing Facilities. Animal experiments were carried out in compliance with the approval protocol from the Institutional Animal Care and Use Committee (IACUC) of Shanghai Model Organisms Center, Inc..

Note that full information on the approval of the study protocol must also be provided in the manuscript.

Flow Cytometry

Plots

Confirm that:

☒ The axis labels state the marker and fluorochrome used (e.g. CD4-FITC).

☒ The axis scales are clearly visible. Include numbers along axes only for bottom left plot of group (a 'group' is an analysis of identical markers).

☒ All plots are contour plots with outliers or pseudocolor plots.

☒ A numerical value for number of cells or percentage (with statistics) is provided.

Methodology

Sample preparation	Human embryonic kidney 293 cells (HEK293) (ATCC) were cultured in Dulbecco's Modified Eagle's Medium (DMEM) (Hyclone) containing 10% fetal bovine serum (FBS) (GEMINI) and 1% Penicillin-Streptomycin (Gibco). All cells were cultured at 37 °C in a 5% CO2 condition.<br>For the measurement of protein expression, cells were transfected with mRNA using Lipofectamine MessengerMAX (Thermo

Scientific). Briefly, a mix of 2 µg mRNA and 6 µL of Lipofectamine reagent was prepared following the manual instructions and then incubated with cells for 24 or 48 hours. For flow cytometric analysis, cells were collected and stained with live/dead cell dye (Fixable Viability Stain 510, BD) for 5 min. After washing, cells were incubated with anti-RBD chimeric mAb (1:100 dilution, Sino Biological) for 30 min, followed by washing and incubation with PE-anti-human IgG Fc (1:100 dilution, Biolgend) for 30 min. Samples were analyzed on BD Canto II (BD Biosciences). Data were processed using FlowJo V10.1 (Tree Star).

Instrument

BD FACSCanto II (Serial # : R33896203261).

Software

Flowjo version 10.1 was used in FACS analysis.

Cell population abundance

After gating the singlet cells, a total of 10,000 cells were collected for each independent assay.

Gating strategy

In our FACS experiments, only homogeneous cells (HEK293) were used for the evaluation of specific protein translation. In this case, only one fluorescent staining was used to assess the intensity. No other unique gating strategy was applied except for the exclusion of doublets and dead cells.

☐ Tick this box to confirm that a figure exemplifying the gating strategy is provided in the Supplementary Information.
